# Supplementary material for: Cross-trait multivariate GWAS confirms health implications of pubertal timing
Source: Nat Commun. 2025 Jan 18;16:799. doi: 10.1038/s41467-025-56191-4 (PMC11742396; doi:10.1038/s41467-025-56191-4)
Supplement: Supplementary file 1 — Supplementary Information [file 41467_2025_56191_MOESM1_ESM.pdf]

Supplementary information

Tables of contents

Supplementary Figures....3
Supplementary Methods....42
Genomic structural equation model background and overview....42
Structured covariance models and factor analysis....42
Assessing model fit. 43
Effective sample size calculation. 44
Heterogeneity testing (QSNP). 45
Genomic risk loci and functional annotation of GWAS results....45
Fine-mapping....45
Gene prioritization using transcriptomic imputation....47
Plasma protein prioritization using proteome imputation....49
Gene set enrichment....50
Significant Cross-trait OUtliers and Trends in JOint York regression....50
BrainXcan imputation....51
Linkage disequilibrium score (LDSC)....53
Mendelian randomization (MR)....54
Mendelian randomization assumptions. 54
MR with complex traits....54
Cancers....55
Cardiovascular diseases....55
Osteoporosis....56
Psychiatric diseases and neurodegenerative diseases....56
Renal disorders and gastrointestinal tract disorders....56
Metabolic syndrome....57
Aging related phenotypes....57
Organ volume/fat/iron....58
Polygenic Mendelian randomization methods....58
Sample independence....59
The mediation effect of mvPuberty to adult traits....59
MR with modifiable dietary factors....60
STROBE-MR Reporting Guidelines....64

Supplementary Disscussion..69
Supplementary References..72

Supplementary Figures

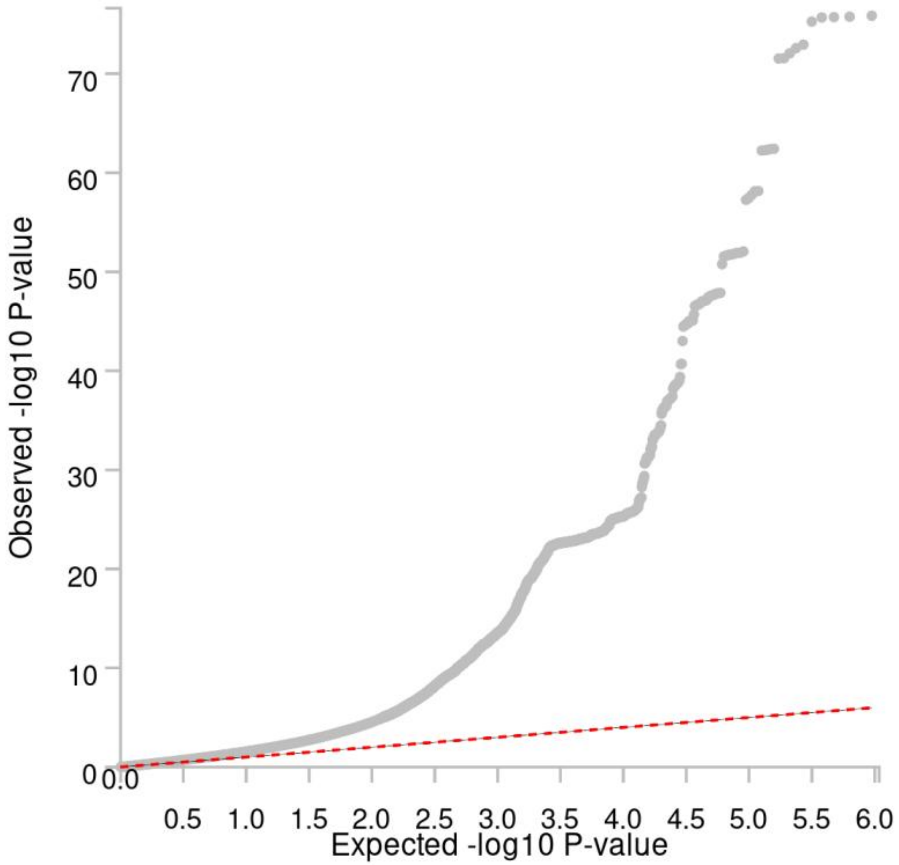

Supplementary Fig. 1. Multivariate GWAS Quantile-Quantile (Q-Q) plot.
Plot represents a comparison of the mvPuberty multivariate genome-wide association study (GWAS) (N=514,750); P values to those expected for null distribution (depicted by the red line). See the **Results** discussion for additional information.

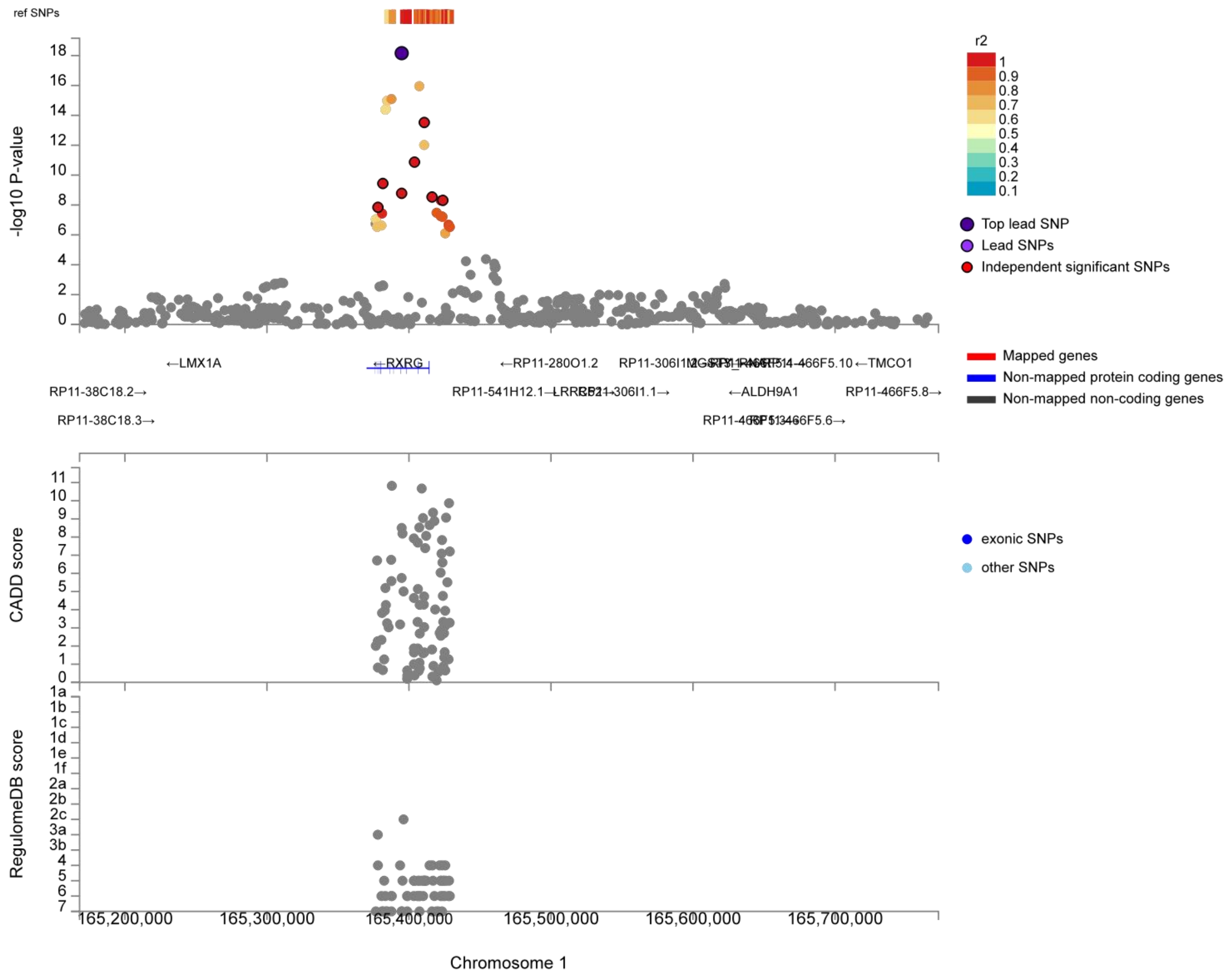

Supplementary Fig. 2. Regional association plot for SNP rs466639.

Regional plot of the locus around the lead variant rs466639 identified in the mvPuberty multivariate genome-wide association study (GWAS) (N=514,750). Top Top panel presents the -log10(P-values) results of two-sided Wald tests for each variant on mvPuberty and linkage disequilibrium (LD) R^2 information for the variants in the locus (variants are colored by LD R^2) and the genes prioritized by FUMA are highlighted in red on the track below.

Bottom The bottom panel presents the CADD (combined annotation dependent depletion) scores and RegulomeDB scores (top and bottom tracks, respectively).

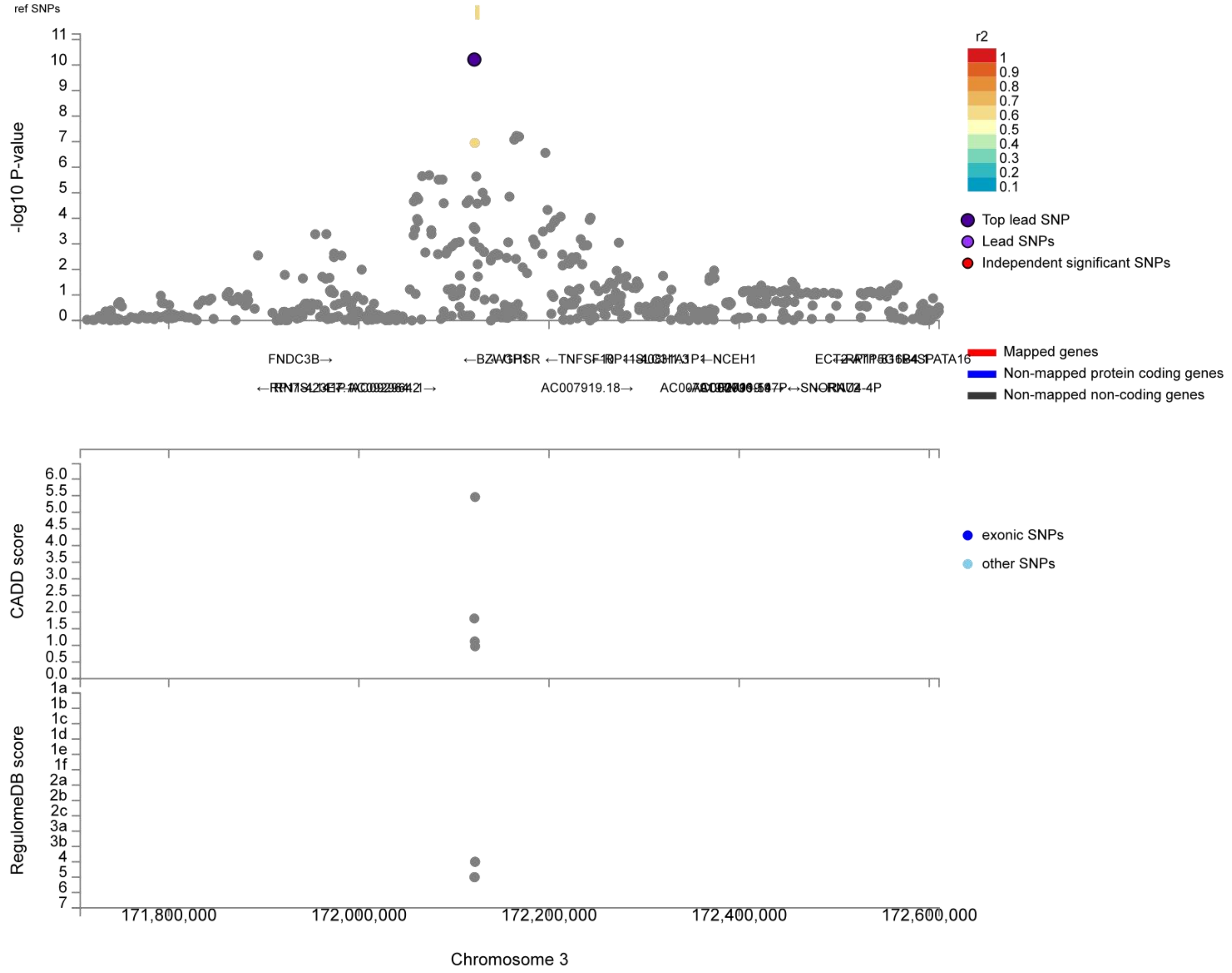

Supplementary Fig. 3. Regional association plot for SNP rs582780.

Regional plot of the locus around the lead variant rs582780 identified in the mvPuberty multivariate genome-wide association study (GWAS) (N=514,750). Top Top panel presents the -log10(P-values) results of two-sided Wald tests for each variant on mvPuberty and linkage disequilibrium (LD) R^2 information for the variants in the locus (variants are colored by LD R^2) and the genes prioritized by FUMA are highlighted in red on the track below.

Bottom The bottom panel presents the CADD (combined annotation dependent depletion) scores and RegulomeDB scores (top and bottom tracks, respectively).

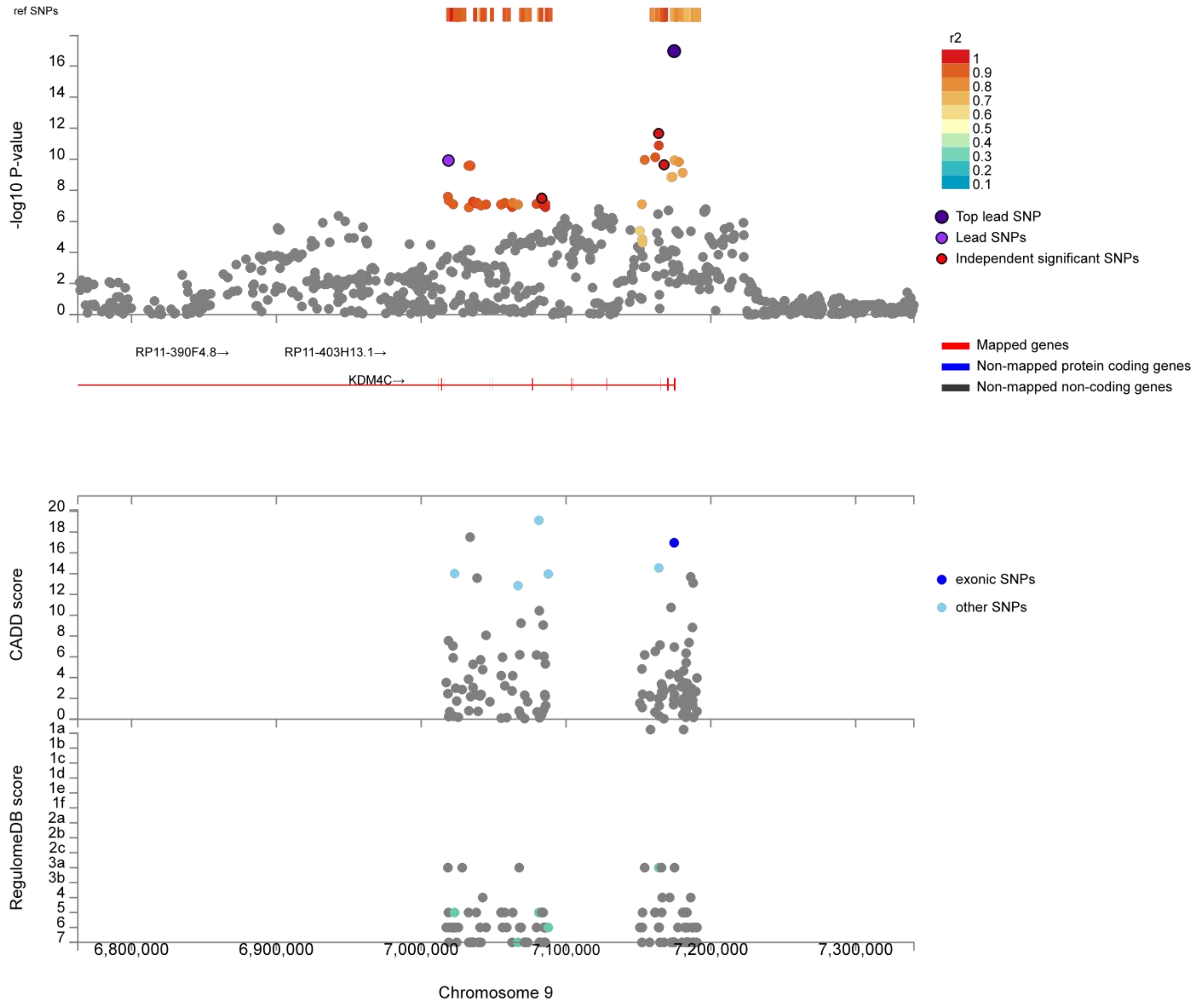

Supplementary Fig. 4. Regional association plot for SNP rs913588.
Regional plot of the locus around the lead variant rs913588 identified in the mvPuberty multivariate genome-wide association study (GWAS) (N=514,750). Top Top panel presents the -log10(P-values) results of two-sided Wald tests for each variant on mvPuberty and linkage disequilibrium (LD) R^2 information for the variants in the locus (variants are colored by LD R^2) and the genes prioritized by FUMA are highlighted in red on the track below.
Bottom The bottom panel presents the CADD (combined annotation dependent depletion) scores and RegulomeDB scores (top and bottom tracks, respectively).

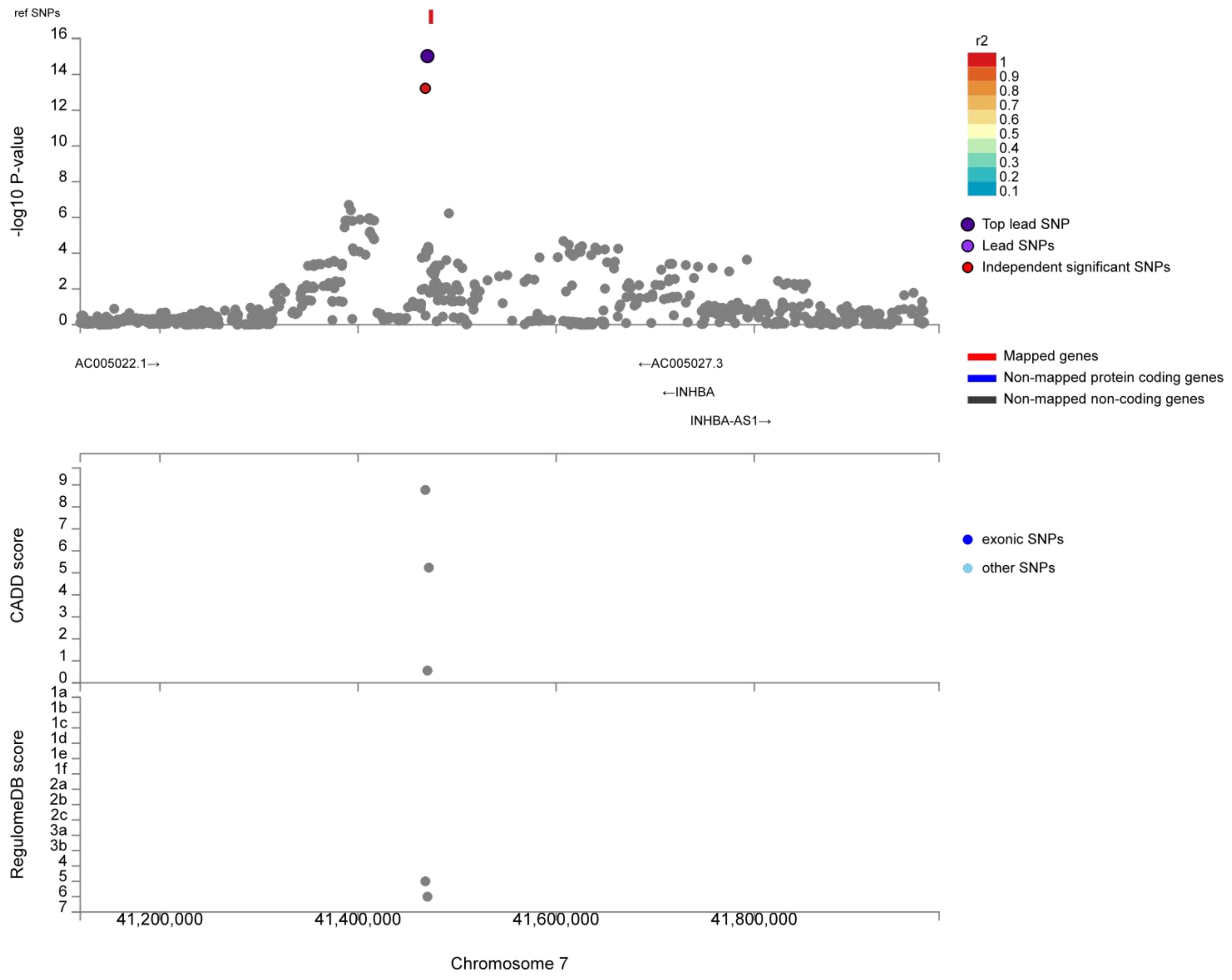

Supplementary Fig. 5. Regional association plot for SNP rs1079866.
Regional plot of the locus around the lead variant rs1079866 identified in the mvPuberty multivariate genome-wide association study (GWAS) (N=514,750). Top Top panel presents the -log10(P-values) results of two-sided Wald tests for each variant on mvPuberty and linkage disequilibrium (LD) R^2 information for the variants in the locus (variants are colored by LD R^2) and the genes prioritized by FUMA are highlighted in red on the track below.
Bottom The bottom panel presents the CADD (combined annotation dependent depletion) scores and RegulomeDB scores (top and bottom tracks, respectively).

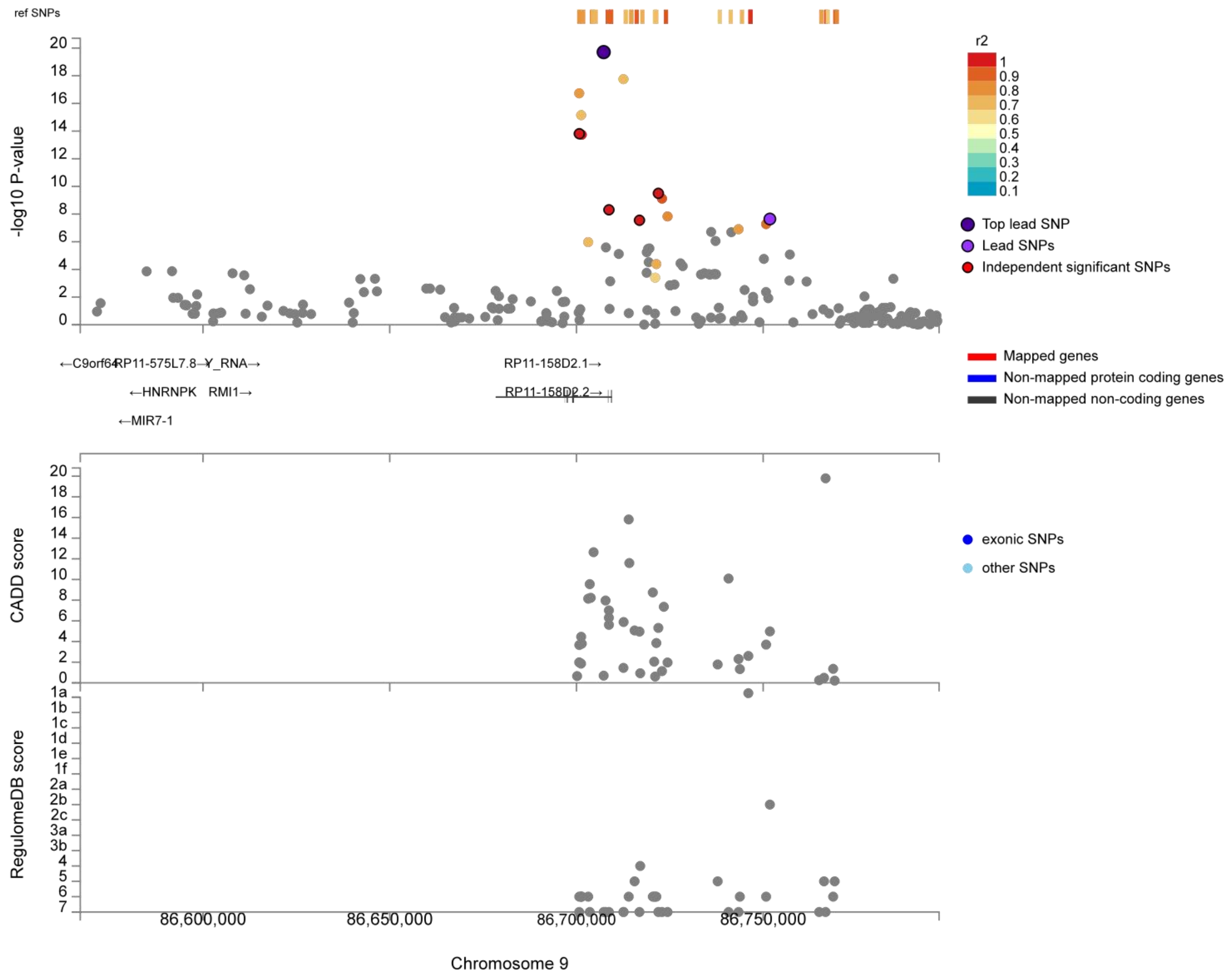

Supplementary Fig. 6. Regional association plot for SNP rs2378662.

Regional plot of the locus around the lead variant rs2378662 identified in the mvPuberty multivariate genome-wide association study (GWAS) (N=514,750). Top Top panel presents the -log10(P-values) results of two-sided Wald tests for each variant on mvPuberty and linkage disequilibrium (LD) R^2 information for the variants in the locus (variants are colored by LD R^2) and the genes prioritized by FUMA are highlighted in red on the track below.

Bottom The bottom panel presents the CADD (combined annotation dependent depletion) scores and RegulomeDB scores (top and bottom tracks, respectively).

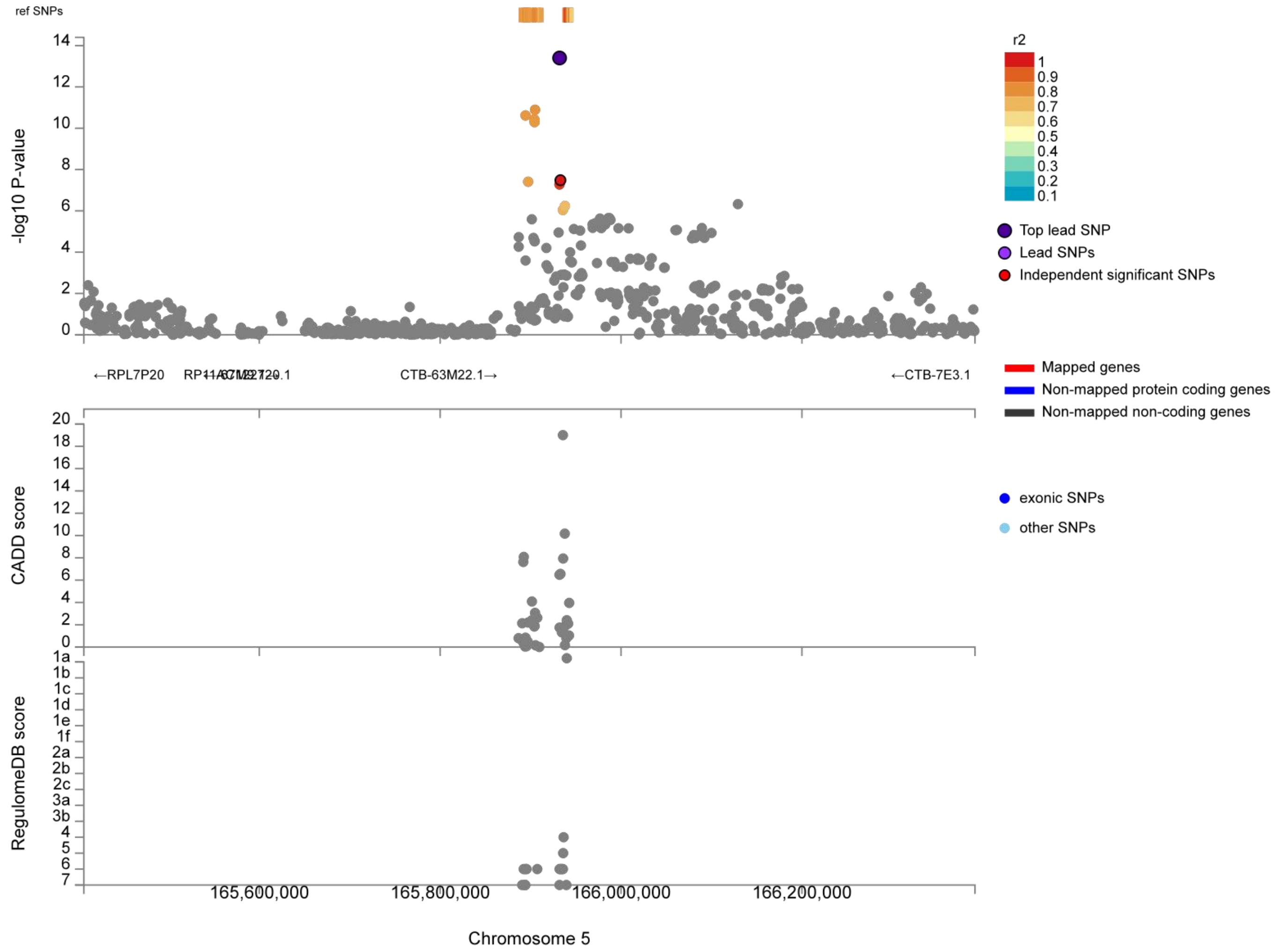

Supplementary Fig. 7. Regional association plot for SNP rs2961853.

Regional plot of the locus around the lead variant rs2961853 identified in the mvPuberty multivariate genome-wide association study (GWAS) (N=514,750). Top Top panel presents the -log10(P-values) results of two-sided Wald tests for each variant on mvPuberty and linkage disequilibrium (LD) R^2 information for the variants in the locus (variants are colored by LD R^2) and the genes prioritized by FUMA are highlighted in red on the track below.

Bottom The bottom panel presents the CADD (combined annotation dependent depletion) scores and RegulomeDB scores (top and bottom tracks, respectively).

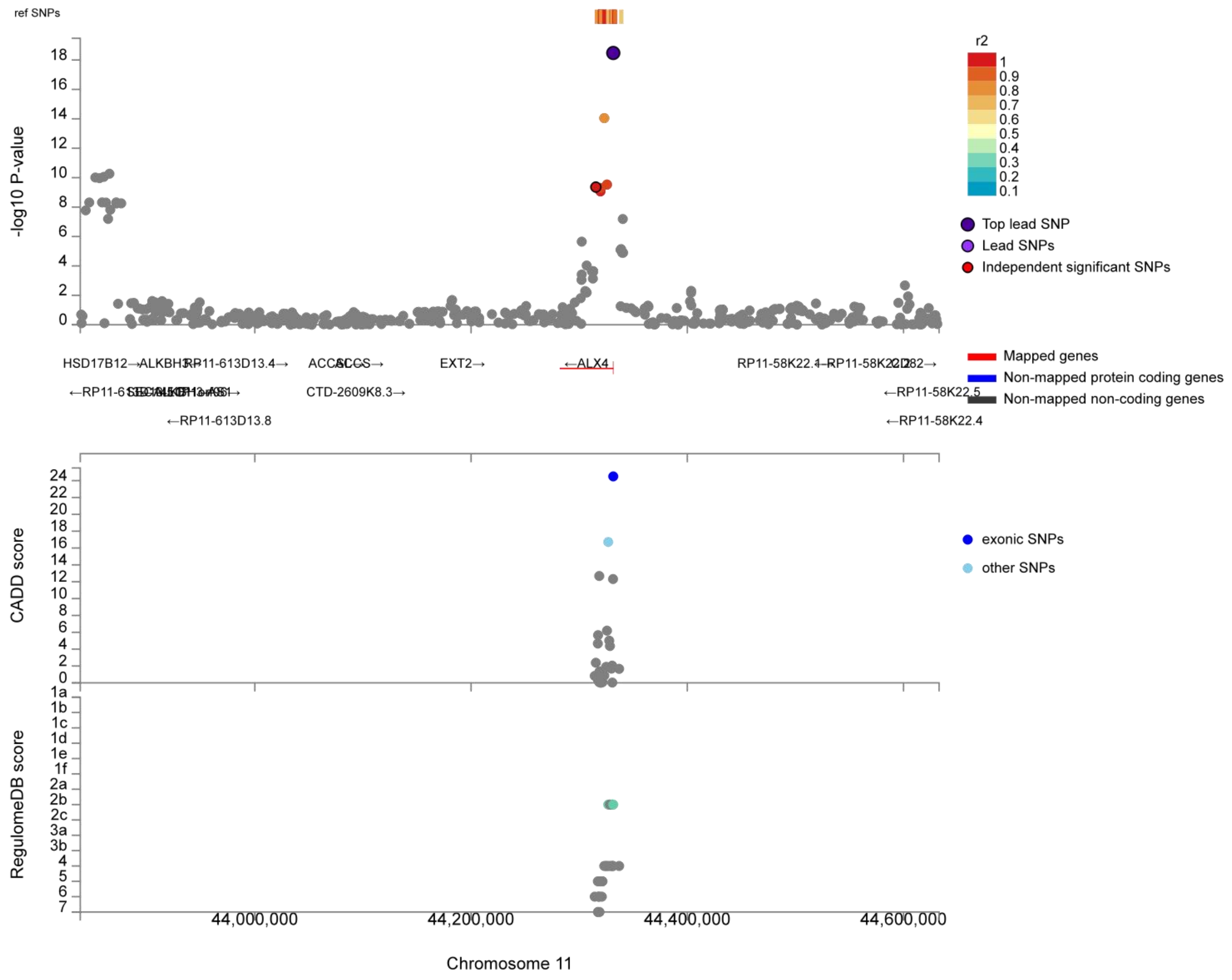

Supplementary Fig. 8. Regional association plot for SNP rs3824915.

Regional plot of the locus around the lead variant rs3824915 identified in the mvPuberty multivariate genome-wide association study (GWAS) (N=514,750). Top Top panel presents the -log10(P-values) results of two-sided Wald tests for each variant on mvPuberty and linkage disequilibrium (LD) R^2 information for the variants in the locus (variants are colored by LD R^2) and the genes prioritized by FUMA are highlighted in red on the track below.

Bottom The bottom panel presents the CADD (combined annotation dependent depletion) scores and RegulomeDB scores (top and bottom tracks, respectively).

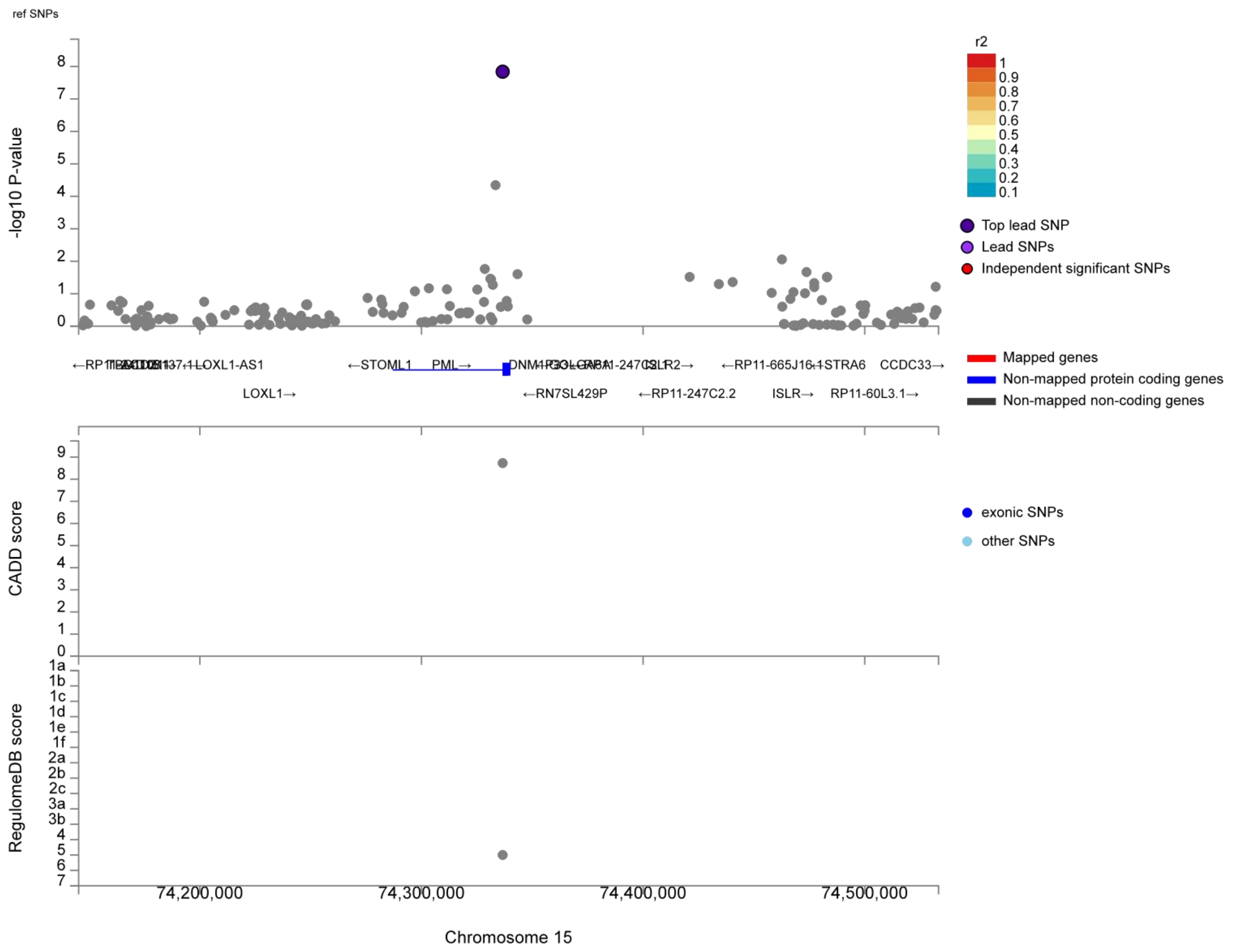

Supplementary Fig. 9. Regional association plot for SNP rs5742915.

Regional plot of the locus around the lead variant rs5742915 identified in the mvPuberty multivariate genome-wide association study (GWAS) (N=514,750). Top Top panel presents the -log10(P-values) results of two-sided Wald tests for each variant on mvPuberty and linkage disequilibrium (LD) R^2 information for the variants in the locus (variants are colored by LD R^2) and the genes prioritized by FUMA are highlighted in red on the track below.

Bottom The bottom panel presents the CADD (combined annotation dependent depletion) scores and RegulomeDB scores (top and bottom tracks, respectively).

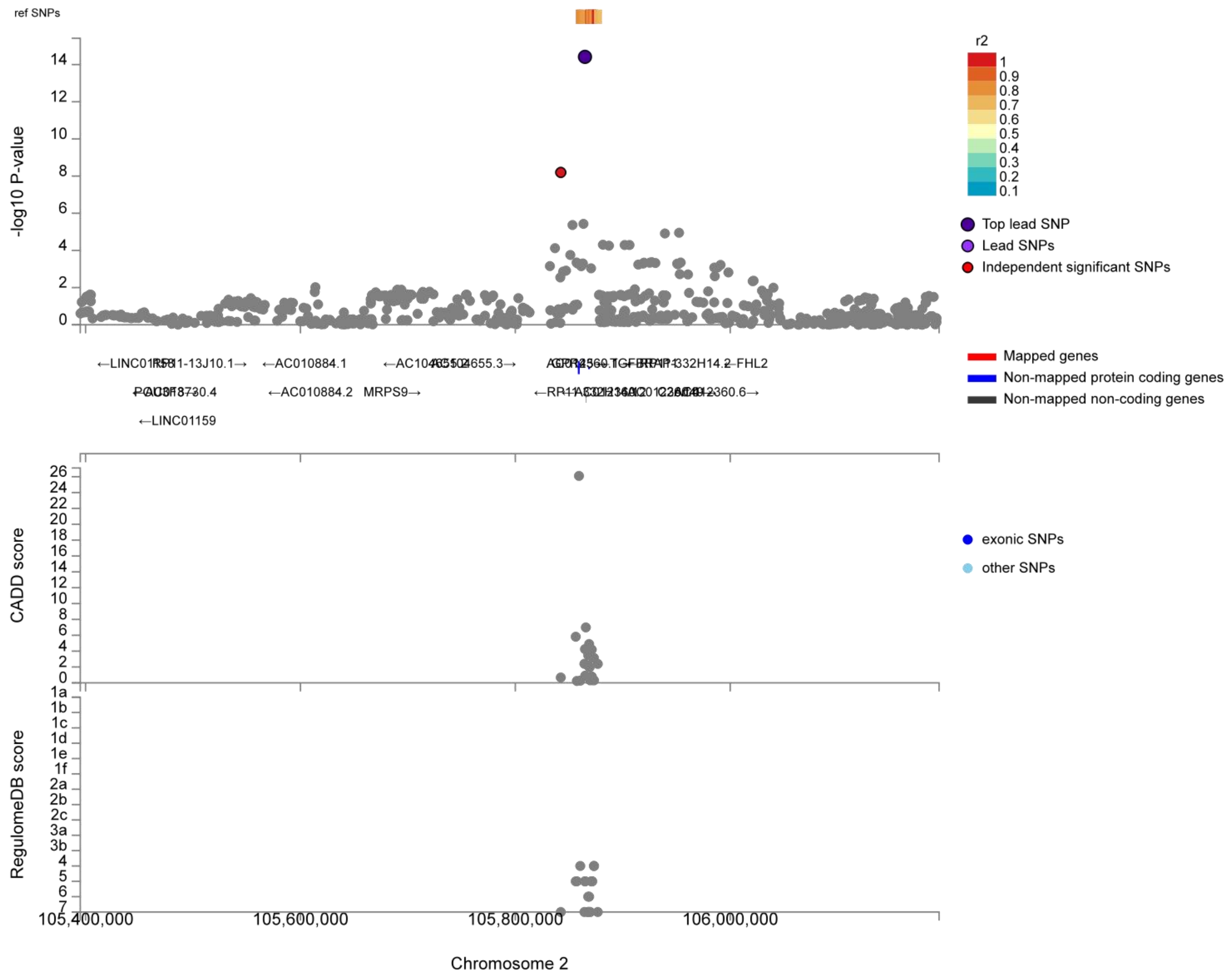

Supplementary Fig. 10. Regional association plot for SNP rs6758290.

Regional plot of the locus around the lead variant rs6758290 identified in the mvPuberty multivariate genome-wide association study (GWAS) (N=514,750). Top Top panel presents the -log10(P-values) results of two-sided Wald tests for each variant on mvPuberty and linkage disequilibrium (LD) R^2 information for the variants in the locus (variants are colored by LD R^2) and the genes prioritized by FUMA are highlighted in red on the track below.

Bottom The bottom panel presents the CADD (combined annotation dependent depletion) scores and RegulomeDB scores (top and bottom tracks, respectively).

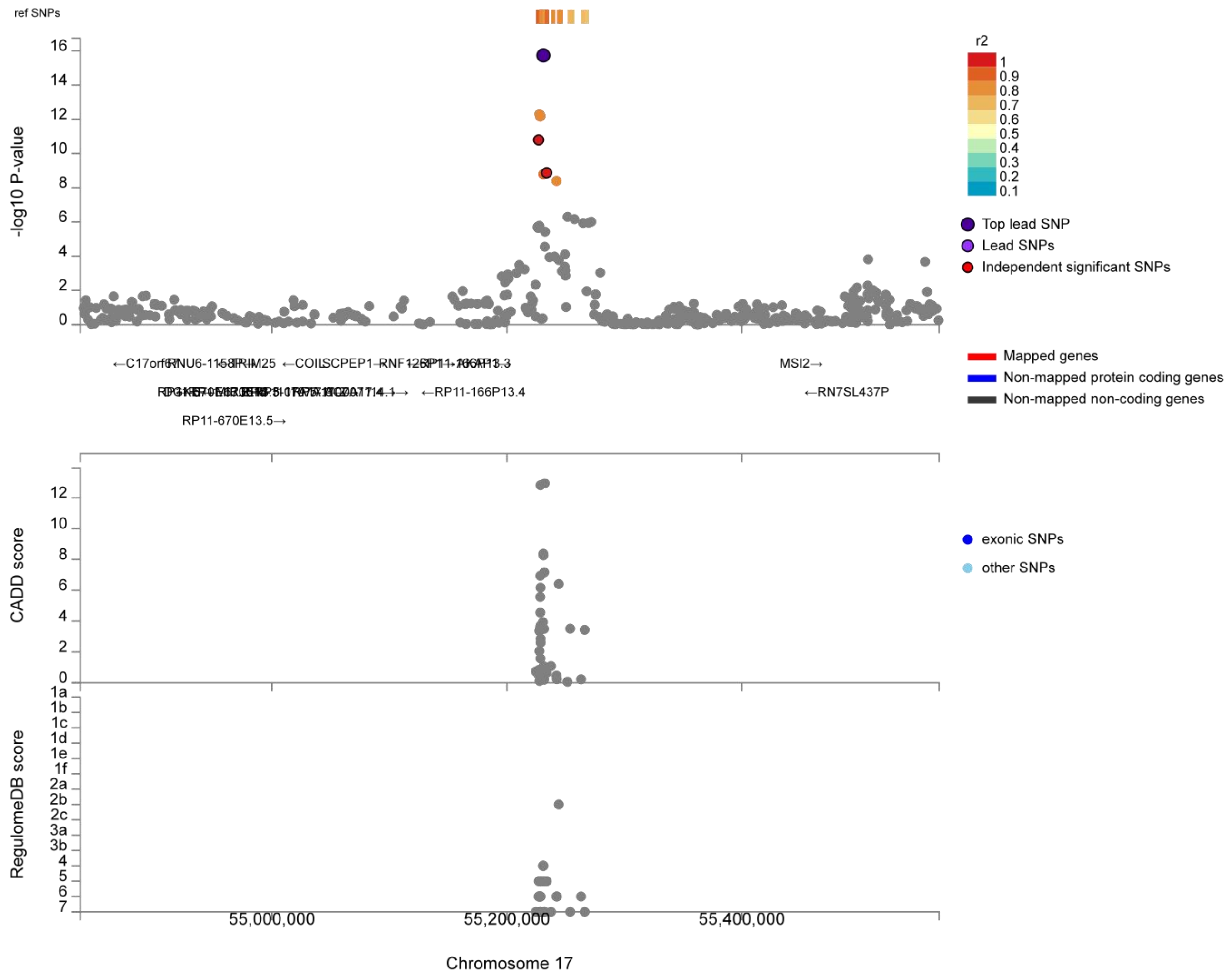

Supplementary Fig. 11. Regional association plot for SNP rs9907841.
Regional plot of the locus around the lead variant rs9907841 identified in the mvPuberty multivariate genome-wide association study (GWAS) (N=514,750). Top Top panel presents the -log10(P-values) results of two-sided Wald tests for each variant on mvPuberty and linkage disequilibrium (LD) R^2 information for the variants in the locus (variants are colored by LD R^2) and the genes prioritized by FUMA are highlighted in red on the track below.
Bottom The bottom panel presents the CADD (combined annotation dependent depletion) scores and RegulomeDB scores (top and bottom tracks, respectively).

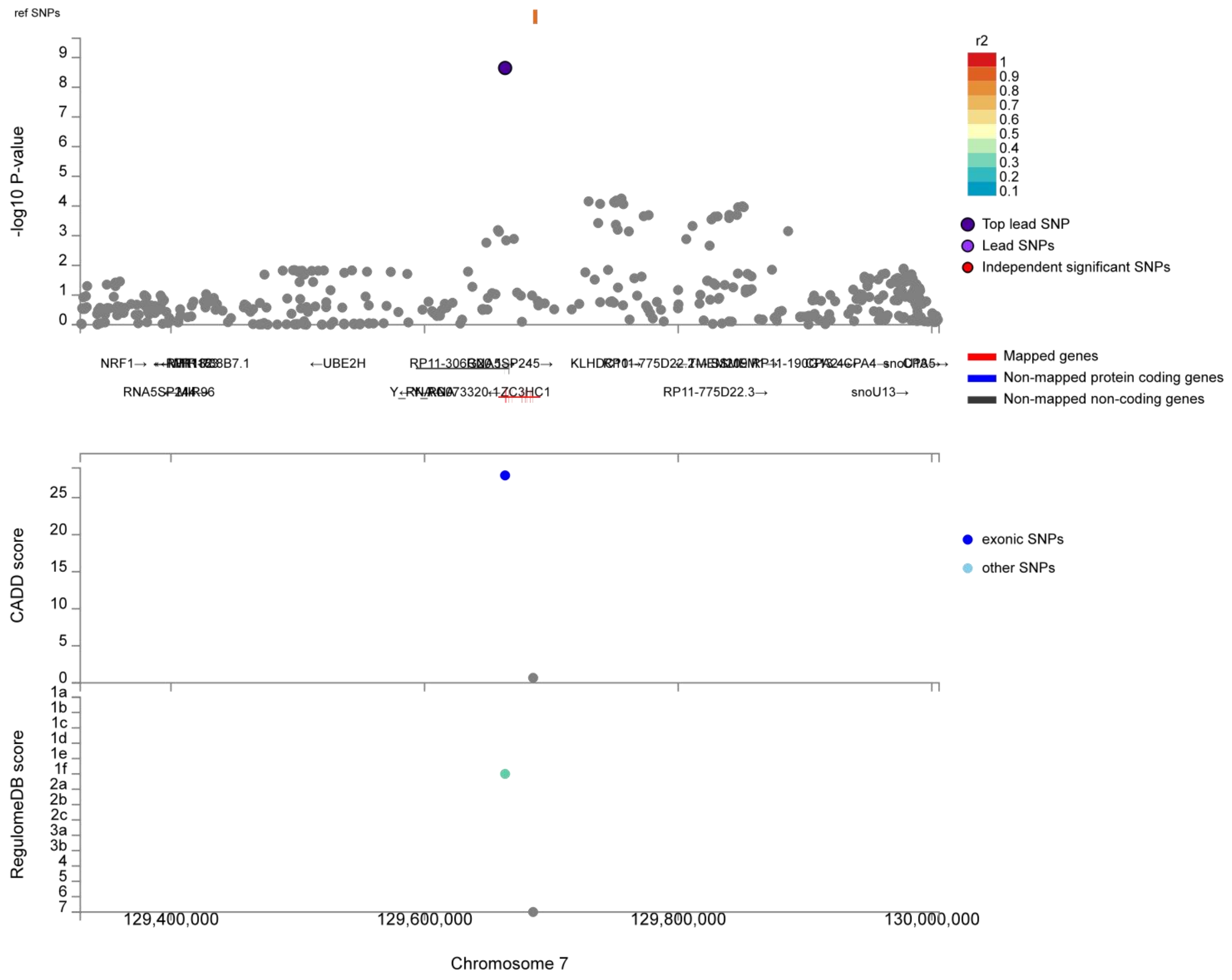

Supplementary Fig. 12. Regional association plot for SNP rs11556924.

Regional plot of the locus around the lead variant rs11556924 identified in the mvPuberty multivariate genome-wide association study (GWAS) (N=514,750). Top Top panel presents the -log10(P-values) results of two-sided Wald tests for each variant on mvPuberty and linkage disequilibrium (LD) R^2 information for the variants in the locus (variants are colored by LD R^2) and the genes prioritized by FUMA are highlighted in red on the track below.

Bottom The bottom panel presents the CADD (combined annotation dependent depletion) scores and RegulomeDB scores (top and bottom tracks, respectively).

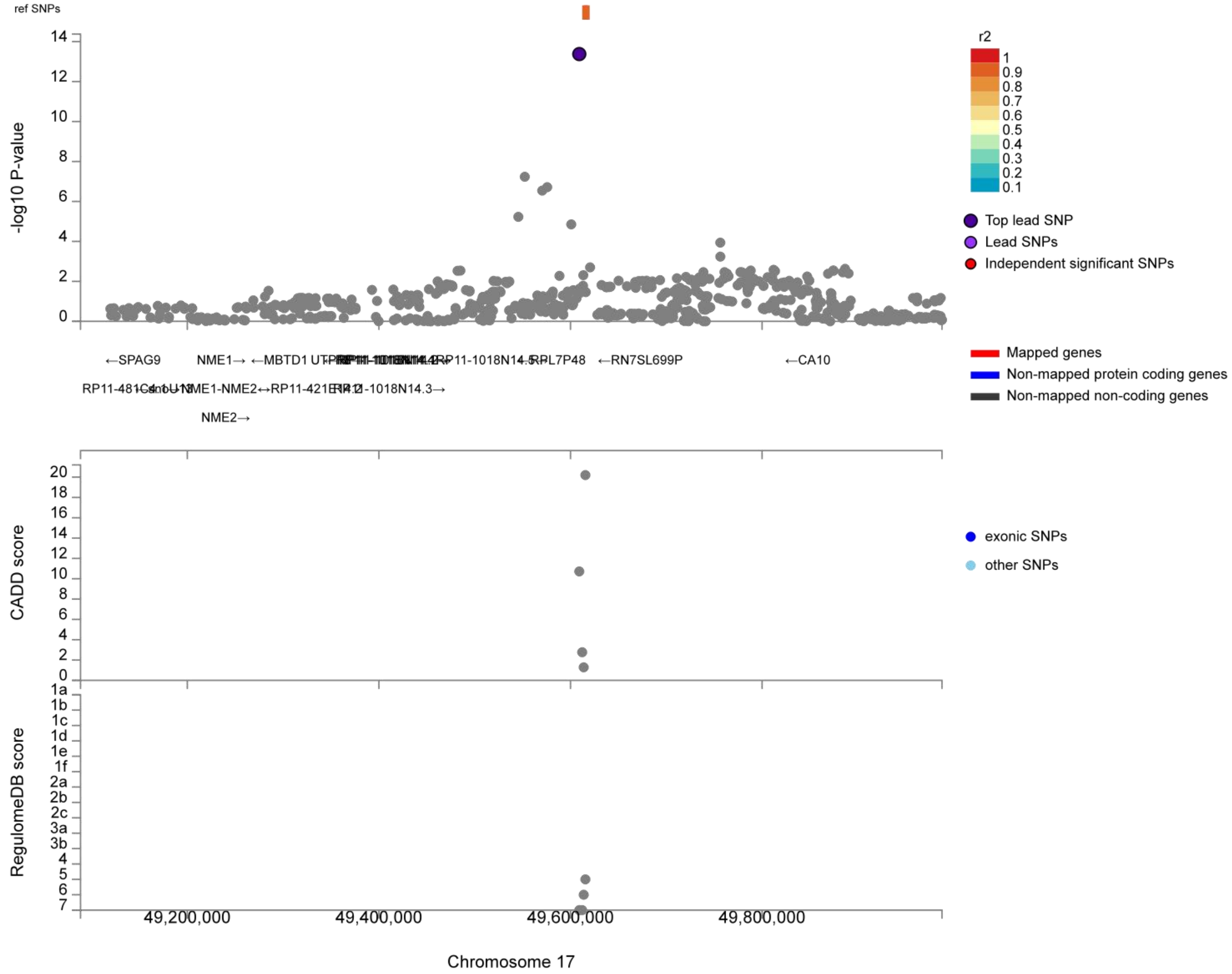

Supplementary Fig. 13. Regional association plot for SNP rs11853016.

Regional plot of the locus around the lead variant rs11853016 identified in the mvPuberty multivariate genome-wide association study (GWAS) (N=514,750). Top Top panel presents the -log10(P-values) results of two-sided Wald tests for each variant on mvPuberty and linkage disequilibrium (LD) R^2 information for the variants in the locus (variants are colored by LD R^2) and the genes prioritized by FUMA are highlighted in red on the track below.

Bottom The bottom panel presents the CADD (combined annotation dependent depletion) scores and RegulomeDB scores (top and bottom tracks, respectively).

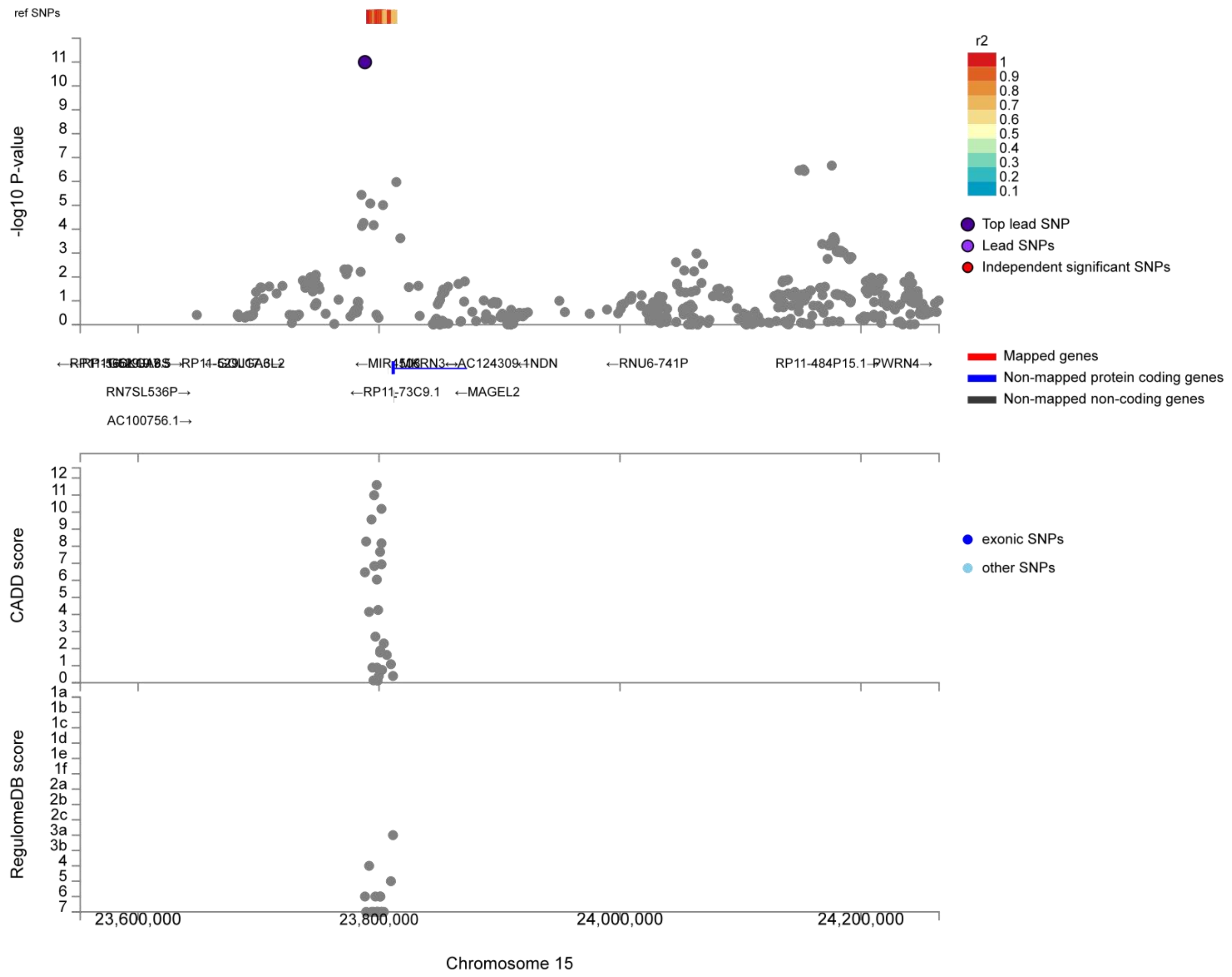

Supplementary Fig. 14. Regional association plot for SNP rs11867780.

Regional plot of the locus around the lead variant rs11867780 identified in the mvPuberty multivariate genome-wide association study (GWAS) (N=514,750). Top Top panel presents the -log10(P-values) results of two-sided Wald tests for each variant on mvPuberty and linkage disequilibrium (LD) R^2 information for the variants in the locus (variants are colored by LD R^2) and the genes prioritized by FUMA are highlighted in red on the track below.

Bottom The bottom panel presents the CADD (combined annotation dependent depletion) scores and RegulomeDB scores (top and bottom tracks, respectively).

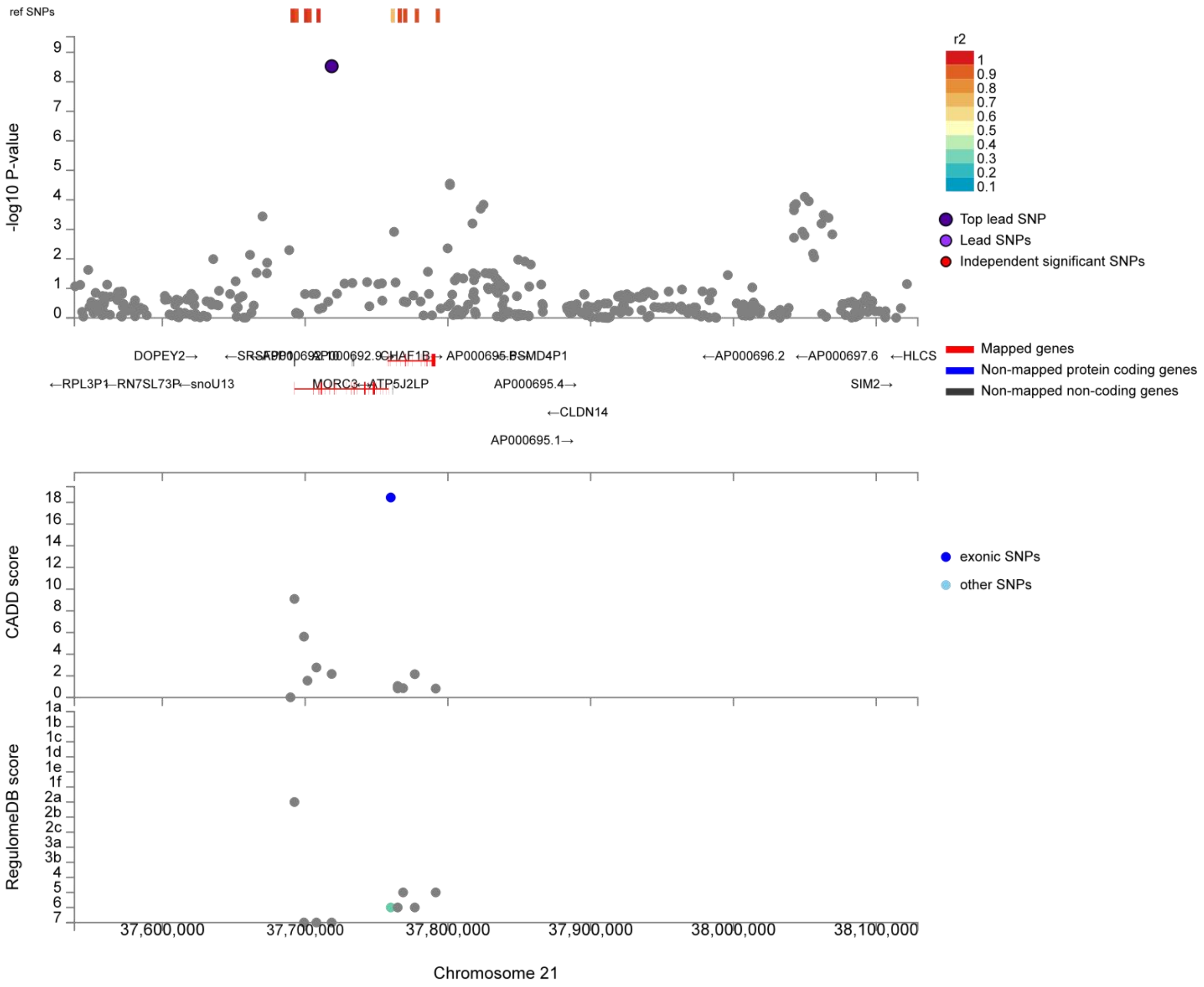

Supplementary Fig. 15. Regional association plot for SNP rs12482030.

Regional plot of the locus around the lead variant rs12482030 identified in the mvPuberty multivariate genome-wide association study (GWAS) (N=514,750). Top Top panel presents the -log10(P-values) results of two-sided Wald tests for each variant on mvPuberty and linkage disequilibrium (LD) R^2 information for the variants in the locus (variants are colored by LD R^2) and the genes prioritized by FUMA are highlighted in red on the track below.

Bottom The bottom panel presents the CADD (combined annotation dependent depletion) scores and RegulomeDB scores (top and bottom tracks, respectively).

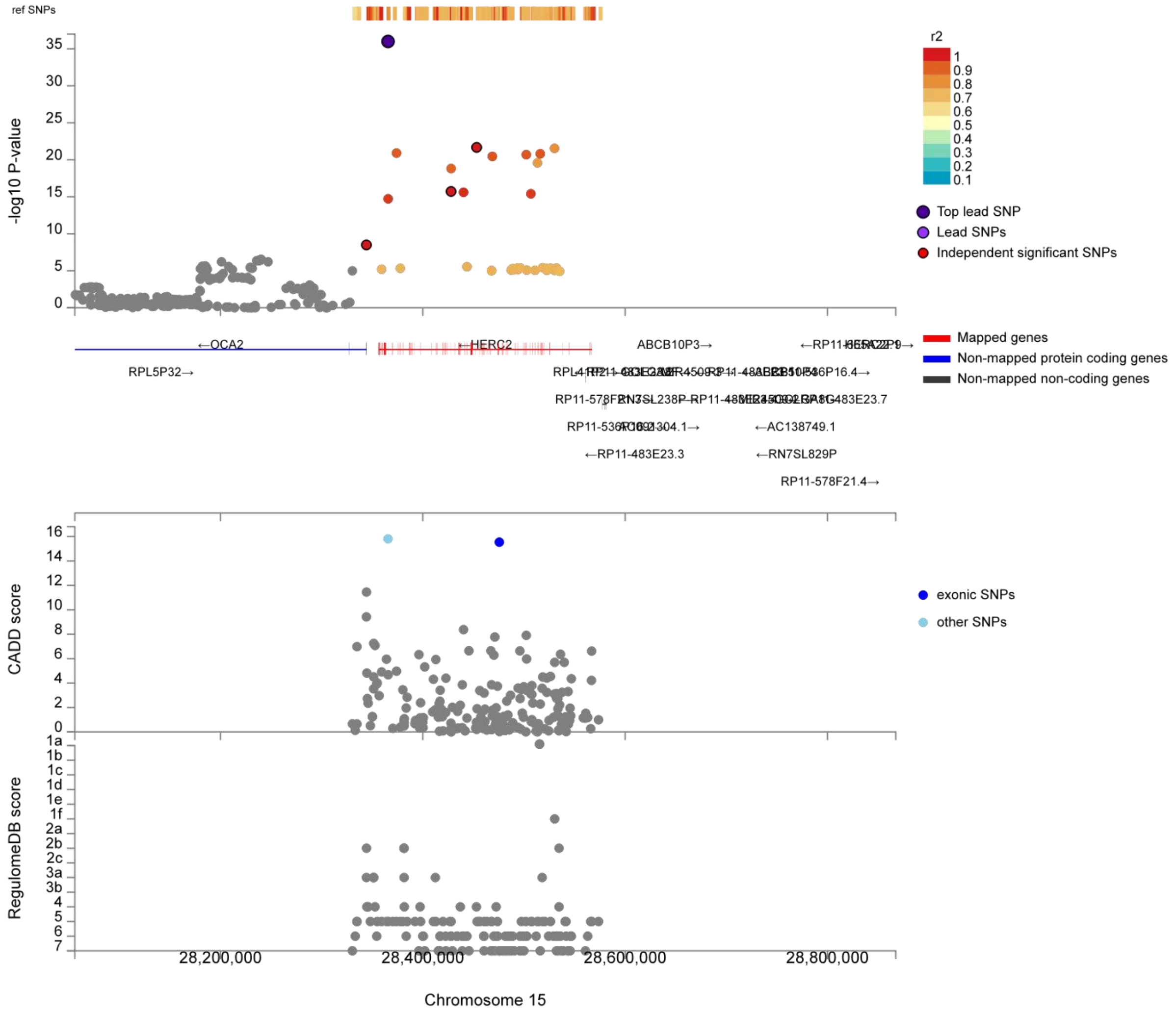

Supplementary Fig. 16. Regional association plot for SNP rs12913832.

Regional plot of the locus around the lead variant rs12913832 identified in the mvPuberty multivariate genome-wide association study (GWAS) (N=514,750). Top Top panel presents the -log10(P-values) results of two-sided Wald tests for each variant on mvPuberty and linkage disequilibrium (LD) R^2 information for the variants in the locus (variants are colored by LD R^2) and the genes prioritized by FUMA are highlighted in red on the track below.

Bottom The bottom panel presents the CADD (combined annotation dependent depletion) scores and RegulomeDB scores (top and bottom tracks, respectively).

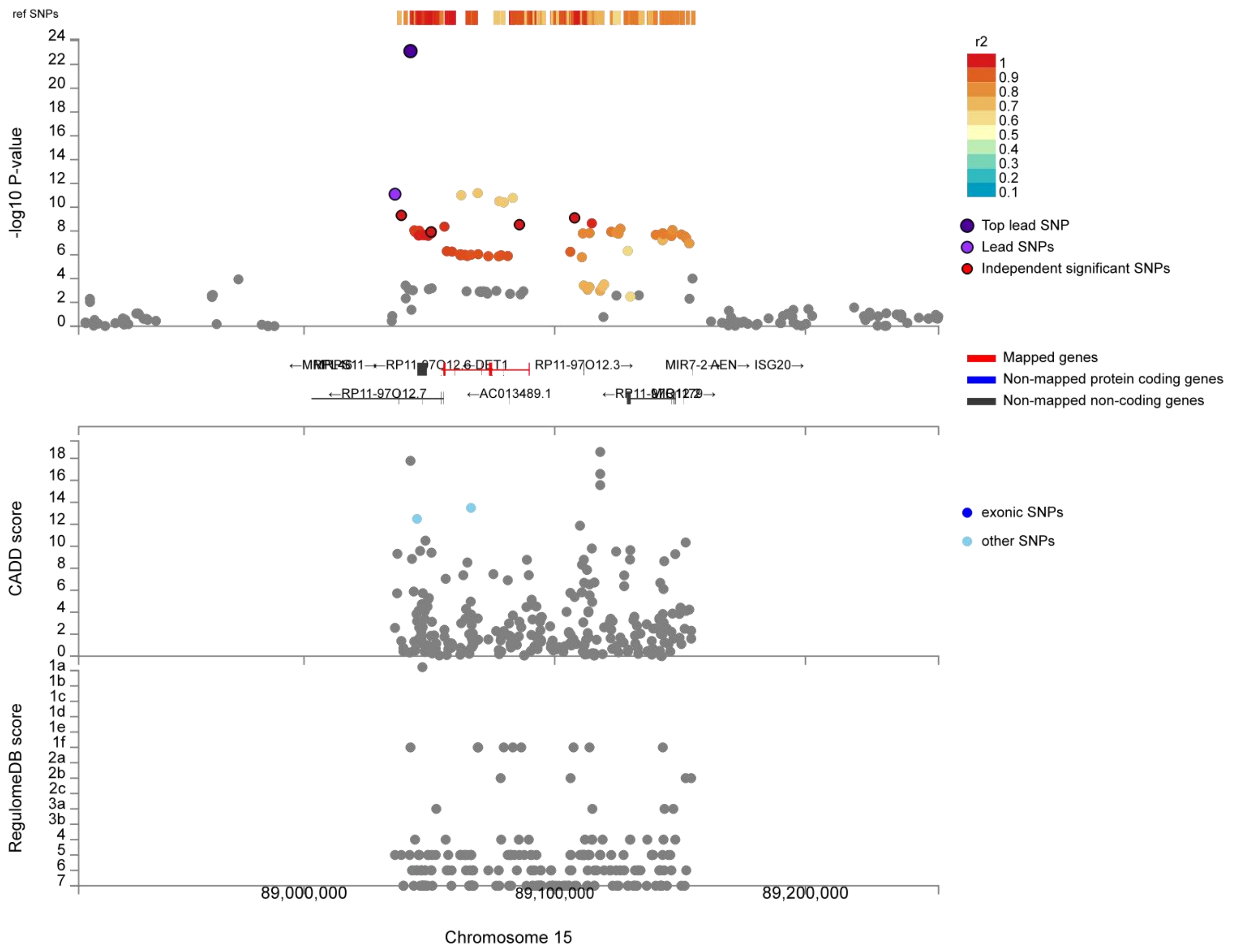

Supplementary Fig. 17. Regional association plot for SNP rs12915845.

Regional plot of the locus around the lead variant rs12915845 identified in the mvPuberty multivariate genome-wide association study (GWAS) (N=514,750). Top Top panel presents the -log10(P-values) results of two-sided Wald tests for each variant on mvPuberty and linkage disequilibrium (LD) R^2 information for the variants in the locus (variants are colored by LD R^2) and the genes prioritized by FUMA are highlighted in red on the track below.

Bottom The bottom panel presents the CADD (combined annotation dependent depletion) scores and RegulomeDB scores (top and bottom tracks, respectively).

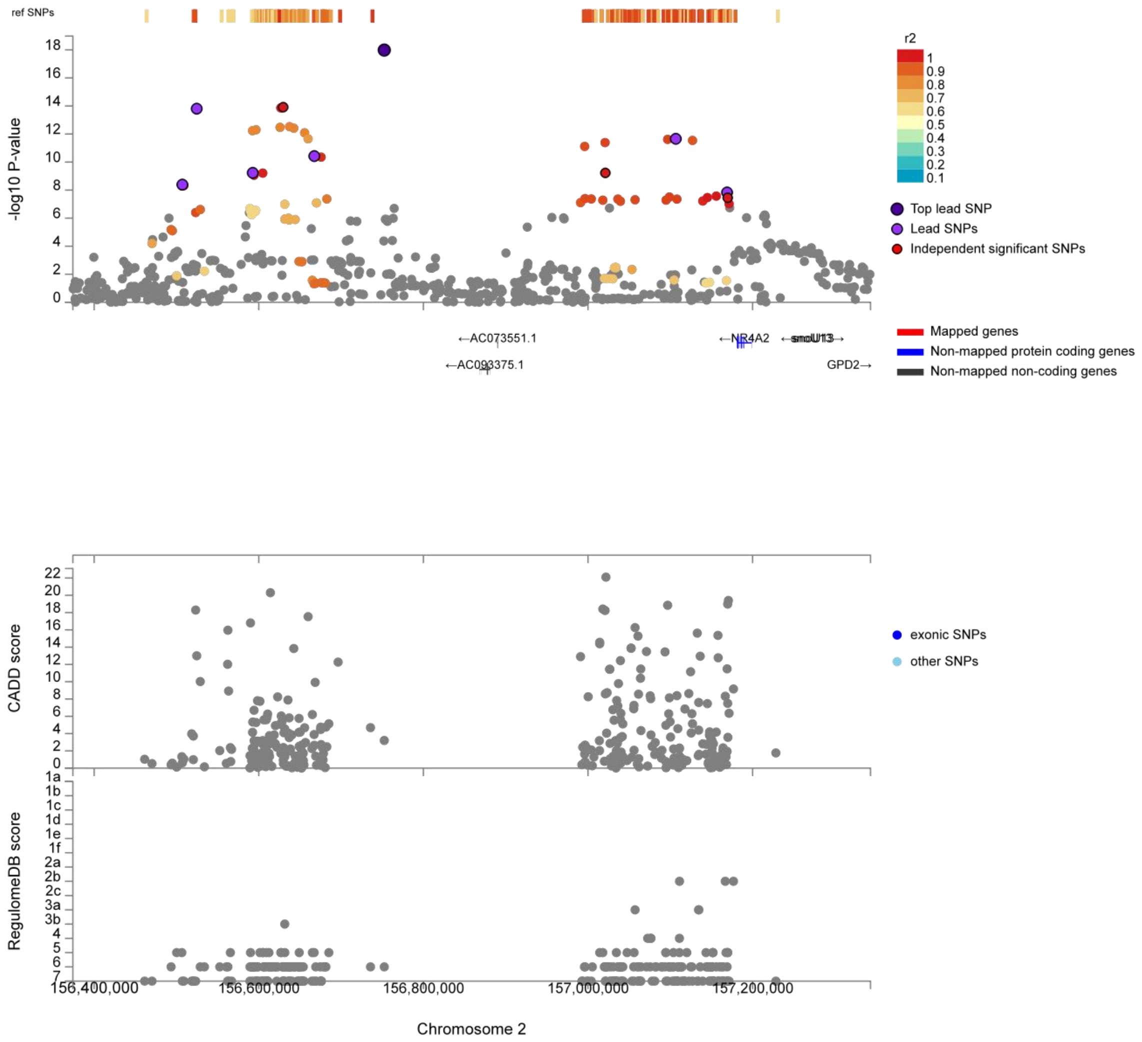

Supplementary Fig. 18. Regional association plot for SNP rs17236969.
Regional plot of the locus around the lead variant rs17236969 identified in the mvPuberty multivariate genome-wide association study (GWAS) (N=514,750). Top Top panel presents the -log10(P-values) results of two-sided Wald tests for each variant on mvPuberty and linkage disequilibrium (LD) R^2 information for the variants in the locus (variants are colored by LD R^2) and the genes prioritized by FUMA are highlighted in red on the track below.
Bottom The bottom panel presents the CADD (combined annotation dependent depletion) scores and RegulomeDB scores (top and bottom tracks, respectively).

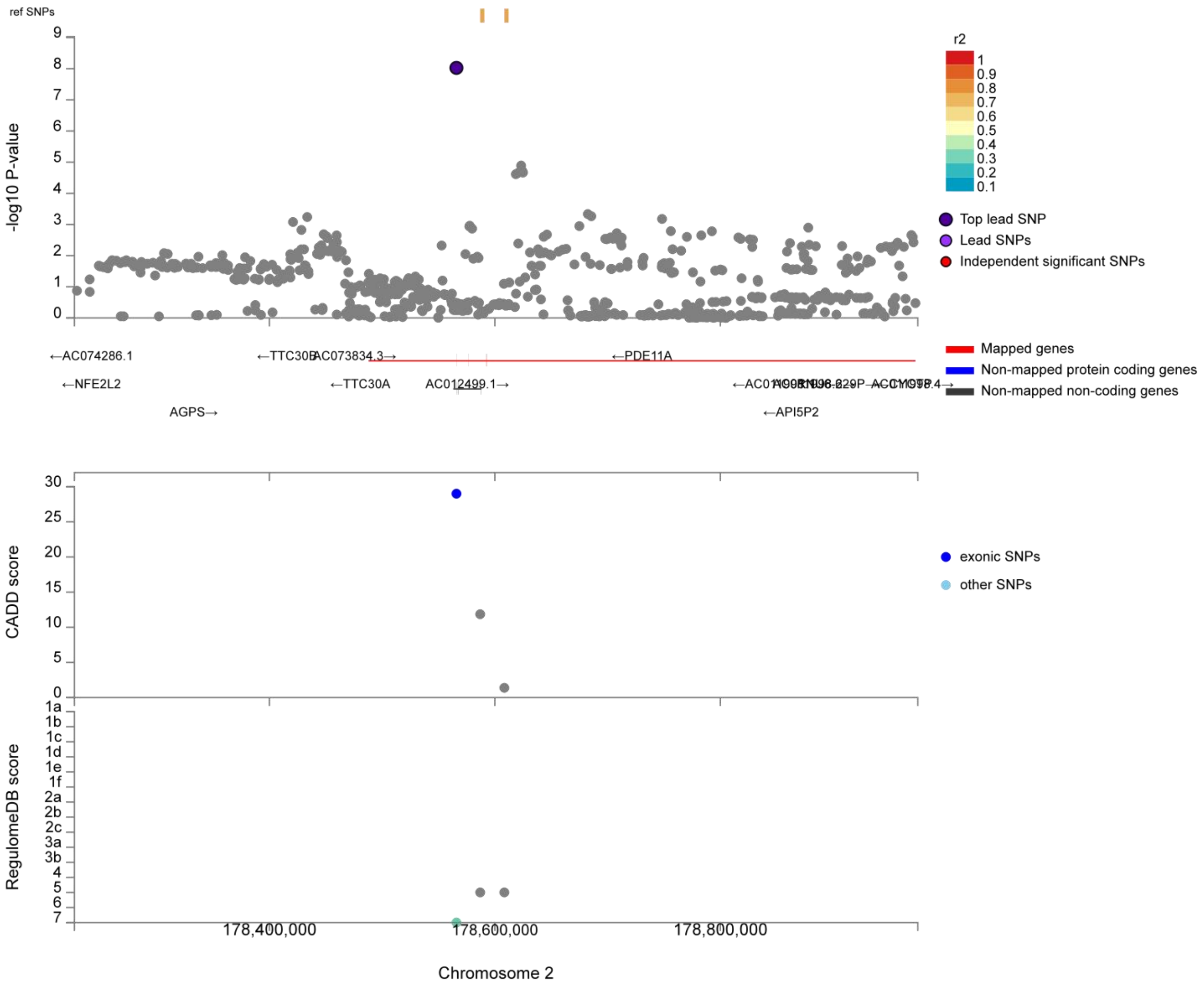

Supplementary Fig. 19. Regional association plot for SNP rs17400325.
Regional plot of the locus around the lead variant rs17400325 identified in the mvPuberty multivariate genome-wide association study (GWAS) (N=514,750). Top Top panel presents the -log10(P-values) results of two-sided Wald tests for each variant on mvPuberty and linkage disequilibrium (LD) R^2 information for the variants in the locus (variants are colored by LD R^2) and the genes prioritized by FUMA are highlighted in red on the track below.
Bottom The bottom panel presents the CADD (combined annotation dependent depletion) scores and RegulomeDB scores (top and bottom tracks, respectively).

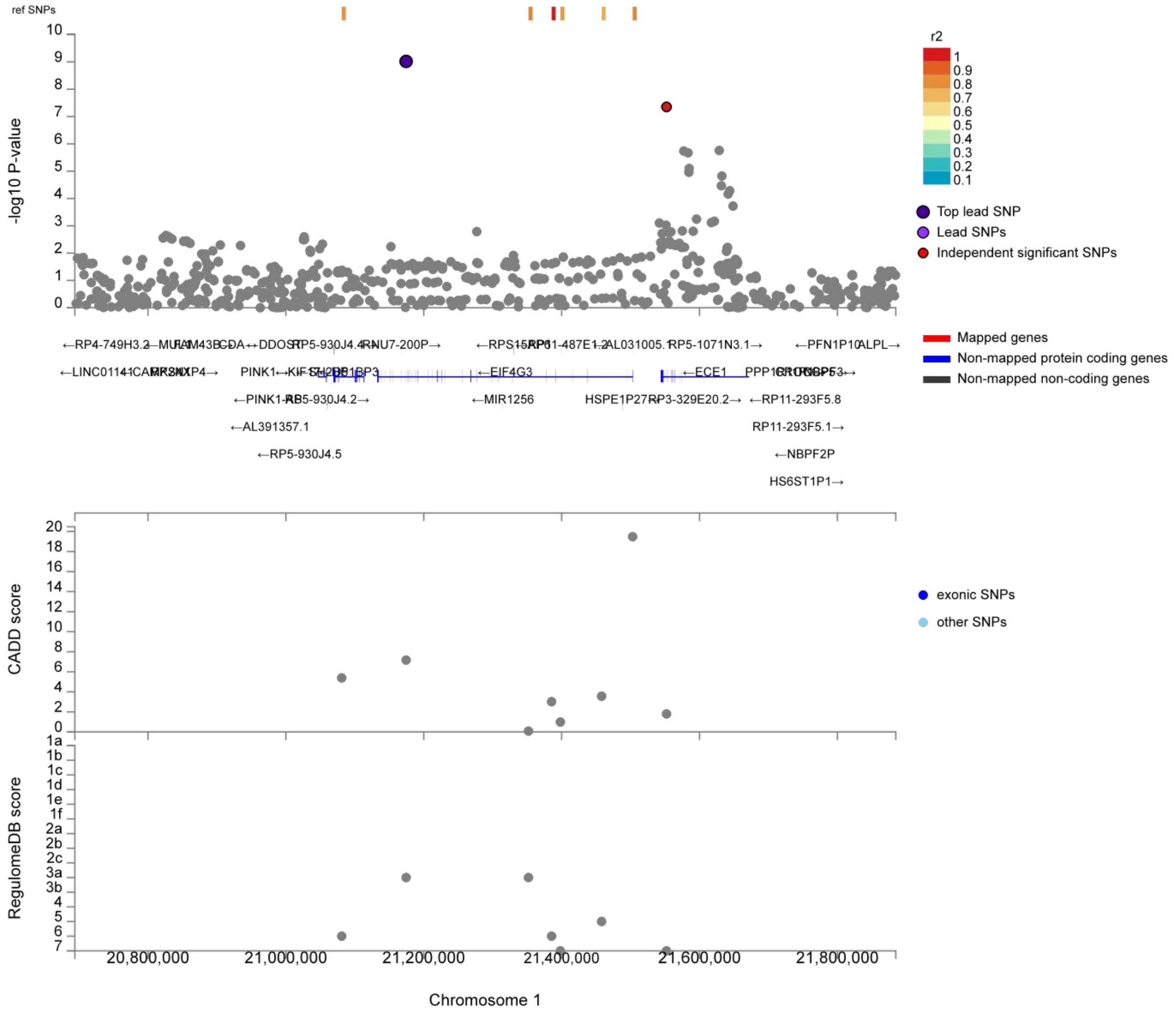

Supplementary Fig. 20. Regional association plot for SNP rs17449243.

Regional plot of the locus around the lead variant rs17449243 identified in the mvPuberty multivariate genome-wide association study (GWAS) (N=514,750). Top Top panel presents the -log10(P-values) results of two-sided Wald tests for each variant on mvPuberty and linkage disequilibrium (LD) R^2 information for the variants in the locus (variants are colored by LD R^2) and the genes prioritized by FUMA are highlighted in red on the track below.

Bottom The bottom panel presents the CADD (combined annotation dependent depletion) scores and RegulomeDB scores (top and bottom tracks, respectively).

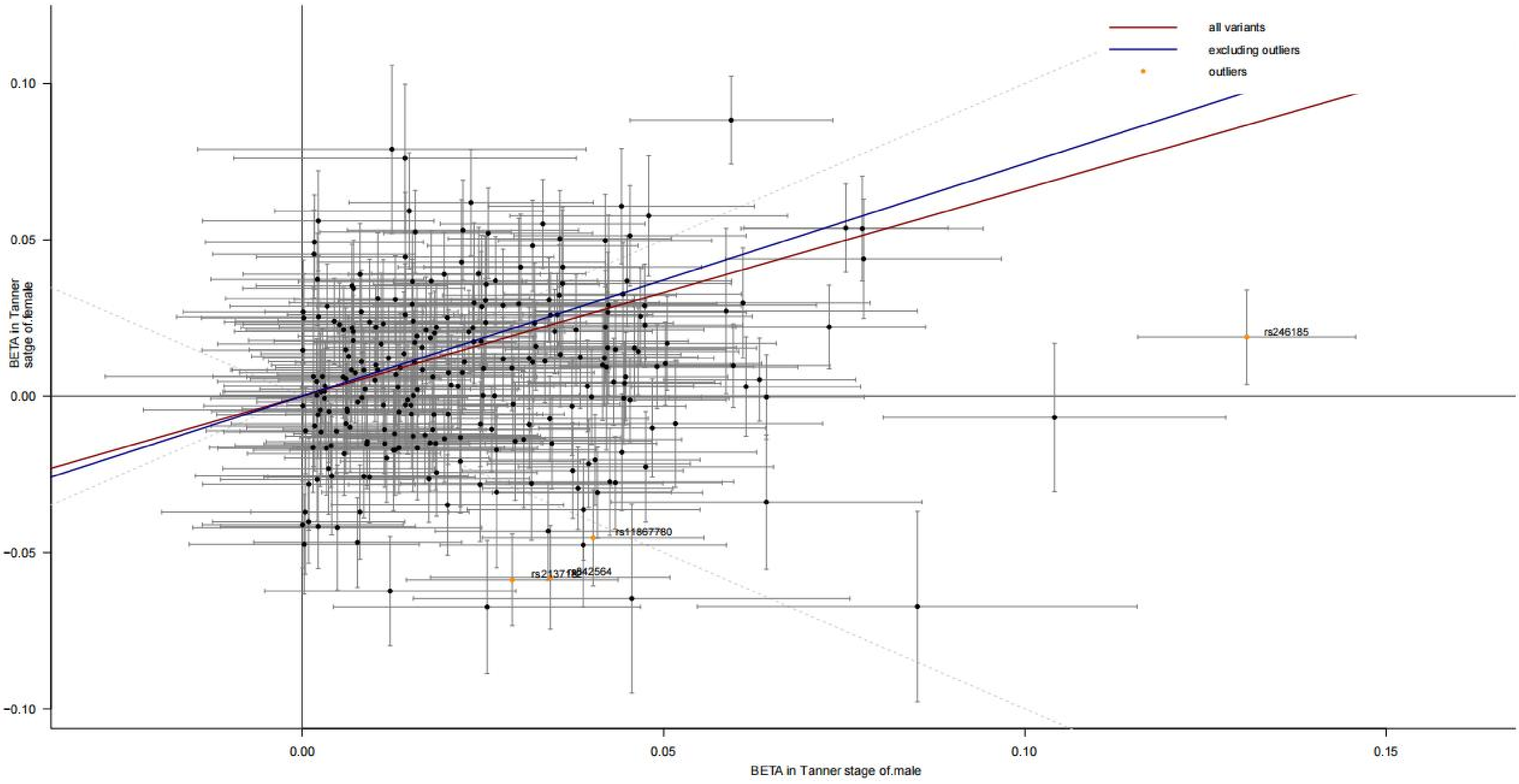

Supplementary Fig. 21. Comparison of effects of mvPuberty-associated loci in Tanner stage between males between females.
The 266 mvPuberty-associated loci were evaluated for whether their log odds ratio in Tanner stage was different in males versus females by comparing a GWAS beta of Tanner stage in males in ECG (x-axis; 3,769 participants) against the GWAS beta for Tanner stage in females (y-axis; 6,147 participants). Error bars indicate +/- 1 standard error in each GWAS. Points labelled with gene names are identified as outliers by SCOUTJOY. Fitted slopes are shown from York regression with all variants (blank) and after removal of identified outliers (orange). Dashed gray reference lines indicate equal absolute effect size. Data available in Supplementary Table 11-12.

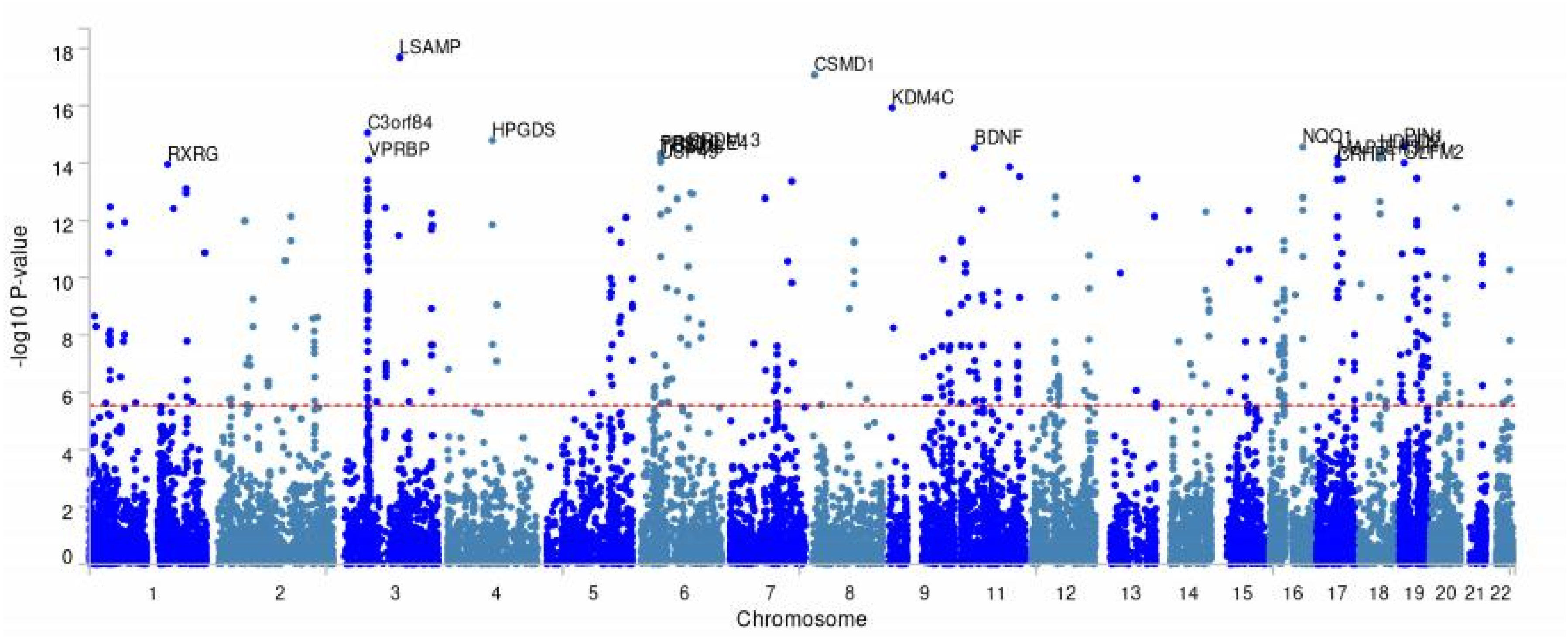

Supplementary Fig. 22. Manhattan plot of the gene-based test as computed by MAGMA based on mvPuberty summary statistic. Gene-based analyses MAGMA gene-based analyses included mapping SNPs to 18,649 protein coding genes within 10 kilobases of lead SNPs using several regression methods to account for LD between SNPs (using the1000 Genomes European (EUR) sample as the reference). Data available in Supplementary Data 14.

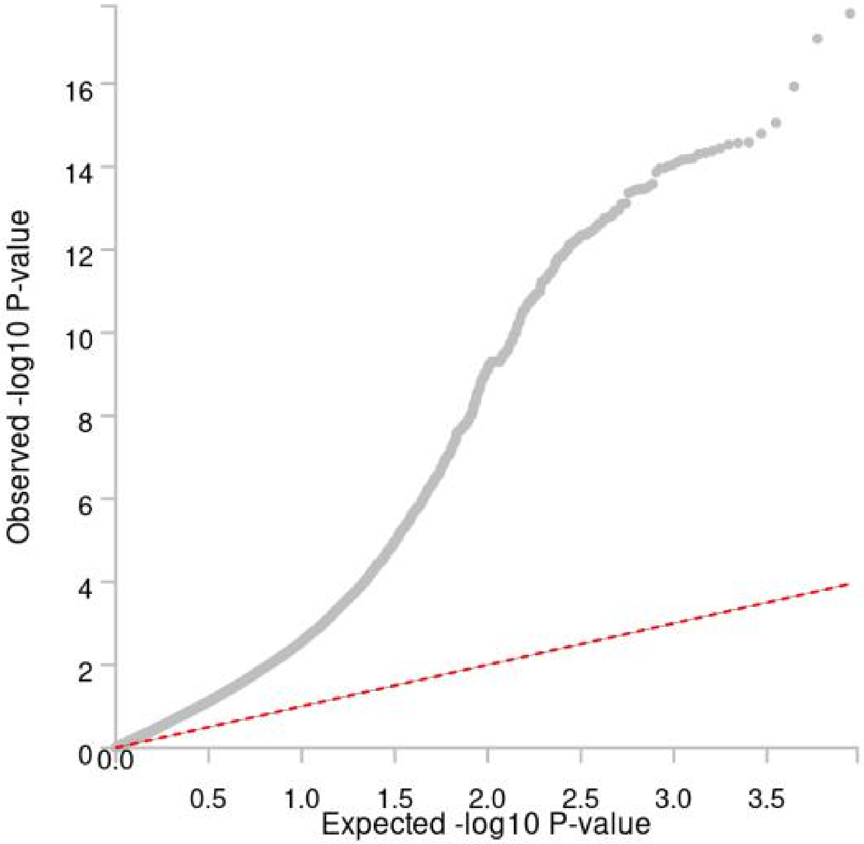

Supplementary Fig. 23. This is a Q-Q plot of the gene-based test computed by MAGMA using mvPuberty summary statistic.
Plot represents a comparison of the mvPuberty multivariate genome-wide association study (GWAS) (N=514,750); P values to those expected for null distribution (depicted by the red line).

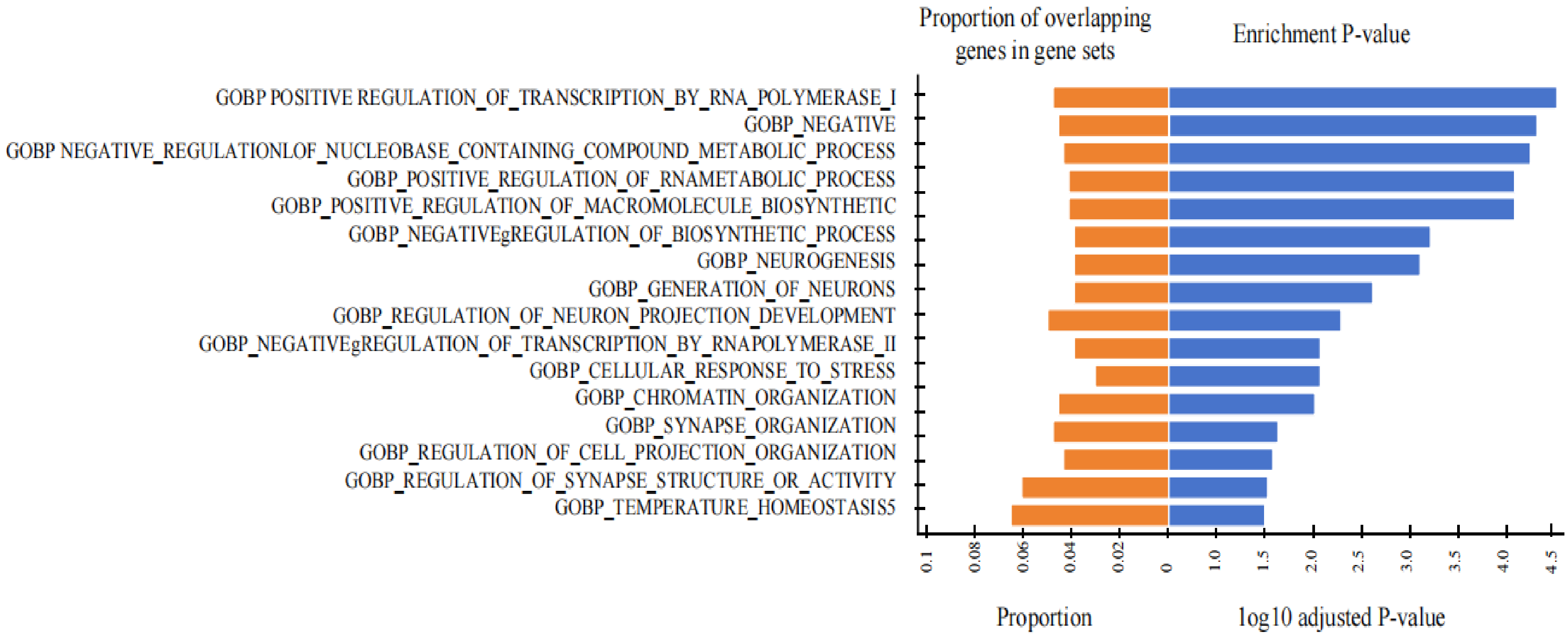

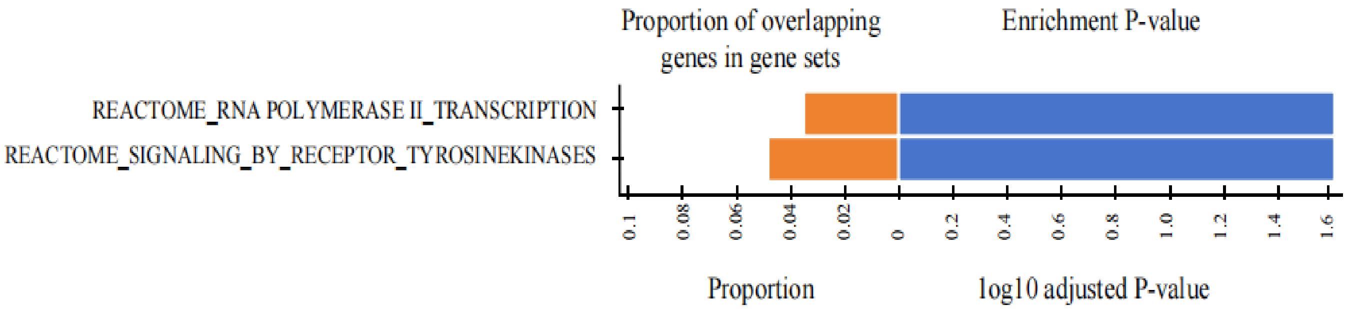

Supplementary Fig. 24. Heat map of Gene-set enrichment analysis for GO_BP and Reactome terms obtained from MsigDB. Data available in Supplementary Data 15.

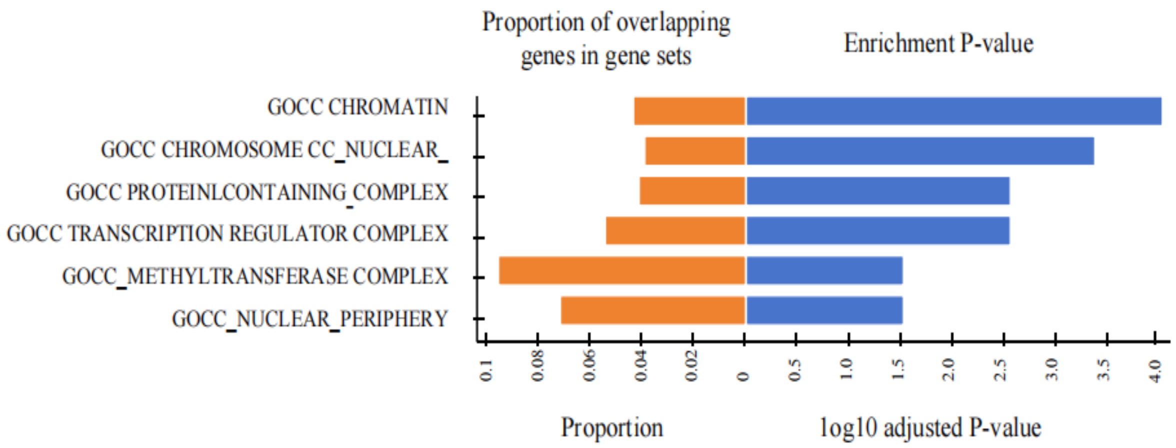

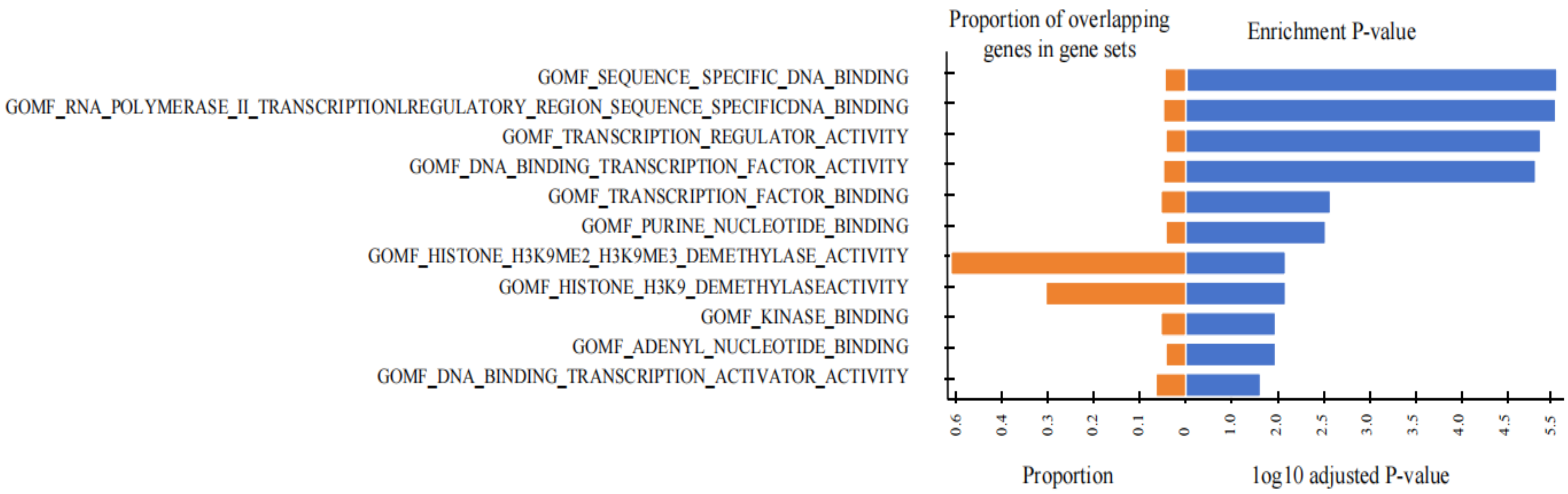

Supplementary Fig. 25. Heat map of Gene-set enrichment analysis for GO_CC and MF terms obtained from MsigDB. Data available in Supplementary Data 15.

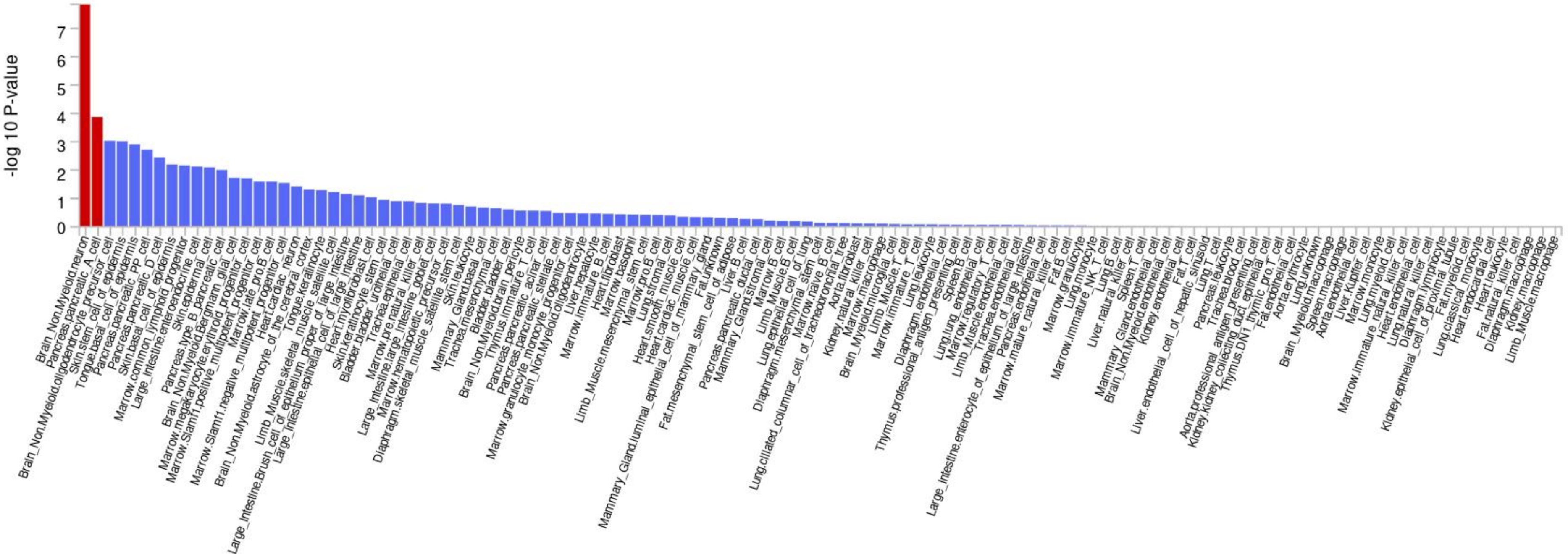

Supplementary Fig. 26. Cene property analysis of cell specific expression.
Unadjusted one-sided p-values from the FUMA gene-property analysis v1.60 linear regression for association of MAGMA gene-level results for mvPuberty on cell specific enrichment using Tabula Muris scRNA-seq data.

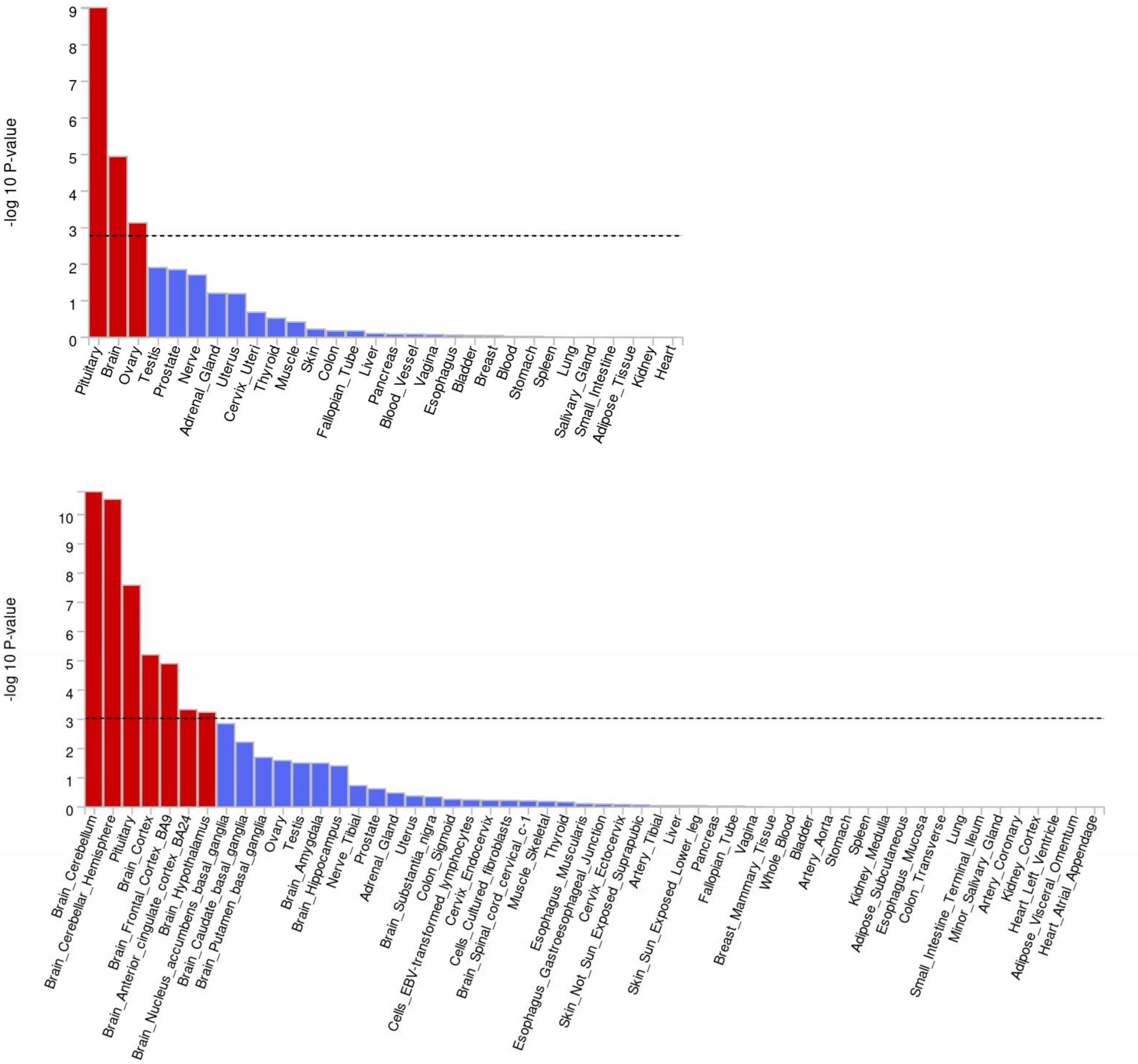

Supplementary Figure 27: Gene property analysis of tissue specific expression.
Unadjusted one-sided p-values from the FUMA gene-property analysis v1.60 linear regression for association of MAGMA gene-level results for mvPuberty on tissue specific gene expression across 17310 genes. Performed using FUMA SNP2GENE based on tissue specificity analysis of expression in GTEx.

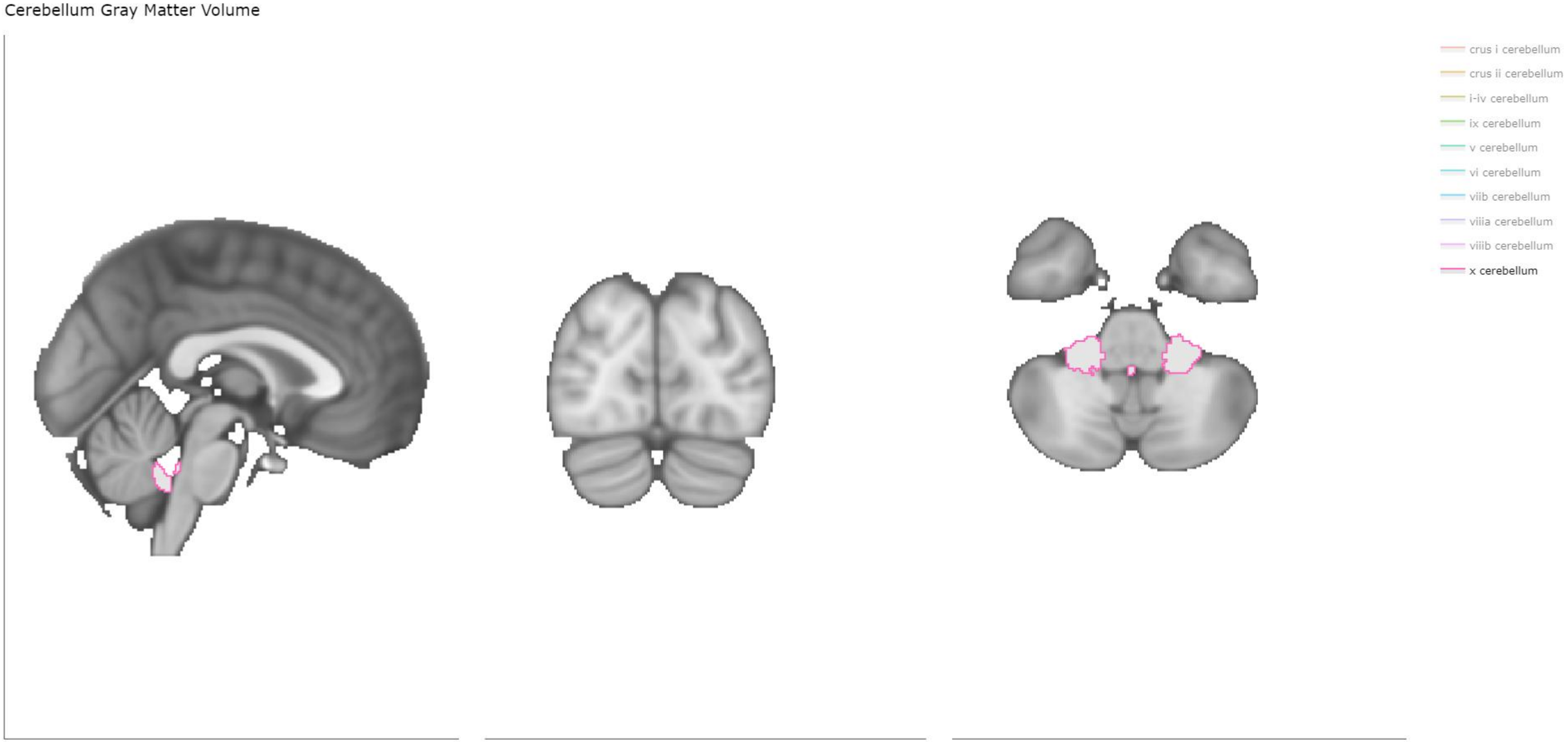

Supplementary Figure 28: Brain T1-Cerebellum regions visualization of structural features associations with mvPuberty risk. Data available in Supplementary Data 20. Color means higher correlation.

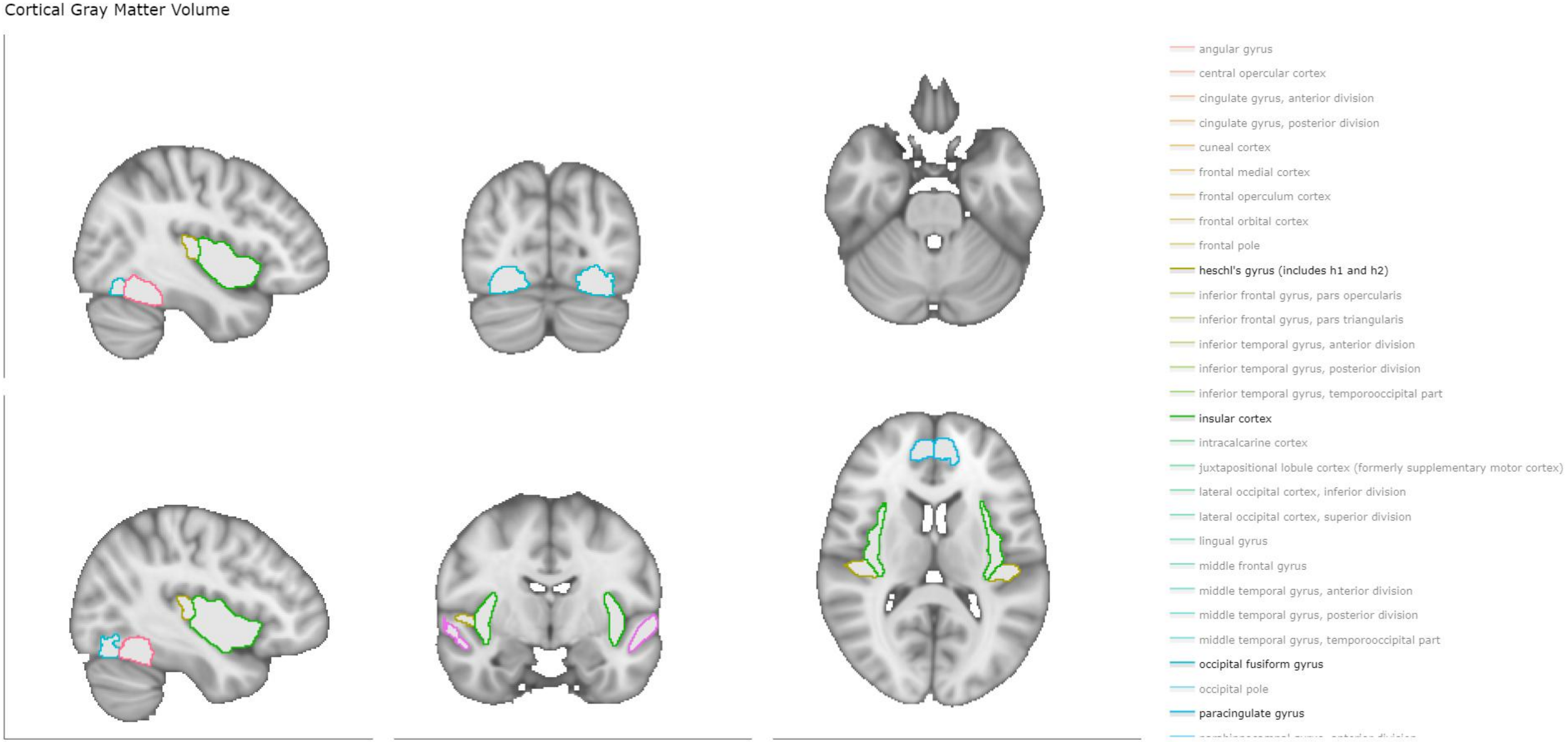

Supplementary Figure 29: Brain T1-Cortical regions visualization of structural features associations with mvPuberty risk. Data available in Supplementary Data 20. Color means higher correlation

Diffusion MRI

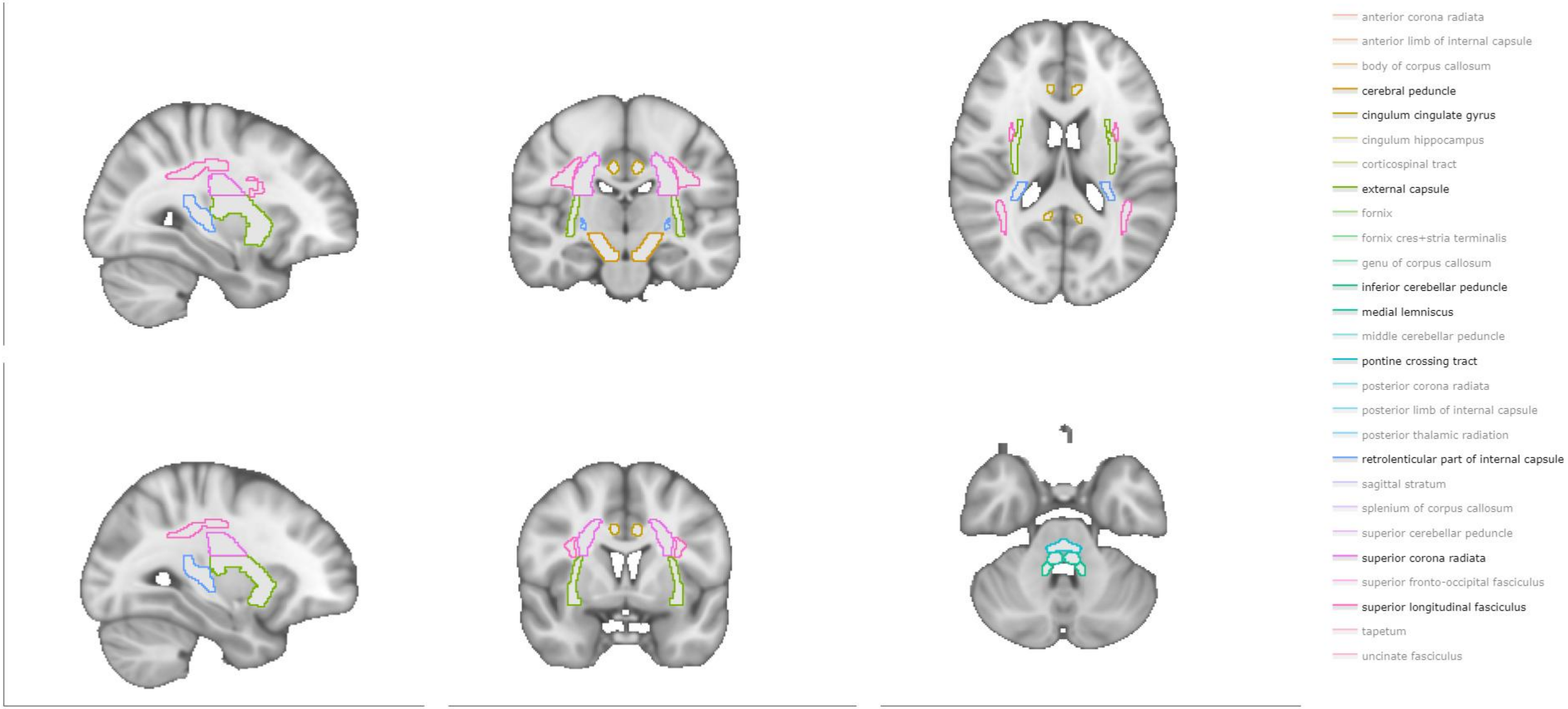

Supplementary Figure 30: Brain MRI-IVCF regions visualization of structural features associations with mvPuberty risk. Data available in Supplementary Data 21. Color means higher correlation

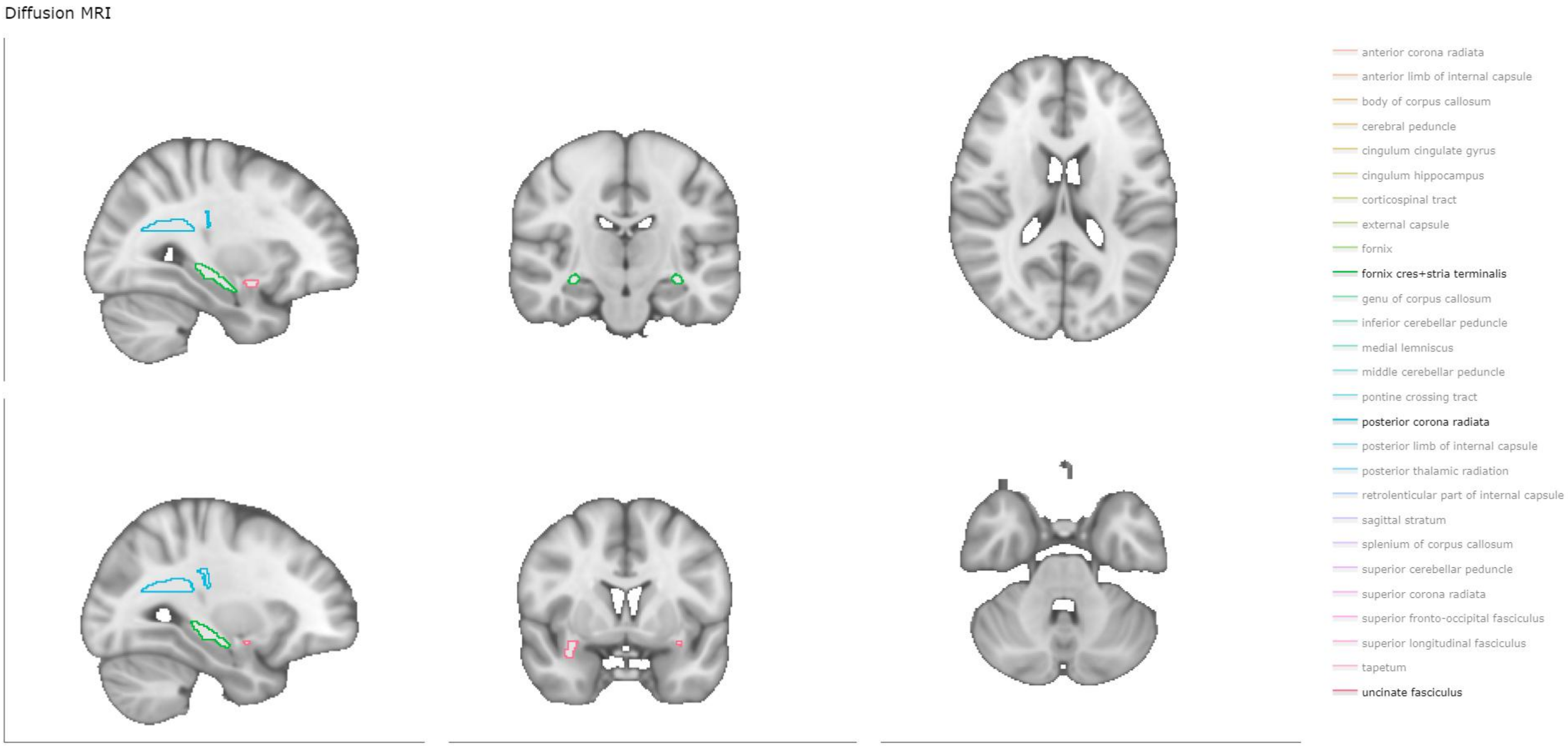

Supplementary Figure 31: Brain MRI-FA regions visualization of structural features associations with mvPuberty risk. Data available in Supplementary Data 20. Color means higher correlation

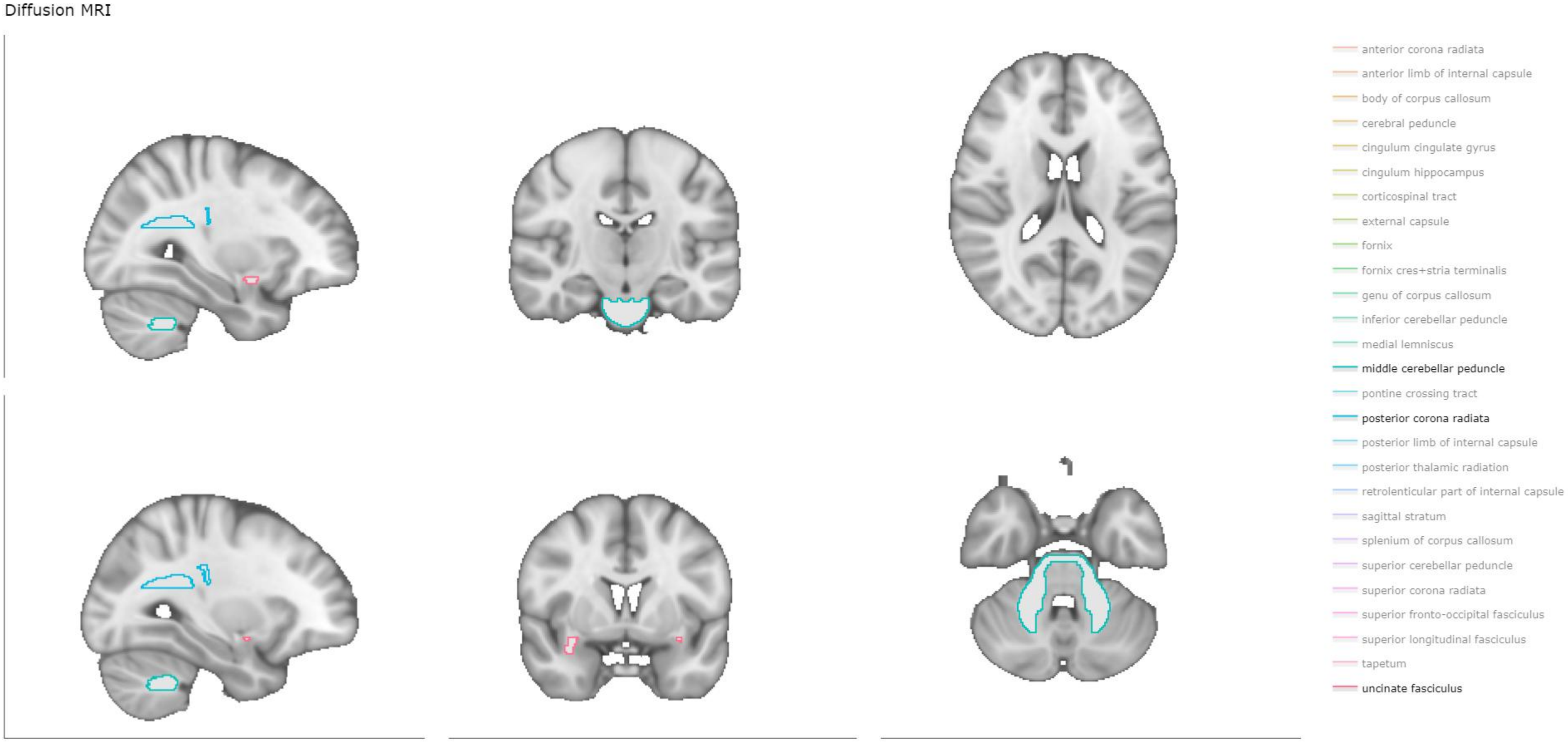

Supplementary Figure 32: Brain MRI-OD regions visualization of structural features associations with mvPuberty risk. Data available in Supplementary Data 20. Color means higher correlation

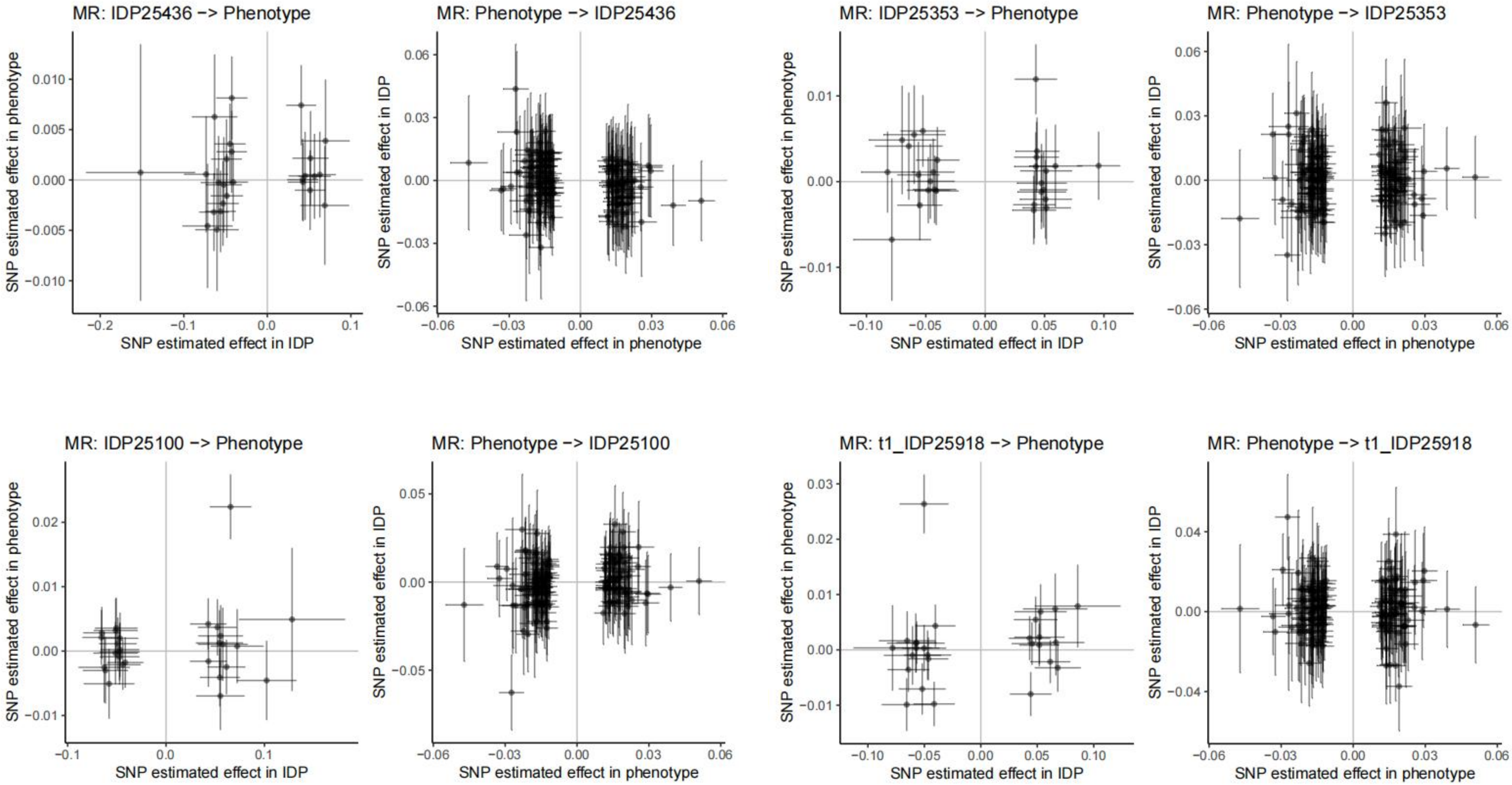

Supplementary Figure 33: Significant MR results between mvPuberty and brain features using BrainXcan. Data available in Supplementary Data 21.

Type of Outcome	Outcome	Methods	β	P	Q	Q_pval	correct_causal_direction
Cardiovascular disease							
	Coronary_artery_disease	Inverse variance weighted	-0.21	1.40×10^{-4}	304.93	1.25×10^{-14}	TRUE
		MRlap	-0.10	3.37×10^{-5}	–	–	–
		MR LASSO	-0.26	8.99×10^{-12}	–	–	–
	Heart_failure	Inverse variance weighted	-0.30	1.88×10^{-7}	247.30	3.25×10^{-8}	TRUE
		MRlap	-0.07	3.63×10^{-8}	–	–	–
		MR LASSO	-0.24	6.65×10^{-9}	–	–	–
Aging							
	mvAge	Inverse variance weighted	0.04	7.45×10^{-6}	410.85	7.11×10^{-30}	TRUE
		MRlap	0.05	2.37×10^{-5}	–	–	–
		MR LASSO	0.04	1.75×10^{-8}	–	–	–
	Parental_lifespan	Inverse variance weighted	0.14	2.43×10^{-5}	318.16	5.16×10^{-17}	TRUE
		MRlap	0.06	1.75×10^{-4}	–	–	–
		MR LASSO	0.11	8.15×10^{-7}	–	–	–
Cancer							
	Endometrial cancer	Inverse variance weighted	-0.54	1.12×10^{-9}	159.70	9.96×10^{-2}	TRUE
		MRlap	-0.15	1.44×10^{-7}	–	–	–
		MR LASSO	-0.44	2.20×10^{-8}	–	–	–
Metabolic syndrome							
	Metabolic syndrome	Inverse variance weighted	-0.19	8.37×10^{-6}	1851.05	1.34×10^{-299}	TRUE
		MRlap	-0.23	1.01×10^{-6}	–	–	–
		MR LASSO	-0.05	2.66×10^{-4}	–	–	–
Osteoporosis							
	Femoral neck BMD	Inverse variance weighted	-0.21	7.01×10^{-5}	186.12	3.97×10^{-3}	TRUE
		MRlap	-0.30	3.00×10^{-7}	–	–	–
		MR LASSO	-0.23	2.45×10^{-8}	–	–	–
	Heel_BMD	Inverse variance weighted	-0.16	3.02×10^{-8}	1152.81	1.15×10^{-159}	TRUE
		MRlap	-0.24	6.30×10^{-9}	–	–	–
		MR LASSO	-0.13	5.17×10^{-20}	–	–	–
	Lumbar Spine BMD	Inverse variance weighted	-0.26	2.85×10^{-6}	184.95	4.71×10^{-3}	TRUE
		MRlap	-0.36	4.50×10^{-8}	–	–	–
		MR LASSO	-0.29	1.15×10^{-10}	–	–	–
	Total_BMD	Inverse variance weighted	-0.16	3.84×10^{-4}	303.98	1.64×10^{-14}	TRUE
		MRlap	-0.22	1.67×10^{-5}	–	–	–
		MR LASSO	-0.19	5.25×10^{-10}	–	–	–
Biomarkers							
	Glycated haemoglobin HbA1c	Inverse variance weighted	-0.11	2.66×10^{-4}	874.20	2.56×10^{-107}	TRUE
		MRlap	-0.14	1.25×10^{-4}	–	–	–
		MR LASSO	-0.07	7.42×10^{-7}	–	–	–
	HDL cholesterol	Inverse variance weighted	0.10	1.79×10^{-5}	769.60	2.34×10^{-88}	TRUE
		MRlap	0.13	4.24×10^{-5}	–	–	–
		MR LASSO	0.09	6.20×10^{-11}	–	–	–
	Apolipoprotein A	Inverse variance weighted	0.11	3.65×10^{-5}	589.58	4.16×10^{-57}	TRUE
		MRlap	0.13	3.31×10^{-5}	–	–	–
		MR LASSO	0.08	3.80×10^{-9}	–	–	–
	Alanine aminotransferase	Inverse variance weighted	-0.09	2.09×10^{-4}	591.36	2.10×10^{-57}	TRUE
		MRlap	-0.12	4.37×10^{-5}	–	–	–
		MR LASSO	-0.07	3.04×10^{-8}	–	–	–

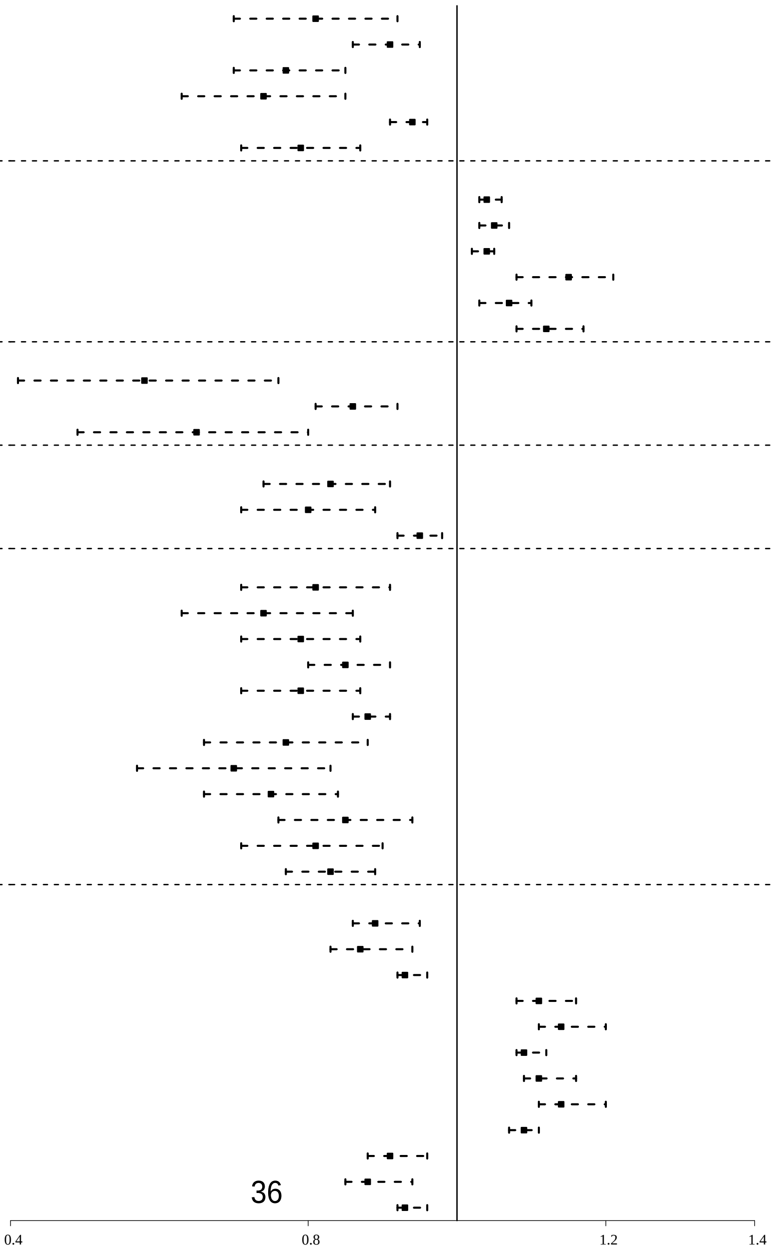

Supplementary Figure 34: Significant casual effects for mvPuberty on adulthood traits and biomarkers using Mendelian randomization analysis . *P* values are derived from Mendelian randomization tests for mvPuberty on adulthood traits and biomarkers, and are surpassing the Bonferroni adjusted threshold. BMD, Bone mineral density. MR LASSO, MR Least Absolute Shrinkage and Selection Operator. mvAge, multivariate GWAS of age. HDL, High-Density Lipoprotein. Data available in Supplementary Data 22.

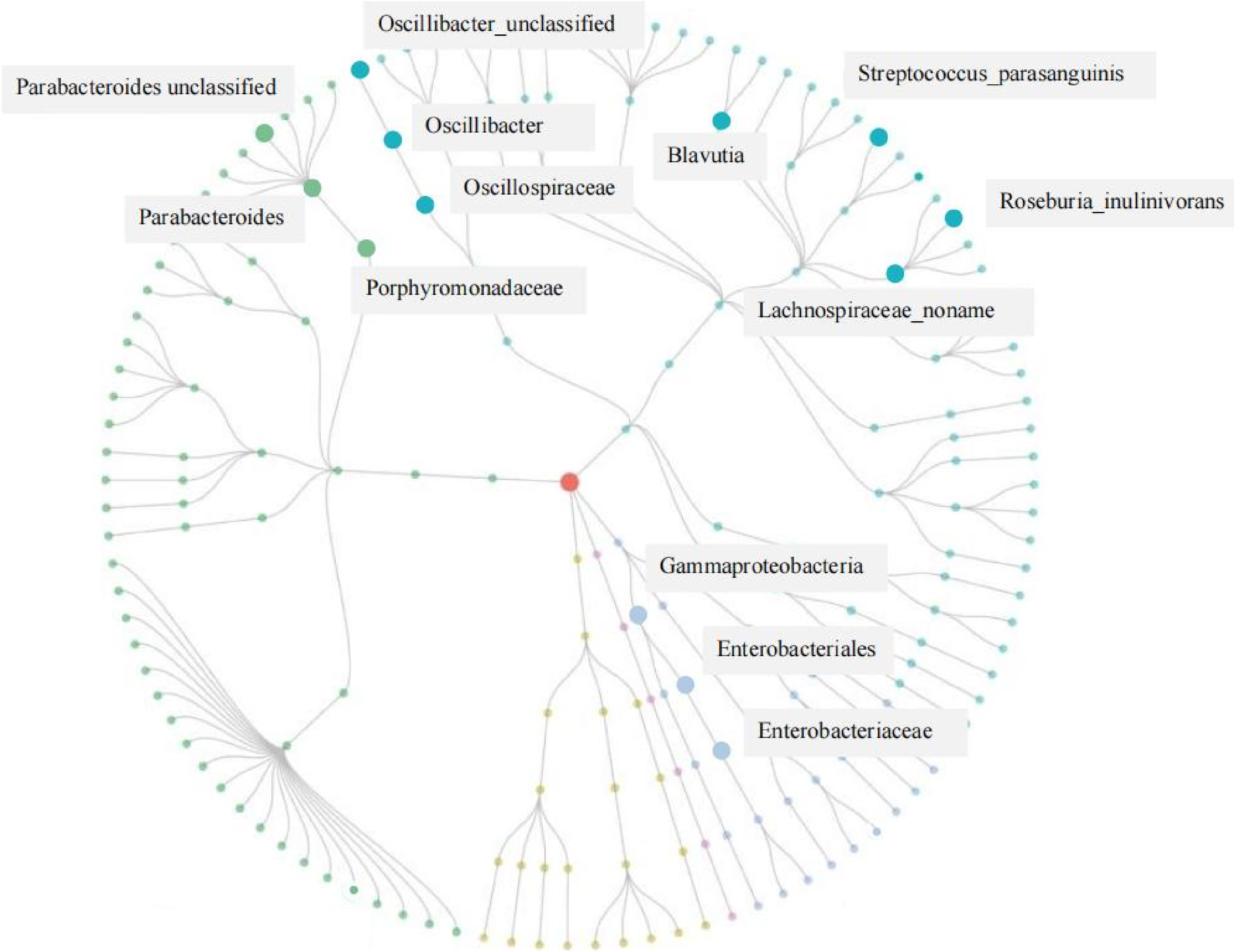

Taxa	Methods	NSNPs	β	SE	*P*
s. Parabacteroides unclassified	Inverse variance weighted	137	-0.42	0.12	7.22×10^{-4}
g. Parabacteroides	Inverse variance weighted	137	-0.26	0.10	6.38×10^{-3}
f. Porphyromonadaceae	Inverse variance weighted	137	-0.23	0.09	9.85×10^{-3}
s. Oscillibacter unclassified	Inverse variance weighted	137	-0.24	0.10	2.14×10^{-2}
f. Oscillospiraceae	Inverse variance weighted	137	-0.23	0.10	2.36×10^{-2}
g. Oscillibacter	Inverse variance weighted	137	-0.23	0.10	2.38×10^{-2}
o. Enterobacteriales	Inverse variance weighted	137	0.22	0.10	3.10×10^{-2}
f. Enterobacteriaceae	Inverse variance weighted	137	0.22	0.10	3.10×10^{-2}
s. Roseburia_inulinivorans	Inverse variance weighted	137	-0.21	0.10	3.31×10^{-2}
g. Blautia	Inverse variance weighted	137	-0.19	0.09	4.25×10^{-2}
g. Lachnospiraceae_noname	Inverse variance weighted	137	-0.18	0.09	4.61×10^{-2}
s. Streptococcus_parasanguinis	Inverse variance weighted	137	-0.39	0.20	4.72×10^{-2}
c. Gammaproteobacteria	Inverse variance weighted	128	0.19	0.10	4.87×10^{-2}

Supplementary Figure 35: Mendelian randomization analysis on the causal effect of the mvPuberty on gut microbiota. Data available in Supplementary Data 24.

Exposure	Outcome	Methods	β	P	Q	Q_pval	correct_causal_direction
HDL cholesterol	Coronary_artery_disease	Inverse variance weighted	-0.28	3.86×10^{-13}	1209.02	6.24×10^{-114}	TRUE
		MRlap	-0.09	1.33×10^{-14}	–	–	–
		MR LASSO	-0.31	7.01×10^{-39}	–	–	–
	Heart_failure	Inverse variance weighted	-0.10	1.37×10^{-3}	676.42	2.21×10^{-30}	TRUE
		MRlap	-0.02	2.81×10^{-5}	–	–	–
		MR LASSO	-0.10	4.65×10^{-6}	–	–	–
	Heel_BMD	Inverse variance weighted	-0.08	1.85×10^{-5}	3562.33	1.00×10^{-100}	TRUE
		MRlap	-0.07	4.09×10^{-5}	–	–	–
		MR LASSO	-0.03	5.83×10^{-6}	–	–	–
	mvAge	Inverse variance weighted	0.04	6.89×10^{-14}	900.59	1.17×10^{-69}	TRUE
		MRlap	0.03	2.14×10^{-12}	–	–	–
		MR LASSO	0.04	1.14×10^{-38}	–	–	–
	Parental_lifespan	Inverse variance weighted	0.15	5.29×10^{-14}	972.43	3.57×10^{-76}	TRUE
		MRlap	0.05	2.95×10^{-13}	–	–	–
		MR LASSO	0.13	2.83×10^{-27}	–	–	–
	Total_BMD	Inverse variance weighted	-0.06	9.68×10^{-3}	619.02	7.13×10^{-22}	TRUE
		MRlap	-0.05	2.15×10^{-3}	–	–	–
		MR LASSO	-0.08	5.26×10^{-7}	–	–	–
Alanine aminotransferase	Coronary_artery_disease	Inverse variance weighted	0.24	3.76×10^{-4}	835.60	1.17×10^{-95}	TRUE
		MRlap	0.07	3.87×10^{-3}	–	–	–
		MR LASSO	0.19	$6.77e\times10^{-8}$	–	–	–
	mvAge	Inverse variance weighted	-0.05	1.41×10^{-9}	378.25	1.08×10^{-24}	TRUE
		MRlap	-0.04	8.04×10^{-7}	–	–	–
		MR LASSO	-0.03	7.70×10^{-12}	–	–	–
	Parental_lifespan	Inverse variance weighted	-0.08	3.43×10^{-2}	749.67	6.81×10^{-82}	TRUE
		MRlap	-0.03	6.83×10^{-2}	–	–	–
		MR LASSO	-0.09	3.31×10^{-7}	–	–	–
Apolipoprotein A	Coronary_artery_disease	Inverse variance weighted	-0.19	5.22×10^{-6}	1095.33	4.15×10^{-127}	TRUE
		MRlap	-0.06	3.53×10^{-6}	–	–	–
		MR LASSO	-0.21	1.80×10^{-21}	–	–	–
	Heart_failure	Inverse variance weighted	-0.06	2.75×10^{-2}	460.05	3.58×10^{-22}	TRUE
		MRlap	-0.01	1.49×10^{-2}	–	–	–
		MR LASSO	-0.05	3.66×10^{-2}	–	–	–
	mvAge	Inverse variance weighted	0.02	1.23×10^{-5}	652.96	7.27×10^{-57}	TRUE
		MRlap	0.02	1.38×10^{-5}	–	–	–
		MR LASSO	0.02	2.86×10^{-12}	–	–	–
	parental_lifespan	Inverse variance weighted	0.10	2.71×10^{-6}	856.97	1.15×10^{-86}	TRUE
		MRlap	0.04	1.59×10^{-6}	–	–	–
		MR LASSO	0.07	1.33×10^{-10}	–	–	–
Glycated haemoglobin HbA1c	Coronary_artery_disease	Inverse variance weighted	0.22	1.06×10^{-12}	709.90	1.21×10^{-50}	TRUE
		MRlap	0.07	2.17×10^{-10}	–	–	–
		MR LASSO	0.21	5.34×10^{-28}	–	–	–
	mvAge	Inverse variance weighted	-0.04	2.19×10^{-14}	568.78	9.14×10^{-34}	TRUE
		MRlap	-0.03	2.14×10^{-10}	–	–	–
		MR LASSO	-0.03	3.09×10^{-27}	–	–	–
	parental_lifespan	Inverse variance weighted	-0.07	7.02×10^{-8}	359.22	3.13×10^{-8}	TRUE
		MRlap	-0.02	1.28×10^{-4}	–	–	–
		MR LASSO	-0.08	1.54×10^{-13}	–	–	–
g.Parabacteroides	Lumbar Spine BMD	Inverse variance weighted	0.10	1.66×10^{-2}	4.95	$8.39\times10^{-}1$	TRUE
	Total_BMD	Inverse variance weighted	0.06	1.28×10^{-2}	16.94	4.97×10^{-2}	TRUE

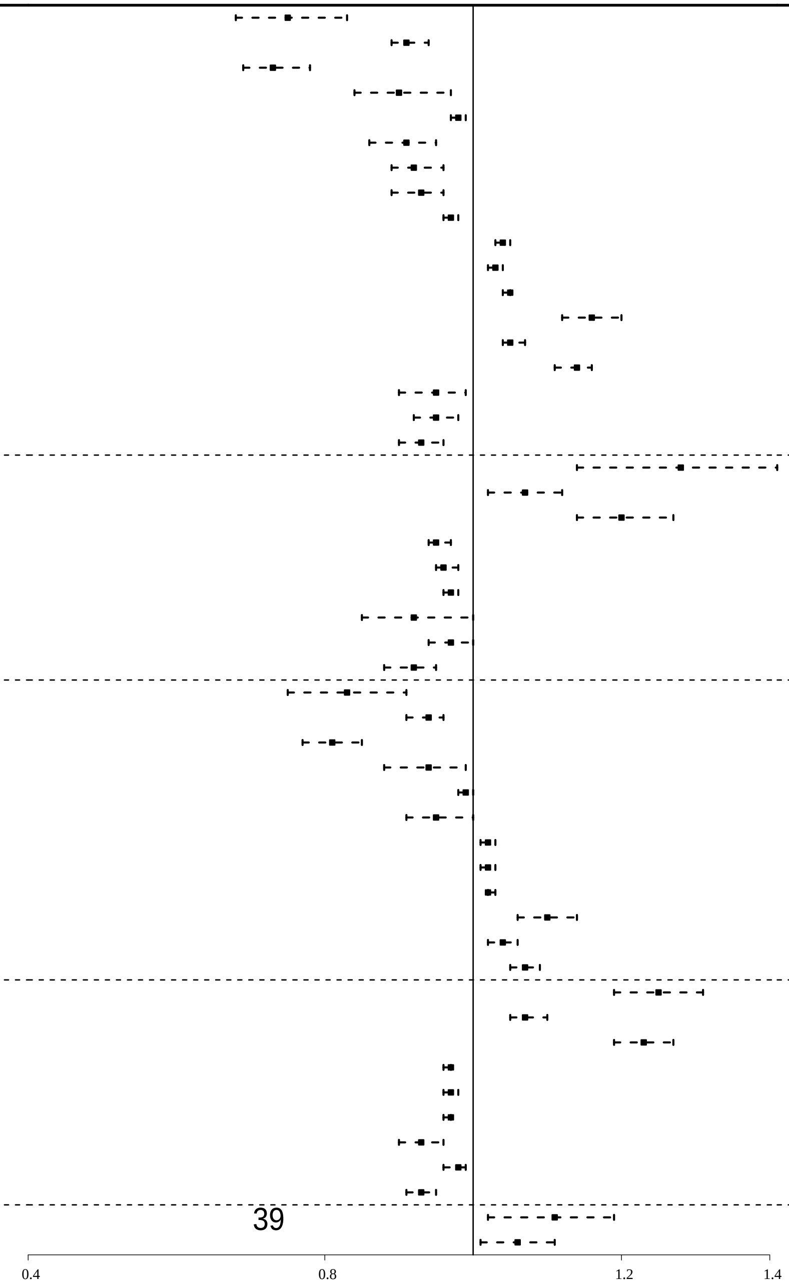

Supplementary Figure 36: Significant casual effects for biomarkers on adulthood traits using Mendelian randomization analysis. BMD, Bone mineral density. P values are derived from Mendelian randomization tests for each biomarker on adulthood traits, and are surpassing the Bonferroni adjusted threshold. BMD, Bone mineral density. MR LASSO, MR Least Absolute Shrinkage and Selection Operator. mvAge, multivariate GWAS of age. HDL, High-Density Lipoprotein. Source data are provided as Supplementary Data 25-27.

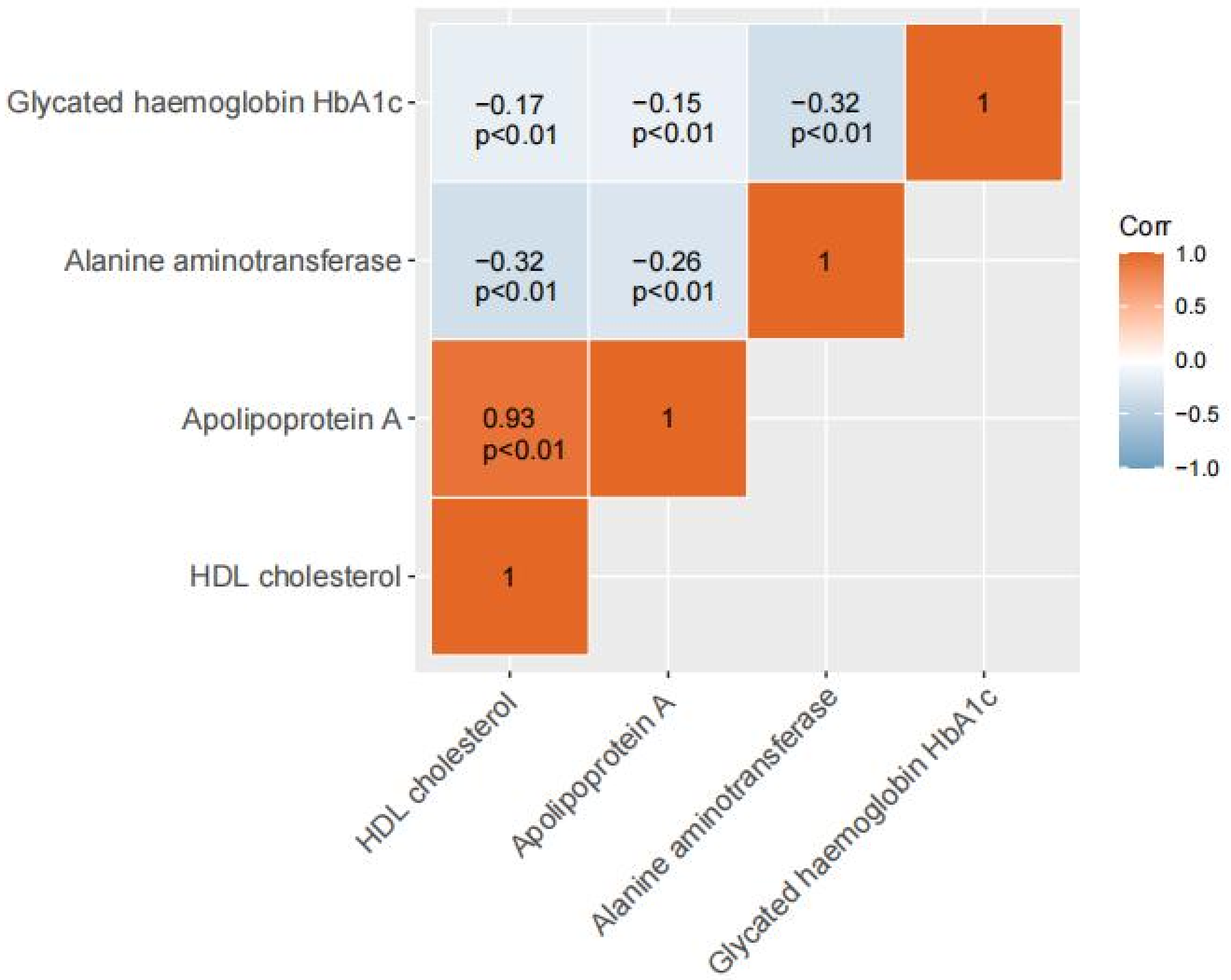

Supplementary Figure 37: Heatmap for genetic correlation for four biomarkers.
Genetic correlations for MR-BMA analysis, displaying pairwise LD score genetic correlation estimates for the four biomarkers.

Supplementary methods

Genomic structural equation modeling (Genomic SEM) background and overview.

Genomic SEM is a multivariate method for analyzing the joint genetic architectures of complex traits. Using formal methods for modeling covariance structure, genomic SEM synthesizes genetic correlations and SNP-heritabilities inferred from GWAS summary statistics of individual traits from samples with varying and unknown degrees of overlap[1]. Genomic SEM can be used to identify variants with effects on general dimensions of cross-trait liability, boost power for discovery, and calculate more predictive polygenic scores. Finally, Genomic SEM can be used to identify loci that cause divergence between traits, aiding the search for what uniquely differentiates highly correlated phenotypes. SEM is a two-stage application for methods that examine the variance and covariance structure among groups of related variables to GWAS studies. Genomic SEM has been shown to be robust to both differences in sample-sizes of the input GWAS and sample overlap of the input GWASs, enhancing applicability and facilitating the capability to improve statistical power from increased effective sample sizes[1]. Therefore, we used Genomic SEM to first model the joint genetic architecture of puberty timing, jointly analyzing GWASs from four genetically correlated puberty timing related phenotypes; and then generate a mvGWAS, identifying individual SNP associations for this general latent puberty timing factor. Relevant principles and details of SEM in the context of Genomic SEM were showed on the original Genomic SEM methods paper[1].

Genomic SEM analysis for our study

Genomic SEM included two stages, using the measurement and structural model framework to model the genetic covariances across puberty timing GWAS phenotypes. In stage 1, Genomic SEM estimates the empirical genetic covariance matrix sampling covariance matrix using the linkage disequilibrium (LD) score regression analysis, modified to account for possible sample overlap[1]. In stage 2, parameters of the

user-specified SEM model are estimated with either weighted least squares (WLS) or maximum likelihood (ML) estimators using the genetic covariance matrix (from stage 1)[1]. WLS and ML estimators weigh the matrix information differently, but both estimators minimize the fit error between the model-implied and empirical genetic covariances[1]. We use the WLS estimator as recommended and used in recent Genomic SEM GWAS studies[2,3]. WLS optimizes the fit function by using the diagonal elements in the sampling covariance matrix and adjusting standard errors of the estimates with the off-diagonal elements, indices for the correlations among the sampling errors of the summary statistics[2]. These features make Genomic SEM unbiased and robust up to 100% sample overlap and also unbalanced GWAS sample sizes[1].

Assessing model fit

Model fit was evaluated using conventional SEM indices, including the model χ^2 statistics, the Akaike information criterion (AIC), comparative fit index (CFI), and the standardized root mean square residual (SRMR)[2]. Apart from the χ^2 statistic, each index uses its standard SEM interpretation. Large sample sizes common in GWAS analysis may overpower the χ^2 test, increasing the likelihood of it being statistically significant. As suggested by the developers of Genomic SEM, we used the model χ^2 statistic as comparative measure of fit, not as a measure of statistical significance. Following guidelines in use, we considered CFI and SRMR values of > 0.90 and < 0.08 as indicating good fit[2].

Effective sample size calculation

The Genomic SEM method is robust to sample overlap, making it applicable to our analyses given the overlap across cohorts included in the four puberty timing related GWASs included (UK Biobank cohorts were included in the age of voice break, age of first facial hair, and other smaller cohorts were potentially included in more than one GWAS used in our analysis). We estimated the effective sample sizes for each SNP included in the mvPuberty GWAS using steps prescribed previously[1,2,4]. Given

the multivariate GWAS effect estimate for SNP j,

$$\beta_j = \frac{Z_j}{\sqrt{n_j \times 2 \times \mathrm{MAF}_j \times (1 - \mathrm{MAF}_j)}}$$

(where Z_j is the multivariate GWAS association statistic for SNP_j, n_j is the effective sample size for SNP_j that we aim to calculate, MAF_j is the minor allele frequency of SNP_j; the SNP_j variance (σ_j^2) is $2 \times \mathrm{MAF}_j \times (1\text{-}\mathrm{MAF}_j)$)), and rearranging,

$$\boldsymbol{n_j} = \frac{\left(\boldsymbol{Z_j}/\boldsymbol{\beta_j}\right)^{\mathbf{2}}}{\boldsymbol{\sigma_j}^{\mathbf{2}}}$$

Effective sample size N_{eff} is taken to be approximately equal to the mean n_j for SNPs meeting the MAF thresholds (restricted in our analyses, as recommended, to MAF between 10% and 40% because the effective calculations are inflated):

$$N_{eff} \approx \frac{1}{m} \sum_{MAF}^{b} = a^{n_j}$$

We apply this formula to estimate the effective sample size for the latent externalizing factor, yielding N_{eff} of 514,750.

Heterogeneity testing

Genomic SEM was used to perform SNP-level tests of heterogeneity (Q_{SNP}) to investigate whether each SNP had consistent, pleiotropic effects on the four input phenotypes that effectively only operate via the shared genetic liability for puberty timing[3]. Q_{SNP} are estimated for each lead mvPuberty SNP (independent SNP associated with mvPuberty with P-value < 5×10^{-8}), which are χ^2-distributed test statistics. The null hypothesis of the QSNP test is that SNP effects on the constituent phenotypes are completely mediated via a shared pathway through the mvPuberty factor, so a significant QSNP test indicates that a given SNP's effects are better explained by trait-specific pathways independent of the mvPuberty factor[1]. Based upon previous multivariate GWASs generated using Genomic SEM[2,4], we evaluated Q_{SNP} heterogeneity using a Bonferroni adjusted P-value threshold of 1.88×10-4 (0.05/266 lead SNPs). Out of 266 lead SNPs, 27 were heterogeneous, suggesting that the majority of the mvPuberty lead SNPs associations explained best by a shared

causal pathway (Supplementary Table 6). We estimated mean $\chi 2$ and genomic inflation factor (λ_{GC}) of the Q_{SNP} results to be 1.98 and 1.60, respectively, when restricted to the 1,016,650 SNPs used in LD score regression. Thus, the Q_{SNP} analysis was sufficiently powered to identify substantial heterogeneity across the genome, but reassuringly, not with respect to the vast majority of our main findings. This aligns with the expectation of modelling a latent common factor with SEM, which is that the common factor should primarily identify shared variance and not the unique features of the model indicators. Finally, we estimated an LD score regression intercept of 0.997 (se = 0.024), which suggests that the inflation we observed in the Q_{SNP} test statistic is not attributable to bias from population stratification.

Genomic risk loci and functional annotation of GWAS results

FUMA (see URLs) is an online platform that annotates SNPs to their biological functionality and maps implicated genes. Biological functionality is characterized for lead SNPs and SNPs in LD using potential regulatory functions (RegulomeDB score), deleteriousness score (CADD score), effects on gene functions (using ANNOVAR), and mRNA expression levels (using eQTL and chromatin interaction data)[5].

Prior to annotation, FUMA first defines independent significant SNPs which have a genome-wide significant p-value (5×10^{-8}) and are independent at $r^2<0.6$. Based on LD information from UK Biobank genotypes, a subset of these independent significant SNPs is labeled as lead SNPs (independent from each other at $r^2<0.1$). If LD blocks of lead SNPs are less than 250 kb apart, then they are merged. We annotated all SNPs in LD with the most significant SNP to get insight into the possible biological reasons for observing a statistical association, i.e., annotation was performed for all SNPs that were in LD ($r^2>0.6$) with one of the independent significant SNPs, had a p-value lower than 1×10^{-5}, and a minor allele frequency (MAF) > 0.0001.

Fine-mapping

We performed fine-mapping to identify the most plausible causal variants in the

genomic loci associated with mvPuberty. We used five fine mapping methods – ABF, PAINOR, CAVIARBF, FINEMAP and SuSiE – implemented in the python pipeline, easyfinemap (version 0.4.6; see URLs). Easyfinemap automates fine mapping of GWAS summary statistics and facilitates comparison of nominated putatively causal variants across fine-mapping methods, which is important as each fine mapping method with complementary strengths and weaknesses.

ABF is a single-causal-variant fine-mapping method where only one causal variant is modeled in each fine-mapped region. It is most commonly applied when only summary statistics are available since it does not require LD[6]. However, ABF has a relatively higher replication failure rate than multiple fine-mapping methods based on sample LD or genotype/phenotype data[6]. PAINTOR is a statistical fine-mapping method that integrates functional genomic data with association strength from potentially multiple populations (or traits) to prioritize variants for follow-up analysis[7]. The software runs on multiple fine-mapping loci and/or populations/traits simultaneously and takes as input the following data for each set of SNPs at a locus[7].

CAVIAR takes as input the association statistics for all of the SNPs (variants) at the locus together with the correlation structure between the variants obtained from a reference data set[8]. The key idea behind CAVIAR is that instead of considering each variant one at a time, CAVIAR instead analyze all of the variants in the entire locus simultaneously. The result of CAVIAR is a set of variants that with high probability contains (or captures) all the causal variants. Through extensive simulation results, CAVIAR is superior to existing methods in reducing the overall number of variants to be examined in functional follow-up to identify the causal variants[8]. High linkage disequilibrium (LD) between genetic variants makes identifying true causal variants challenging and SuSIE ("SUm of SIngle Effects") extends Bayesian variable selection in regression (BVSR) applied to fine-mapping by applying an iterative Bayesian stepwise selection (IBSS) process (in contrast with a traditional stepwise selection method) and producing credible sets of variants that quantify the uncertainty

regarding the selection of a particular variant among the set of highly correlated variants[9]. By contrast, FINEMAP leverages a Shotgun Stochastic Search (SSS) algorithm, which is based upon the Markov Chain Monte Carlo (MCMC) algorithms used in applications of Bayesian inference, and for each locus with p SNPs (and a corresponding 2p possible causal models), efficiently evaluates neighboring models at each iteration (compared to search strategies employed by CAVIAR and other early fine-mapping methods)[10]. In addition, easyfinemap also supports function-based fine-mapping using PolyFun. PolyFun estimates prior causal probabilities for SNPs, which can then be used by fine-mapping methods like SuSiE or FINEMAP. Unlike previous methods for functionally-informed fine-mapping, PolyFun can aggregate polygenic data from across the entire genome and hundreds of functional annotations.

Therefore, in our study, to avoid the false positive rate for single fine-mapping method, we combine the results of these five approaches to identify the most plausible causal variants[11]. Combined PolyFun and SuSiE or FINEMAP analysis also was conducted to further supports the results of LD-free and LD-based fine-mapping approaches. We used the same 1000 Genomes Phase 3 reference panel that was used to generate the mvPuberty summary data in Genomic SEM. PAINTOR, CAVIAR, SuSIE and FINEMAP methods each generate a posterior probability that the variant is causal for mvPuberty. We used a 250 kilobase window (±250) kilobases around the lead SNP [SNP with smallest P-value in the locus]) and a stringent probability threshold of 0.95 to define credible sets of potentially causal variants.

Gene prioritization using transcriptomic imputation

Transcriptome-wide association study (TWAS) was conducted to further investigate gene-level associations of the mvPuberty genetic signature using the FUSION and UTMOST method. For our FUSION TWAS, pre-computed expression quantitative trait loci (eQTL), including 37,920 features, sourced from GTEx Version 8 downloaded from the FUSION website that leverage cross-tissue sparse canonical correlation (sCCA) models that integrate genetic features across multiple tissues to

increase TWAS power (especially when sample sizes for individual tissues used to derive the TWAS features are small), reduce testing burden, and when the disease-relevant tissue is not known[12]. The 1000 Genomes Project Phase 3 European for LD estimation was employed and genes located within the major histocompatibility complex (MHC; chromosome 6: given the complex LD structure of that region) were excluded according to FUSION protocol. After munging the mvPuberty summary statistics, there were 33,798 of the 37,920 sCCA features available in the mvPuberty data for analysis. The primary FUSION pipeline constitutes three steps: (1) identify gene expression features that are cis-heritable (i.e., variants associated with gene expression within or near the genomic locus); (2) construct a linear predictor for each cis-heritable gene (i.e., a SNP-based prediction weight of the gene feature); and (3) calculate both TWAS test-statistics incorporating these SNP-based prediction weights and summary-level GWAS Z-scores. FUSION uses penalized several linear regression and Bayesian sparse linear mixed models (e.g., GBLUP, LASSO, Elastic Net, BLSMM)[12] and computes an out-of-sample R^2 statistics to identify the best model via a cross-validation of each gene-GWAS model and we used Bonferroni corrected-statistical thresholds for determined by the number of genes tested across panels: $P < 1.48\times10^{-6}$ (0.05/33,798 sCCA features available for testing in mvPuberty) as a heuristic to facilitate our follow-up analysis on a plausible number of TWAS findings.

For our UTMOST PWAS, pre-computed single-tissue covariance matrices and cross-tissue imputation models, including 44 GTEx tissues, sourced from GTEx Version 8 downloaded from the UTMOST website[13]. Combine gene-trait associations in 44 tissues was conducted by joint generalized Berk-Jones (GBJ) test in 17,290 genes. After munging the mvPuberty summary statistics, there were 13,841 of the 17,290 GTEx features available in the mvPuberty data for analysis. The primary UTMOST pipeline constitutes two steps[13]: (1) Run single tissue association test for 44 tissues; (2); Combine gene-trait associations in 44 tissues by joint GBJ test. UTMOST

uses GBJ test with single-tissue association statistics Z and their covariance matrix as inputs to provide powerful inference results while explicitly taking the correlation among single-tissue test statistics into account even under a sparse alternative. We used Bonferroni corrected-statistical thresholds for determined by the number of genes tested across panels: $P < 3.61\times10^{-6}$ (0.05/13,841 GTEx features available for testing in mvPuberty) as a heuristic to facilitate our follow-up analysis on a plausible number of TWAS findings.

Subsequently, candidate genes were identified by intersecting the results of FUSION and UTMOST. We took each of the TWAS candidate genes associated with mvPuberty forward for additional analysis. FOCUS (Fine-mapping of causal gene setS), a fine-mapping method analogous to fine-mapping of GWAS results, using the same sCCA weights to identify potentially causal gene features within TWAS significant regions was conducted[14]. For each genetic region containing a TWAS-significant association, FOCUS controls for potential pleiotropic SNP effects and calculates the posterior inclusion probability (PIP) of each feature by summing the posterior probabilities so as to define a set of features with a strong likelihood of containing a causal feature (i.e., a 95% credible set)[14]. In FOCUS, individual feature PIP values > 0.5 are considered to suggest that the feature is more likely to be causal than any other regional feature[2].

Plasma protein prioritization using proteome imputation

Proteome-wide association study (PWAS) was conducted to further investigate protein-level associations of the mvPuberty genetic signature using the FUSION method. PWAS typically employs a two-stage framework[15]: Stage I uses the genetics and proteomics data of the same reference cohort to train a protein abundance prediction model for each target protein, taking the protein abundance quantitative trait as the response variable and the cis-SNPs proximal to the protein coding gene as predictors. The estimated SNP coefficients from Stage I can be viewed as effect sizes of “protein quantitative trait loci (pQTL)” in a broad sense, as most cis-SNPs with

non-zero effect sizes will not be statistically significant pQTL. Stage II proceeds by using the estimated pQTL effect sizes as variant weights to predict genetically regulated protein abundance in a GWAS cohort, and subsequently conducts a gene-based association test (of the corresponding protein coding gene) relating the predicted abundance of the target protein to phenotype[15].

For our FUSION PWAS, two pre-computed cis-protein quantitative trait loci (pQTL), including 2004 and 3154 features, sourced from Atherosclerosis Risk in Communities (ARIC) study and INTERVAL study, respectively[15,16]. After munging the mvPuberty summary statistics, there were 789 of the 2004, and 832 of the 3154 plasma protein features available in the mvPuberty data for analysis. The PWAS FUSION steps are consistent with the TWAS FUSION described above. PWAS FUSION uses Elastic Net model and we used Bonferroni corrected-statistical thresholds for determined by the number of genes tested across panels: $P < 6.34\times10^{-5}$ (0.05/789 ARIC features available for testing in mvPuberty) and $P < 6.01\times10^{-5}$ (0.05/832 INTERVAL features available for testing in mvPuberty) as a heuristic to facilitate our follow-up analysis on a plausible number of PWAS findings. Subsequently, candidate proteins were also forward for additional analysis for FOCUS[14]. In FOCUS, individual feature PIP values > 0.5 are considered to suggest that the feature is more likely to be causal than any other regional feature.

Gene set enrichment

MAGMA (Multi-marker Analysis of Genomic Annotation) with data from GTEx (version 8) was used to perform gene-based and gene-set analyses. MAGMA gene-based analyses included mapping SNPs to 18,649 protein coding genes within 10 kilobases of lead SNPs using several regression methods to account for LD between SNPs (using the 1000 Genomes European (EUR) sample as the reference)[17].

We used a Bonferroni corrected threshold of $P < 2.68\times10^{-6}$ to account for multiple coding genes comparisons. We also performed FUMA GENE2FUNC (gene to function) gene-set analyses using genes identified with MAGMA to evaluate potential

relationships between mvPuberty and lists of mapped genes from MSigDB gene sets[5], (i.e., Reactome, and Gene Ontology (GO)). We used an FDR 5% threshold correction to account for multiple testing to identify gene-sets enriched with mvPuberty.

Significant Cross-trait OUtliers and Trends in JOint York regression (SCOUTJOY)

We are interested in the question of whether there is a consistent relationship in the effect sizes for mvPuberty-associated loci across sex. Genetic correlation estimates can help quantify this relationship on the genome-wide level, but we are interested in also making this comparison among genome-wide significant loci. Our interest is not only in quantifying the average relationship, but in identifying whether the observed locus results for mvPuberty are consistent with a uniform relationship between male and female or if certain loci can be identified as outliers, potentially indicating differential roles or relative importance in each context.

SCOUTJOY, introduced by MR-PRESSO, use York regression to account for measurement error in the effect size estimates from both GWAS, including possible correlated error due to e.g. sample overlap, and extend outlier detection in cases where initial estimates of the trend in correlating effect size estimates may be unstable[18]. SCOUTJOY then evaluate both general over dispersion relative to a single trend line and whether individual SNPs can be identified as outliers relative to the effects seen for other SNPs in the group[18].

For our SCOUTJOY analysis, publicly available GWAS for Tanner stage, a measure of sexual maturation, divided into males and females to detect the difference for 266 mvPuberty-associated loci across sex[19]. The final results for SCOUTJOY analysis showed that mvPuberty-associated loci shoed generally concordant effect sizes (slope= 0.74, se=0.12) according to the original report[18]. In addition, only 4 loci were identified for outliers, which showed highly concordant across sex. Taken together these suggests that the relationship of mvPuberty-associated loci is not generally mediated by sex differences and instead the observed structure is consistent

across sex.

BrainXcan imputation

BrainXcan leverages the power of large-scale GWAS and reference brain image data to discover new mechanisms of disease etiology and validate existing ones[20]. BrainXcan tests the association with genetic predictors of brain image-derived features and complex traits to pinpoint relevant region-specific and cross-brain features. As this approach requires only genetic data, BrainXcan allows us to test a host of hypotheses on complex diseases, across many disorders and brain region modalities, using existing public data resources[20].

The BrainXcan framework is organized into three modules. The Prediction weight training module trains linear genetic predictors of brain features, tests for association between brain features and genotype and calculates the sample covariance of the genotypes[20]. For our BrainXcan analysis, pre-computed prediction weight, including 159 features derived from structural images representing total and gray matter volumes from different regions of the brain, and 300 diffusion MRI derived features representing neurite density, dispersion, and connectivity features, sourced from BrainXcan database[20].

The Association module will use the brain feature prediction weights and 'Reference LD' information we have shared to compute the BrainXcan association results table. The latter will provide estimated coefficients of the regression between the trait and the genetically predicted brain features, their standard error, and p-values[20]. For our study, the BrainXcan framework can be applied to mvPuberty GWAS that do not have the individual-level data available. This is because the association statistics can be inferred using the summary results from GWAS, the brain feature prediction weights, and the reference LD data generated by the first module[20]. The Mendelian randomization (MR) module performs a number of multiple instrument-based MR to determine the direction of the putative causal flow, i.e. whether alterations in brain features affect the complex trait or whether the trait (e.g.

disease status) alters brain features[20]. It provides bi-directional tests of causal flow and effect size scatter plots to help assess the consistency of the results. BrainXcan applied several MR approaches, including MR-BASE, inverse variance weighted regression, weighted median method, and Egger regression, to determine the direction of the putative causal flow, i.e. whether changes in image derived phenotypes (IDPs) are affecting changes in GWAS traits. In all analyses, BrainXcan assumed that multiple genetic variants are strongly associated with the phenotype (we refer to these as variants as "instruments"). SNPs with GWAS p-values $< 5 \times 10^{-8}$ for complex traits, and SNPs with GWAS p-values $< 1 \times 10^{-5}$ for IDPs were selected for BrainXcan. The less stringent p-value for IDPs was necessary to have a sufficient number of instruments, which can be adjusted as the number of individuals in the IDP dataset increases. To streamline the interpretations of the multiple MR results, BrainXcan combined the p-values of each MR output using an extension of the ACAT method, which accounts for concordance of the sign of the results. Results for all MR approaches are provided with the output of the software[20].

In brief, we focused on 261 features, which include 48 cortical gray matter volumes, 10 subcortical volumes, 13 subcortical gray matter volumes, fractional anisotropy (water diffusivity along nerve tracts) in 46 regions, ICVF (intracellular volume fraction) in 44 regions, OD (orientation dispersion) in 45 regions, and ISOVF (isotropic volume fraction) in 13 regions (note that features in the ISOVF category were less heritable leading to fewer successful predictors). We also included brain-wide measures represented by leading principal components of each subtype (gray volumes of cortical, cerebellum, and subcortical regions, subcortical total volumes, FA, ICVF, OD, and ISOVF). Bonferroni corrected threshold of $P < 0.05/261$ to account for multiple brain feature comparisons.

LD score regression

Identifying genetic correlations between complex traits and diseases can provide useful etiological insights and help prioritize likely causal relationships[21]. LD score

regression, widely used for estimating genetic correlation, requires only GWAS summary statistics and is not biased by sample overlap[21]. Therefore, genetic correlations (rg) were calculated between mvPuberty and 78 health-related traits used as outcomes in MR analysis using LD score regression. LD score regression estimates correlation between true causal effects of two traits (ranging from -1 to 1)[21]. SNPs in a high LD region would have a larger $\chi 2$, and a similar relationship would appear when single-study statistics are replaced by the product of z-scores from two studies of traits with correlation[21]. We performed LD score regression with unconstrained intercept using pre-computed LD scores from the 1000 Genomes Project Phase 3 (European ancestry), and restricted our analysis to only Haplotype map 3 (HapMap3) SNPs to minimize bias of low imputation quality[21]. We set Bonferroni-corrected significant threshold at a P value = 6.41×10^{-4} (0.05/78).

Mendelian randomization (MR)

MR assumptions

MR uses single nucleotide polymorphisms (SNPs) as instrumental variables to find associations between the genetic liability for an exposure trait and an outcome. The main assumptions underlying MR are (1) SNP instruments are robustly associated with the exposure, (2) SNP instruments are independent of confounders, and (3) SNP instruments influence the outcome only through the exposure. In the following sections, we describe the MR methods used in the study and how the methods are implemented to test these assumptions[22].

MR with complex traits

Epidemiological or observational studies have shown evidence that growth patterns during puberty are associated with health outcomes later in life, including adverse cardiovascular health[23], cancer[24,25], bone outcomes such as lower bone mineral density in later life—a risk factor for osteoporosis[26,27], gastrointestinal tract disorders, psychiatric diseases[28], neurodegenerative diseases[28], renal disorders and metabolic syndrome[29]. To investigate whether childhood later health was causally influenced by

our multivariate GWAS, we performed MR with 56 diseases derived from GWASs (European ancestry), including cancers, cardiovascular diseases, psychiatric diseases, gastrointestinal tract disorders, osteoporosis, neurodegenerative diseases, renal disorders and metabolic syndrome. And GWASs for aging and multi-organ development also were included in this analysis, due to the closely relationship with puberty timing. The analysis to identify causal effects form puberty timing may inform public health initiatives, interventions, and prevention strategies to reduce impact of irregular puberty onset. We outline below our motivation for selecting these diseases and developmental states.

Cancers

Cancer has progressively become the world's leading cause of mortality, imposing substantial disease burdens[30]. Early detection is crucial in preventing the progression of tumors[31]. Because puberty acts as a key early stage for human growth, in our studies, 20 types cancers were selected as outcomes for MR analysis, including bladder cancer, breast cancer, cervical cancer, colorectal cancer, corpus uteri cancer, endometrial cancer, esophagus cancer, gastric cancer, head and neck malignant neoplasia, renal cell carcinoma, lung cancer, malignant melanoma, non-hodgkin lymphoma, oral cavity cancer, oropharynx cancer, ovarian cancer, pancreatic cancer, prostate cancer, thyroid cancer. Detailed information showed in Supplementary Table 29.

Cardiovascular diseases

Cardiovascular disease (CVD) is the leading cause of death globally, and the population incidence of CVD and related metabolic disorders is higher in low- and middle-income countries than in the rest of the world[32]. Growth patterns in early life, such as birth weight and childhood BMI, are important predictors of adult CVD risk factors[32]. Understanding when in childhood growth relates to later CVD risk may guide the timing of interventions to prevent disease[32]. Therefore, 5 types common CVD were included in our MR analysis (these is atrial fibrillation, coronary artery

disease, heart failure, ischemic stroke, venous thromboembolism). Detailed information showed in Supplementary Tables 29.

Osteoporosis

Puberty is a time of dramatic changes in body size and composition, driven by a complex hormonal cascade that leads to rapid weight and height gain, the timing of which varies from person to person. And greater height and weight growth during 'pre-puberty' and 'post-puberty' were positively associated with bone size, a predictor of fracture risk, at 60–64 years[27]. Given relation between the timing of puberty and later bone outcomes, bone mineral density in 5 areas (femoral neck, lumbar spine, heel, skull and total body) and fracture were collected to explore the causal effects on mvPuberty. Detailed information showed in Supplementary Tables 29.

Psychiatric diseases and neurodegenerative diseases

Early pubertal timing is a risk factor for psychopathology during adolescence. Childhood who go through puberty earlier than their peers are at greater risk for internalizing and externalizing problems, including depression, anxiety, increased stress, aggression, and suicidal thoughts and behaviors[28]. Genetic correlation between psychiatric diseases and neurodegenerative diseases has been found in many genetic studies[33]. Given the relative comprehensiveness of current GWAS data on psychiatric diseases and neurodegenerative diseases and the strong correlation shown by observational studies between the puberty timing and psychiatric diseases[34], 11 psychiatric diseases and 4 and neurodegenerative diseases included in our MR studies (insomnia, attention deficit hyperactivity disorder, autism spectrum disorder, post-traumatic stress disorder, schizophrenia, bipolar disorder, anxiety, anorexia nervosa, depression, Tourette syndrome and obsessive compulsive disorder; amyotrophic lateral sclerosis, Parkinson's disease, Lewy body disease, and Alzheimer's disease). Detailed information showed in Supplementary Tables 29.

Renal disorders and gastrointestinal tract disorders

Previous studies have demonstrated that pubertal onset may be associated with more

rapid progression of kidney disease, such as glomerular filtration rate declining after pubertal onset[35,36]. In addition, increase in luteinizing hormone is a more objective marker of pubertal onset, and activation of the HPG-axis via pulsatile increase in various pubertal hormones occurs before certain physical manifestations of puberty such as growth spurt[35]. However, alterations in the HPG axis may impaired kidney metabolism and intestinal absorption. Therefore, in our studies, considering the small sample size of GWAS data on renal disorders, this study only included chronic kidney disease and acute tubulo interstitial nephritis to conduct MR analysis. For gastrointestinal tract disorders[37], we selected the GWAS, used in our previous studies, to investigate whether it is affected by puberty timing. Detailed information showed in Supplementary Tables 29.

Metabolic syndrome

The observation evidence that timing of puberty is associated with metabolic risk has been overwhelming and was observed across different ethnic backgrounds[38]. This observation is also supported by GWAS, which identify shared etiologies between puberty timing and markers for metabolic syndrome[38]. However, only the age of menarche in girls, acted as the measure of the timing of puberty, was used to explore shared genetic architecture between puberty timing and metabolic syndrome[38]. In our studies, we used the current the largest GWAS data for metabolic syndrome to explore whether it is affected by puberty timing. Detailed information showed in Supplementary Tables 29.

Aging related phenotypes

Enabling older adults to remain independent and maintain sufficient quality of life is a significant ongoing challenge facing aging populations. Previous cross-sectional observational studies showed pubertal maturation may accelerate to increase opportunities to reproduce before the individual dies or becomes compromised, potentially at the expense of investments in adult health and longevity. Early pubertal timing has been associated with higher morbidity and earlier mortality[39] and with

accelerated biological aging as indexed by epigenetic clocks[39] and telomere length[39]. In meta-analysis, early pubertal timing was associated with accelerated cellular aging, as indexed by both epigenetic age and telomere length[40]. However, the genetic effects between puberty timing and aging are largely unknown. Because the focus of our downstream analyses is to reduce impact of irregular puberty onset, we focused our aging related phenotypes MR analyses on outcomes that commonly measure in studies. Therefore, we included outcomes related to healthspan, parental lifespan, longevity, frailty, phenoAge acceleration, Hannum age acceleration, grimAge acceleration, estimate levels of plasminogen activation inhibitor 1, intrinsic epigenetic age acceleration and estimated proportion of granulocytes which all reported in recently studies.

The healthspan GWAS consists of 300,477 unrelated, British-ancestry individuals from UK Biobank. The statistics were calculated by fitting Cox-Gompertz survival models with events defined as the first incidence of one of seven specific diseases (any cancer, diabetes, myocardial infarction, stroke, chronic obstructive pulmonary disease, dementia, and congestive heart failure) or death itself. As such, healthspan is highly dependent on the characteristics of the UK Biobank cohort, who were aged 40-69 years when they were recruited in 2006-2010 and of which two-thirds have yet to experience an age-related disease. Therefore, loci of healthspan GWAS have overrepresented effects on diseases of middle age (cancer, heart disease, etc) than age-related disease, which is a limitation acknowledged in the original study.

The parental lifespan GWAS consists of unrelated, European-ancestry individuals reporting a total of 512,047 mothers' and 500,193 fathers' lifespans. The statistics for each participating cohort were calculated by fitting Cox survival models to father's and mother's survival separately, adjusted for subject sex, at least 10 principal components, and study-specific covariates such as genotyping batch and array. Therefore, loci of parental lifespan GWAS don't have overrepresented effects on diseases of middle age.

The longevity GWAS included 11,262/3484 cases surviving at or beyond the age corresponding to the 90th/99th survival percentile, respectively, and 25,483 controls whose age at death or at last contact was at or below the age corresponding to the 60th survival percentile.

And its multivariate genome-wide association GWAS for aging related phenotypes (mvAge) also was used to assess the causal effects to mvPuberty[2]. Detailed information showed in Supplementary Tables 29.

Organ volume/fat/iron

As described above, puberty is a critical stage in growth and development and influences bone density as well as physical development, but whether it affects the development of the body's organs is not known, so this study collects the volume and fat content of multiple body organs to further explore the effects of puberty on them. These organs include abdominal subcutaneous fat volume, liver volume, visceral fat volume, percent liver fat, kidney volume, pancreas volume, lung volume, spleen volume, percent pancreas fat, liver iron content a pancreas iron content. Detailed information showed in Supplementary Tables 29.

Polygenic Mendelian randomization methods

For our multivariate puberty timing exposure, we created genetic instruments using SNPs at conventional genome-wide significance $P < 5\times10^{-8}$ clumped at linkage disequilibrium $R^2 = 0.001$ (10,000 kb distance), using reference samples comprised of participants of European ancestry[22]. F-statistics was calculated for the instruments for our exposure, which statistics generally exceeded the conventional cutoff of 10, suggesting minimal bias from weak instruments[22]. We extracted instruments corresponding to the outcome SNPs from our multivariate puberty timing GWAS, harmonized exposure and outcome SNPs, and then performed MR analysis: inverse variance weighted (IVW) MR comprises our main method with additional complementary, robust methods developed to estimate consistent causal effects under weaker assumptions than IVW MR (allowing us to assess evidence of causal effects

and evaluate the sensitivity of the analyses to different patterns of violations of IV assumptions (MR Egger, weighted median, simple mode, and weighted mode)[22]. The Steiger directionality test was used to evaluate the causal direction, and the Cochran Q heterogeneity test was used to assess heterogeneity in instrument effects, as heterogeneity may indicate violations of IV assumptions[2]. If we found evidence of heterogeneity, we used the MR Lasso method, which applies lasso-type penalization to the direct effects of the instruments using a post-lasso estimate, and then performing IVW MR using only those instruments identified as valid instruments (tuning parameter specified at default heterogeneity stopping rule)[2].

We used a Bonferroni adjusted threshold P-value = 6.41×10^{-4} (0.05/78 diseases and growth status). Analyses were carried out using TwoSampleMR, version 0.6.2, and MendelianRandomization, version 0.10.0, in the R environment, version 4.3.2. These MR analyses have been reported in accordance with the STROBE-MR guidelines (Supplementary Checklist).

Sample independence

Our mvPuberty GWAS and many of disease outcomes GWASs are, at least in part, comprised of data from UK Biobank participants. Sample overlap between exposure and outcome datasets produce may bias IVW estimates in two-sample MR. Further, sample overlap impacts two other sources of potential bias in MR IVW estimates – weak instrument bias and winner's curse (which may happen when the same sample used to select the instruments are used as the exposure dataset). Simulation studies suggest that sample overlap bias is minimal when the MR instruments are strong (i.e., have large F-statistics [F-statistics >10])[2], and when overlapping samples come from large biobanks (i.e., the UK Biobank), suggesting that our results are minimally affected by this source of bias[3]. Nevertheless, we incorporate the MR Lap method, which was recently developed to account for sample overlap (even when the exact overlap percentage is unknown) and also assesses weak instrument bias and winner's curse, as an additional sensitivity test for our polygenic MR analyses[41]. Briefly, MR

Lap uses cross-trait LD score regression to evaluate approximate sample overlap and provide a corrected IVW estimate. Per the developer guidelines, we used the MR Lap method as a sensitivity analysis and report the MR Lap-corrected estimate if it was different than the IVW estimate[41].

The mediation effect of mvPuberty to adult traits

The above study found that aging, cardiovascular disease, osteoporosis, endometrial cancer, and metabolic syndrome are affected by the puberty timing. Moreover, previous studies have shown that some circulating lipids, glycemic markers, blood pressure, immune cells, markers of inflammation, liver function and gut microbiota also affect the aging process, cardiovascular disease, osteoporosis and endometrial cancer[2,42-44]. These observations suggest that these biomarkers may mediate the relationship between puberty timing and adult traits. Therefore, our study further investigates whether puberty timing affects these significant traits by influencing the levels of these biomarkers.

According to previous studies, we collected 34 types biomarkers (8 circulating lipids, 2 glycemic markers, 2 endocrine function markers, 6 kidney function markers, 5 liver function enzymes, 2 liver iron status, 8 inflammation status markers and systolic blood pressure) and 207 bacterial taxa (5 phyla, 10 classes, 13 orders, 26 families, 48 genera, and 105 species). Detailed information showed in Supplementary Tables 30.

We performed a two-step MR analysis to determine the mediation effect of biomarkers on the associations between mvPuberty and adult traits and the potential role of biomarkers as mediators.

Firstly, we conducted two sample MR analysis, consistent with the MR analysis described above (including IVW, MR LASSO and MR Lap analysis), to find the mediators (biomarkers) using a Bonferroni adjusted threshold ($P = 0.05/34 = 1.47\times10^{-3}$ for biomarkers; $P = 0.05/207 = 2.42\times10^{-4}$ for gut microbiota). The results of this part were showed in Supplementary Tables 19-22. As a results, we found 4 biomarkers

(HDL-C, Alanine aminotransferase, Apolipoprotein A and HbA1c) surpassing correction for multiple comparisons. For gut microbiota, with no bacterial taxa surpassing correction for multiple comparisons, we only selected the most significant result was for the species *Parabacteroides* to act as a mediator for the next analysis. Secondly, the causal effects for mvPuberty on adult traits had been done in above MR analysis. Adult traits surpassing correction for multiple comparisons was selected to further explore the causal effects for biomarkers. Since metabolic syndrome is a clinical syndrome that seriously affects the health of the organism with the aggregated onset of obesity, hyperglycaemia and dyslipidemia, which overlapped with the mediators obtained so they were not included in the next analysis.

Subsequently, considering the high correlation among biomarkers (Supplementary Tables 23), we performed the multivariable MR to prioritize the most likely causal biomarkers. We utilized MR-BMA, a two-sample multivariable MR approach, which is capable of identifying true causal risk factors despite high correlations of candidate factors as previously described[45]. First, we performed MR analysis of the combination of multiple biomarkers using weighted linear regression models as in the IVW method and assessed the posterior probability of causality for each specific model based on a Bayesian framework. Then, for each of the candidate biomarkers, we summed up the posterior probability over all models where the candidate biomarkers are present, to compute its marginal inclusion probability (MIP), which represented the probability of being a causal biomarker for adulthood traits. Furthermore, the model-averaged causal estimate (MACE), which represents the average causal effect across models of each biomarker on adult traits, was also calculated. P-values were calculated for each biomarker using a permutation method, with adjustment for multiple testing via the Bonferroni procedure. Besides, we performed a LD score regression analysis to confirm the high genetic correlation between the biomarkers.

Finally, the proportion of the total effect mediated by biomarkers was estimated

by dividing the indirect effect by the total effect ($\beta1 \times \beta2/\beta3$)[46], which β1 representing the effect of mvPuberty on the biomarkers, β2 representing the effect of biomarkers on adult traits, and β3 representing the effect of mvPuberty on adult traits. Standard errors were derived using the bootstrap method and effect estimates were obtained from two-sample MR analysis.

MR with modifiable dietary factors.

Above analysis showed early onset of puberty is considered an essential factor on the life-course path to a number of diseases in adulthood, including cancers, aging, metabolic syndrome, and cardiovascular disease. And our previous studies found notable associations between dietary intakes and pubertal timing beyond contributions to an energy imbalance: children with the highest intakes of vegetable protein or animal protein experience pubertal onset up to 7 months later or 7 months earlier, respectively[47]. Therefore, to investigate whether our multivariate GWAS was causally influenced by dietary factors, we performed MR analysis with 26 dietary intake and micronutrients derived from GWASs (European ancestry). Detailed information showed in Supplementary Tables 31. Polygenic MR methods and sample independence were consistent with above analysis.

STROBE-MR Reporting Guidelines

1. TITLE and ABSTRACT

Indicate Mendelian randomization as the study's design in the title and/or the abstract.

Not applicable for title (GWAS study). MR discussed in abstract.

INTRODUCTION

2. Background

Explain the scientific background and rationale for the reported study. Is causality between exposure and outcome plausible? Justify why MR is a helpful method to address the study question.

Addressed in the Introduction and Methods and Supplementary Methods

3. Objectives

State specific objectives clearly, including pre-specified causal hypotheses (if any).

Addressed in the Introduction and Methods.

METHODS

4. Study design and data sources

Present key elements of study design early in the paper. Consider including a table listing sources of data for all phases of the study. For each data source contributing to the analysis, describe the following:

a) Describe the study design and the underlying population from which it was drawn. Describe also the setting, locations, and relevant dates, including periods of recruitment, exposure, follow-up, and data collection, if available.

b) Give the eligibility criteria, and the sources and methods of selection of participants.

c) Explain how the analyzed sample size was arrived at.

d) Describe measurement, quality and selection of genetic variants.

e) For each exposure, outcome and other relevant variables, describe methods of assessment and, in the case of diseases, the diagnostic criteria used.

f) Provide details of ethics committee approval and participant informed consent, if relevant.

Addressed in the Methods and Supplementary Methods.

7. Assessment of assumptions

Describe any methods used to assess the assumptions or justify their validity.

Addressed in the Methods and Supplementary Methods.

8. Sensitivity analyses

Describe any sensitivity analyses or additional analyses performed.

Addressed in the Methods and Supplementary Methods.

9. Software and pre-registration

a) Name statistical software and package(s), including version and settings used.

Addressed in the Methods.

b) State whether the study protocol and details were pre-registered (as well as when and where).

Addressed in the Methods and Supplementary Methods.

RESULTS

10. Descriptive data

a) Report the numbers of individuals at each stage of included studies and reasons for exclusion. Consider use of a flow-diagram.

b) Report summary statistics for phenotypic exposure(s), outcome(s) and other relevant variables (e.g. means, standard deviations, proportions).

c) If the data sources include meta-analyses of previous studies, provide the number of studies, their reported ancestry, if available, and assessments of heterogeneity across these studies. Consider using a supplementary table for each data source.

d) For two-sample Mendelian randomization:

i. Provide information on the similarity of the genetic variant-exposure associations between the exposure and outcome samples.

ii. Provide information on extent of sample overlap between the exposure and outcome data sources.

Addressed in the Methods, Results, Supplementary Results, Supplementary Tables.

11. Main results

a) Report the associations between genetic variant and exposure, and between genetic variant and outcome, preferably on an interpretable scale (e.g. comparing 25th and 75th percentile of allele count or genetic risk score, if individual-level data available).
b) Report causal effect estimate between exposure and outcome, and the measures of uncertainty from the MR analysis. Use an intuitive scale, such as odds ratio, or relative risk, per standard deviation difference.
c) If relevant, consider translating estimates of relative risk into absolute risk for a meaningful time-period.
d) Consider any plots to visualize results (e.g. forest plot, scatter plot of associations between genetic variants and outcome versus between genetic variants and exposure).
Addressed in the Results, Supplementary Results, and Supplementary Tables.
12. Assessment of assumptions
a) Assess the validity of the assumptions.
b) Report any additional statistics (e.g., assessments of heterogeneity, such as I2, Q statistic).
Addressed in the Results, Supplementary Tables, Discussion and Supplementary Discussion.
13. Sensitivity and additional analyses
a) Use sensitivity analyses to assess the robustness of the main results to violations of the assumptions.
b) Report results from other sensitivity analyses (e.g., replication study with different dataset, analyses of subgroups, validation of instrument(s), simulations, etc.).
c) Report any assessment of direction of causality (e.g., bidirectional MR).
d) When relevant, report and compare with estimates from non-MR analyses.
e) Consider any additional plots to visualize results (e.g., leave-one-out analyses).
Addressed in the Results, Supplementary Results, and Supplementary Tables.
DISCUSSION
14. Key results
Summarize key results with reference to study objectives.
Addressed in the Discussion and Supplementary Discussion.

15. Limitations

Discuss limitations of the study, taking into account the validity of the MR assumptions, other

sources of potential bias, and imprecision. Discuss both direction and magnitude of any

potential bias, and any efforts to address them.

Addressed in the Discussion and Supplementary Discussion.

16. Interpretation

a) Give a cautious overall interpretation of results considering objectives and limitations. Compare with results from other relevant studies.

b) Discuss underlying biological mechanisms that could be modelled by using the genetic variants to assess the relationship between the exposure and the outcome.

c) Discuss whether the results have clinical or policy relevance, and whether interventions could have the same size effect.

Addressed in the Discussion and Supplementary Discussion.

17. Generalizability

Discuss the generalizability of the study results (a) to other populations (i.e. external validity),

(b) across other exposure periods/timings, and (c) across other levels of exposure.

Addressed in the Discussion and Supplementary Discussion.

OTHER INFORMATION

18. Funding

Give the source of funding and the role of the funders for the present study and, if applicable, for the original study or studies on which the present article is based.

Addressed in the Funding.

19. Data and data sharing

Present data used to perform all analyses or report where and how the data can be accessed. State whether statistical code is publicly accessible and if so, where.

Addressed in the Methods.

20. Conflicts of Interest

All authors should declare all potential conflicts of interest.

Addressed in the Conflicts of interest.

Supplementary discussion

In our BrainXcan analysis, we found changes in the mean diffusivity and radial diffusivity of several white matter microstructures, including the superior corona radiata, superior longitudinal fasciculus, external capsule, and uncinate fasciculus, which affected emotional development [48-50]. These white matter microstructures were identified as possible subsequent neural signatures for advanced pubertal status due to abnormal changes in gonadal sex steroid hormones [48-50]. These findings indicate that adult emotional disorders may be attributed to changes in white matter microstructures caused by abnormal gonadal sex steroid hormones during puberty. In addition, we also detected changes in the mean diffusivity and radial diffusivity in two other white matter microstructures, the inferior cerebellar peduncle and cingulum cingulate gyrus, through BrainXcan analysis. The inferior cerebellar peduncle, which connects the cerebellum and other parts of the nervous system, plays an important role in locomotor and mental adaptation, age and brain lateralization and fatigue [51-53]. The cingulate gyrus, which connects the cingulate cortex with other brain regions, has also been associated with language, specifically phonological processing, including visuospatial episodic memory and spatial word memory [54,55]. Previous studies have shown that gonadal sex steroid hormones regulate neurogenesis and neuronal survival in the inferior cerebellar peduncle and cingulum cingulate gyrus. For example, oestradiol and progesterone facilitate oligodendrocyte activity and stimulate the proliferation of Schwann cells that produce myelin proteins, thereby modulating neurogenesis and neuronal survival through regulating each other's synthesis [56,57]. We found for the first time that the inferior cerebellar peduncle and cingulum cingulate gyrus are associated with pubertal timing, which could be attributed to the interaction between gonadal sex steroid hormones and neurogenesis and neuronal survival [48]. In summary, when the body secretes pubertal hormones, specifically gonadal hormones, at an earlier age and at higher levels, the brain may experience accelerated proliferation and subsequent fine-tuning of white matter development during puberty.

Future work incorporating longitudinal neuroimaging in parallel with pubertal measures may contribute to the understanding of individual variation in pubertal course and brain region development.

For cardiovascular diseases, we found that HDL-C levels mediated the causal effect of pubertal timing on the risk of developing coronary artery disease and heart failure. Observational, experimental, and genetic studies have shown that lower HDL cholesterol levels are associated with a greater risk of developing atherosclerosis and related cardiovascular complications [58-63]. HDL-C is intricately involved in cholesterol transport and inflammation modulation [64] and is associated with altered activity of the hypothalamus–pituitary–adrenal axis [65]. For example, early puberty causes abnormal secretion of sex hormones, affecting the expression of sex hormone-binding receptors and proteins, including oestrogen receptor alpha and sex hormone-binding globulin, which in turn interact with lipid metabolism genes, leading to reduced expression of lipid transporters [63-65]. These studies suggest a closer connection between early pubertal timing and lower HDL cholesterol [66,67]. However, the mediating role of HDL cholesterol in the relationship between pubertal timing and the risk of developing cardiovascular diseases has rarely been reported. Our results, at the genomic level, contribute to a better understanding of HDL-C as a mediator between early pubertal timing and increased risk of developing cardiovascular diseases.

For the ageing process, we found that early pubertal causally elevates alanine aminotransferase, HbA1c, and HDL-C levels and is associated with greater ageing. Previous multivariate genome-wide analyses of ageing-related traits revealed that HbA1c and HDL-C are novel targets for accelerated ageing and that blood glucose and lipid levels are risk factors for ageing and cognitive impairment [68,69]. In our study, the mvPuberty SNP-heritable cell type included pancreatic cells, suggesting that early pubertal timing may influence the level of HbA1c, which reflects the average blood glucose level over the last 90 days, by controlling pancreatic cell secretion to accelerate ageing, in line with previous findings [69]. Clinical and epidemiological

studies have reported an association between elevated alanine aminotransferase levels and accelerated ageing and increased cardiovascular morbidity [70,71]. The mechanism by which puberty onset affects alanine aminotransferase is unknown, but studies of hepatocyte damage have shown that an abnormal increase in sex hormones leads to increased hepatocyte damage in mice [72]. Therefore, the causal link between early pubertal timing and increased alanine aminotransferase levels, implying hepatocyte damage, may be related to a steep increase in sex hormone levels. We analysed common biomarkers as mediators of early puberty and multiple adverse health outcomes for the first time and reported that earlier puberty can cause abnormalities in blood glucose, lipids and liver function, which can lead to an increased incidence of adverse health outcomes. Our findings indicate that close monitoring of blood glucose, blood lipids and liver function in adolescents with early pubertal timing is more beneficial for their health later in life.

Supplementary Reference

1. Grotzinger, A.D. *et al.* Genomic structural equation modelling provides insights into the multivariate genetic architecture of complex traits. *Nat Hum Behav* 3, 513-525 (2019).
2. Rosoff, D.B. *et al.* Multivariate genome-wide analysis of aging-related traits identifies novel loci and new drug targets for healthy aging. *Nat Aging* 3, 1020-1035 (2023).
3. Karlsson Linner, R. *et al.* Multivariate analysis of 1.5 million people identifies genetic associations with traits related to self-regulation and addiction. *Nat Neurosci* 24, 1367-1376 (2021).
4. Mallard, T.T. *et al.* Item-Level Genome-Wide Association Study of the Alcohol Use Disorders Identification Test in Three Population-Based Cohorts. *Am J Psychiatry* 179, 58-70 (2022).
5. Watanabe, K., Taskesen, E., van Bochoven, A. & Posthuma, D. Functional mapping and annotation of genetic associations with FUMA. *Nature Communications* 8(2017).
6. Asimit, J.L., Hatzikotoulas, K., McCarthy, M., Morris, A.P. & Zeggini, E. Trans-ethnic study design approaches for fine-mapping. *Eur J Hum Genet* 24, 1330-6 (2016).
7. Kichaev, G. *et al.* Improved methods for multi-trait fine mapping of pleiotropic risk loci. *Bioinformatics* 33, 248-255 (2017).
8. Chen, W. *et al.* Fine Mapping Causal Variants with an Approximate Bayesian Method Using Marginal Test Statistics. *Genetics* 200, 719-36 (2015).
9. Wang, G., Sarkar, A., Carbonetto, P. & Stephens, M. A simple new approach to variable selection in regression, with application to genetic fine mapping. *J R Stat Soc Series B Stat Methodol* 82, 1273-1300 (2020).
10. Benner, C. *et al.* Prospects of Fine-Mapping Trait-Associated Genomic Regions by Using Summary Statistics from Genome-wide Association Studies. *Am J Hum Genet* 101, 539-551 (2017).
11. Weissbrod, O. *et al.* Functionally informed fine-mapping and polygenic

localization of complex trait heritability. *Nat Genet* 52, 1355-1363 (2020).
12. Feng, H. *et al.* Leveraging expression from multiple tissues using sparse canonical correlation analysis and aggregate tests improves the power of transcriptome-wide association studies. *PLoS Genet* 17, e1008973 (2021).
13. Hu, Y. *et al.* A statistical framework for cross-tissue transcriptome-wide association analysis. *Nat Genet* 51, 568-576 (2019).
14. Mancuso, N. *et al.* Probabilistic fine-mapping of transcriptome-wide association studies. *Nat Genet* 51, 675-682 (2019).
15. Zhang, J. *et al.* Plasma proteome analyses in individuals of European and African ancestry identify cis-pQTLs and models for proteome-wide association studies. *Nat Genet* 54, 593-602 (2022).
16. Lu, Z. *et al.* Improved multi-ancestry fine-mapping identifies cis-regulatory variants underlying molecular traits and disease risk. *medRxiv* (2024).
17. de Leeuw, C.A., Mooij, J.M., Heskes, T. & Posthuma, D. MAGMA: generalized gene-set analysis of GWAS data. *PLoS Comput Biol* 11, e1004219 (2015).
18. Elliott, A. *et al.* Distinct and shared genetic architectures of gestational diabetes mellitus and type 2 diabetes. *Nat Genet* 56, 377-382 (2024).
19. Cousminer, D.L. *et al.* Genome-wide association study of sexual maturation in males and females highlights a role for body mass and menarche loci in male puberty. *Hum Mol Genet* 23, 4452-64 (2014).
20. Liang, Y. *et al.* BrainXcan identifies brain features associated with behavioral and psychiatric traits using large scale genetic and imaging data. *medRxiv*, 2021.06.01.21258159 (2022).
21. Bulik-Sullivan, B.K. *et al.* LD Score regression distinguishes confounding from polygenicity in genome-wide association studies. *Nat Genet* 47, 291-5 (2015).
22. Zhou, S. *et al.* Investigating the shared genetic architecture of post-traumatic stress disorder and gastrointestinal tract disorders: a genome-wide cross-trait analysis. *Psychol Med* 53, 7627-7635 (2023).
23. Bradfield, J.P. *et al.* Trans-ancestral genome-wide association study of longitudinal pubertal height growth and shared heritability with adult health

outcomes. *Genome Biol* 25, 22 (2024).

24. Okasha, M., Gunnell, D., Holly, J. & Davey Smith, G. Childhood growth and adult cancer. *Best Pract Res Clin Endocrinol Metab* 16, 225-41 (2002).
25. Lope, V. *et al.* Perinatal and childhood factors and risk of breast cancer subtypes in adulthood. *Cancer Epidemiol* 40, 22-30 (2016).
26. Mikkola, T.M. *et al.* Childhood growth predicts higher bone mass and greater bone area in early old age: findings among a subgroup of women from the Helsinki Birth Cohort Study. *Osteoporos Int* 28, 2717-2722 (2017).
27. Cole, T.J. *et al.* Using Super-Imposition by Translation And Rotation (SITAR) to relate pubertal growth to bone health in later life: the Medical Research Council (MRC) National Survey of Health and Development. *Int J Epidemiol* 45, 1125-1134 (2016).
28. Stumper, A. *et al.* Correlates of Menarcheal Age in a Psychiatric Sample of Adolescents. *J Nerv Ment Dis* 212, 129-131 (2024).
29. Suresh, S., O'Callaghan, M., Sly, P.D. & Mamun, A.A. Impact of childhood anthropometry trends on adult lung function. *Chest* 147, 1118-1126 (2015).
30. Li, S. *et al.* Ferroptosis is a protective factor for the prognosis of cancer patients: a systematic review and meta-analysis. *BMC Cancer* 24, 604 (2024).
31. Gao, L. *et al.* Small Nucleolar RNAs as Diagnostic and Prognostic Biomarkers in Cancer: A Systematic Review and Meta-Analysis. *Technol Cancer Res Treat* 23, 15330338241245939 (2024).
32. Antonisamy, B. *et al.* Weight Gain and Height Growth during Infancy, Childhood, and Adolescence as Predictors of Adult Cardiovascular Risk. *J Pediatr* 180, 53-61 e3 (2017).
33. Romero, C. *et al.* Exploring the genetic overlap between 12 psychiatric disorders. *medRxiv*, 2022.04.12.22273763 (2022).
34. Dehestani, N. *et al.* "Puberty age gap": new method of assessing pubertal timing and its association with mental health problems. *Mol Psychiatry* 29, 221-228 (2024).
35. Kim, H.S. *et al.* Pubertal luteinizing hormone levels in children with chronic

kidney disease and association with change in glomerular filtration rate. *Pediatr Nephrol* 39, 1543-1549 (2024).

36. Meuwese, C.L. & Carrero, J.J. Chronic kidney disease and hypothalamic-pituitary axis dysfunction: the chicken or the egg? *Arch Med Res* 44, 591-600 (2013).
37. Deboer, M.D., Steinman, J. & Li, Y. Partial normalization of pubertal timing in female mice with DSS colitis treated with anti-TNF-alpha antibody. *J Gastroenterol* 47, 647-54 (2012).
38. Jee, Y.H., Jumani, S. & Mericq, V. The Association of Accelerated Early Growth, Timing of Puberty, and Metabolic Consequences in Children. *J Clin Endocrinol Metab* 108, e663-e670 (2023).
39. Hamlat, E.J. *et al.* Early life adversity predicts an accelerated cellular aging phenotype through early timing of puberty. *Psychol Med* 53, 7720-7728 (2023).
40. Li, Z., He, Y., Wang, D., Tang, J. & Chen, X. Association between childhood trauma and accelerated telomere erosion in adulthood: A meta-analytic study. *J Psychiatr Res* 93, 64-71 (2017).
41. Mounier, N. & Kutalik, Z. Bias correction for inverse variance weighting Mendelian randomization. *Genet Epidemiol* 47, 314-331 (2023).
42. Yourman, L.C. *et al.* Evaluation of Time to Benefit of Statins for the Primary Prevention of Cardiovascular Events in Adults Aged 50 to 75 Years: A Meta-analysis. *JAMA Intern Med* 181, 179-185 (2021).
43. Miura, K. *et al.* Relationship of blood pressure to 25-year mortality due to coronary heart disease, cardiovascular diseases, and all causes in young adult men: the Chicago Heart Association Detection Project in Industry. *Arch Intern Med* 161, 1501-8 (2001).
44. Barzilai, N., Huffman, D.M., Muzumdar, R.H. & Bartke, A. The critical role of metabolic pathways in aging. *Diabetes* 61, 1315-22 (2012).
45. Zuber, V., Colijn, J.M., Klaver, C. & Burgess, S. Selecting likely causal risk factors from high-throughput experiments using multivariable Mendelian randomization. *Nat Commun* 11, 29 (2020).

46. Dai, H. *et al.* Causal relationships between the gut microbiome, blood lipids, and heart failure: a Mendelian randomization analysis. *Eur J Prev Cardiol* 30, 1274-1282 (2023).
47. Cheng, G. *et al.* Beyond overweight: nutrition as an important lifestyle factor influencing timing of puberty. *Nutr Rev* 70, 133-52 (2012).
48. Chahal, R. et al. Girls' pubertal development is associated with white matter microstructure in late adolescence. Neuroimage 181, 659-669 (2018).
49. Bava, S. et al. Sex differences in adolescent white matter architecture. Brain Res 1375, 41-8 (2011).
50. Herting, M.M. et al. Longitudinal changes in pubertal maturation and white matter microstructure. Psychoneuroendocrinology 81, 70-79 (2017).
51. Jossinger, S., Mawase, F., Ben-Shachar, M. & Shmuelof, L. Locomotor Adaptation Is Associated with Microstructural Properties of the Inferior Cerebellar Peduncle. Cerebellum 19, 370-382 (2020).
52. Ardekani, S., Kumar, A., Bartzokis, G. & Sinha, U. Exploratory voxel-based analysis of diffusion indices and hemispheric asymmetry in normal aging. Magn Reson Imaging 25, 154-67 (2007).
53. Bisecco, A. et al. Fatigue in multiple sclerosis: The contribution of occult white matter damage. Multiple Sclerosis Journal 22, 1676-1684 (2016).
54. Pruden, S.M. & Levine, S.C. Parents' Spatial Language Mediates a Sex Difference in Preschoolers' Spatial-Language Use. Psychological Science 28, 1583-1596 (2017).
55. Jung, M. et al. Sex Differences in White Matter Pathways Related to Language Ability. Front Neurosci 13, 898 (2019).
56. Fex Svenningsen, A. & Kanje, M. Estrogen and progesterone stimulate Schwann cell proliferation in a sex- and age-dependent manner. J Neurosci Res 57, 124-30 (1999).
57. Baulieu, E.E. & Schumacher, M. Progesterone as a neuroactive neurosteroid, with special reference to the effect of progesterone on myelination. Steroids 65, 605-612 (2000).

58. Groenen, A.G., Halmos, B., Tall, A.R. & Westerterp, M. Cholesterol efflux pathways, inflammation, and atherosclerosis. *Crit Rev Biochem Mol Biol* **56**, 426-439 (2021).
59. Casula, M., Colpani, O., Xie, S., Catapano, A.L. & Baragetti, A. HDL in Atherosclerotic Cardiovascular Disease: In Search of a Role. *Cells* **10**(2021).
60. Dai, H. *et al.* Causal relationships between the gut microbiome, blood lipids, and heart failure: a Mendelian randomization analysis. *Eur J Prev Cardiol* **30**, 1274-1282 (2023).
61. Bhale, A.S., Meilhac, O., d'Hellencourt, C.L., Vijayalakshmi, M.A. & Venkataraman, K. Cholesterol transport and beyond: Illuminating the versatile functions of HDL apolipoproteins through structural insights and functional implications. *Biofactors* (2024).
62. Luo, H. *et al.* Prenatal caffeine ingestion induces transgenerational neuroendocrine metabolic programming alteration in second generation rats. *Toxicol Appl Pharmacol* **274**, 383-92 (2014).
63. Aydin, B. & Winters, S.J. Sex Hormone-Binding Globulin in Children and Adolescents. *J Clin Res Pediatr Endocrinol* **8**, 1-12 (2016).
64. Gao, H., Falt, S., Sandelin, A., Gustafsson, J.A. & Dahlman-Wright, K. Genome-wide identification of estrogen receptor alpha-binding sites in mouse liver. *Mol Endocrinol* **22**, 10-22 (2008).
65. Park, G. *et al.* Sex Hormone-Binding Globulin Is Associated with Obesity and Dyslipidemia in Prepubertal Children. *Children (Basel)* **7**(2020).
66. Constantino, D.B. *et al.* Effects of lighting patterns in pubertal development and metabolism of female wistar rats. *Physiol Behav* **243**, 113641 (2022).
67. Day, F.R. *et al.* Shared genetic aetiology of puberty timing between sexes and with health-related outcomes. *Nat Commun* **6**, 8842 (2015).
68. Mattishent, K. & Loke, Y.K. Bi-directional interaction between hypoglycaemia and cognitive impairment in elderly patients treated with glucose-lowering agents: a systematic review and meta-analysis. *Diabetes Obes Metab* **18**, 135-41 (2016).

69. Souza, J.G. *et al.* Diabetes, hemoglobin A1c, and cognitive performance in older adults: is there any impact of frailty? Evidence from the ELSI-Brazil study. *Braz J Med Biol Res* **57**, e12939 (2024).
70. Sohn, J. *et al.* Elevation of Serum Aminotransferase Levels and Future Risk of Death from External Causes: A Prospective Cohort Study in Korea. *Yonsei Med J* **56**, 1582-9 (2015).
71. Lee, T.H., Kim, W.R., Benson, J.T., Therneau, T.M. & Melton, L.J., 3rd. Serum aminotransferase activity and mortality risk in a United States community. *Hepatology* **47**, 880-7 (2008).
72. Shirakawa, T. *et al.* The impact of serum testosterone level to reflect age-related multi-organ functions. *Endocr J* **71**, 265-272 (2024).
