# Supplementary material for: Cross-trait multivariate GWAS confirms health implications of pubertal timing
Source: Nat Commun. 2025 Jan 18;16:799. doi: 10.1038/s41467-025-56191-4 (PMC11742396; doi:10.1038/s41467-025-56191-4)
Supplement: Supplementary file 3 — Description of Additional Supplementary Files [file 41467_2025_56191_MOESM3_ESM.pdf]

Description of Additional Supplementary Files

Supplementary Data 1

Puberty timing related phenotypes included in exploratory and final specifications of Genomic structural equation model (Genomic SEM). Phenotypes explored, i.e., age of voice break, age of first facial hair, age of menarche, pubertal height growth. Linkage disequilibrium score regression (LDSC or LDSR) statistics presented. Cohort demographics described.

Supplementary Data 2

Linkage disequilibrium score regression (LDSC) estimates of heritability andgenetic correlations of puberty timing-related phenotypes. Estimated using LDSCfunction and LD Scores calculated by Bulik-Sullivan et al. including only HapMap3SNPs with minor allele frequency (MAF) > 0.01; and standard errors estimated usinga block jackknife over SNPs.

Exploratory **Supplementary Data 3**

factor analysis (EFA). Implementing multivariable LDSC, then exploratory factor analysis (EFA) informing subsequent confirmatory factor analysis.

Supplementary Data 4

Confirmatory factor analysis (CFA) for common factor and two factor models. Fitting both common factor and two factor models indicated by EFA, implementedinGenomic SEM.

Supplementary Data 5

Summary of quality control (QC) filtering applied to GWAS summary statistics prior to multivariate GSEM analysis. Removing inadmissible alleles; variants witheffect values exactly 0 compromising matrix inversion necessary for Genomic SEM; variants with MAF < 0.01 prone to error as fewer samples within genotype cluster, with LD score standard errors that tend to high; multiallelics or duplicates; variants not matched to 1000 Genomes Phase 3 European reference panel.

Supplementary Data 6

Characterization and annotation of significant hits in multivariate puberty timing GWAS (mvPuberty). Independent significant SNPs identified. Lead SNPs and genomic risk loci defined. Functional consequences on genes (ANNOVAR), CADD score, RegulomeDBscore, 15 chromatin state (127 tissue/cell types) annotated.

Supplementary Data 7

Lead multivariate puberty timing GWAS (mvPuberty) SNPs annotated in the four input puberty timing related GWASs.

Supplementary Data 8

GWAS Catalog look-up of the multivariate puberty timing GWAS lead SNPs andindependent significant SNPs. Implemented using FUMA GWAS SNP2GENE(performed April 8, 2024).

Supplementary Data 9

GWAS Catalog look-up of the puberty timing GWAS 18 novel lead SNPs andindependent significant SNPs. Implemented using FUMA GWAS SNP2GENE(performed April 8, 2024).

Supplementary Data 10

Fine-mapping of multivariate puberty timing GWAS (mvPuberty) lead SNPs. Fine-mapping implemented to identify most plausible causal variants. SNPs containedwithin each 95% credible set for mvPuberty loci are listed, along with their inclusionprobability and functional annotation.

Supplementary Data 11

Sources of 27 lead SNPs with heterogeneity.

Supplementary Data 12

Shared variants form SCOUTJOY of mvPuberty with male and female Tanner stage. Boldfaced Pvalue indicates outliers identified for shared variants.

Supplementary Data 13

Regression slopes form SCOUTJOY of mvPuberty with male and female Tannerstage.

Supplementary Data 14

Transcriptome-wide association study (TWAS). TWAS implemented to integrate GWAS and gene expression datasets to identify gene-trait associations and prioritizecausal genes at GWAS loci.

Supplementary Data 15

Multi-marker analysis of genomic annotation (MAGMA) gene-based results. MAGMA implemented with data from GTEx (version 8) to perform gene-based andgene-set analyses. Mapping SNPs to 18,649 protein coding genes within 10 kb of leadSNPs accounting for LD between SNPs (using the1000G reference panel).

Supplementary Data 16

Gene-set enrichment analysis (GSEA) using MAGMA-derived genes. Gene-to-function (G2F) gene-set analyses implemented using genes identified withMAGMA to evaluate potential relationships between mvPuberty and mapped genes from MSigDB gene sets, including Reactome, and Gene Ontology (GO).

Supplementary Data 17

Cell-type enrichment analysis. To identify etiological cell types associated with mvPuberty, single-cell RNAsequencing (scRNA-seq) from Tabula Muris was integrated using CELLECT(CELL-type Expression-specific integration for Complex Traits). In CELLECT, MAGMA measures extent to which genetic

associations with a phenotype increase as a function of gene expression specificity for a given cell type, categorized following nomenclature used in original Tabula Muris study.

Supplementary Data 18

Proteome-wide association study (PWAS). PWAS implemented to integrate GWASand protein expression datasets to identify protein-trait associations and prioritize causal protein at GWAS loci.

Supplementary Data 19

LDSC analysis investigating the Genetic correlation between mvPuberty andmuti-traits.

Supplementary Data 20

Brain image-derived phenotypes correlation analysis for mvPuberty identifiedby BrainXcan.

Supplementary Data 21

Brain image-derived phenotypes casual analysis with mvPuberty identifiedbyBrainXcan.

Supplementary Data 22

Mendelian randomization analysis investigating the causal role of mvPubertyonmuti-traits.

Supplementary Data 23

Mendelian randomization analysis investigating the causal role of mvPubertyonserum biomarkers.

Supplementary Data 24

Mendelian randomization analysis investigating the causal role of mvPubertyongut microbiota.

Supplementary Data 25

Mendelian randomization analysis investigating the causal role of significant biomarkers on significant traits.

Supplementary Data 26

Ranking of models and Prioritization of causal serum biomarkers using the MR-BMA method.

Supplementary Data 27

Mendelian randomization analysis investigating the causal role of Parabacteroides on significant traits.

Supplementary Data 28

Mendelian randomization analysis investigating the causal role of dietary factors on mvPuberty.

Supplementary Data 29

Mendelian randomization analysis investigating the causal role of serumMicronutrients on mvPuberty.

Supplementary Data 30

Detailed information for polygenic Mendelian randomization adulthood muti-traits.

Supplementary Data 31

Detailed information for mediator effects Mendelian randomization biomarkers.

Supplementary Data 32

Detailed information for polygenic Mendelian randomization dietary factors.
