# Supplementary material for: Cross-trait multivariate GWAS confirms health implications of pubertal timing
Source: Nat Commun. 2025 Jan 18;16:799. doi: 10.1038/s41467-025-56191-4 (PMC11742396; doi:10.1038/s41467-025-56191-4)
Supplement: Supplementary file 36 — Reporting Summary [file 41467_2025_56191_MOESM36_ESM.pdf]

nature portfolio

Corresponding author(s): Jingyuan Xiong (jzx0004@tigermail.auburn.edu) and Guo Cheng (gcheng@scu.edu.cn)

Last updated by author(s): Dec 5, 2024

Reporting Summary

Nature Portfolio wishes to improve the reproducibility of the work that we publish. This form provides structure for consistency and transparency in reporting. For further information on Nature Portfolio policies, see our Editorial Policies and the Editorial Policy Checklist.

Statistics

For all statistical analyses, confirm that the following items are present in the figure legend, table legend, main text, or Methods section.

n/a	Confirmed	
☐	☒	The exact sample size (*n*) for each experimental group/condition, given as a discrete number and unit of measurement
☒	☐	A statement on whether measurements were taken from distinct samples or whether the same sample was measured repeatedly
☐	☒	The statistical test(s) used AND whether they are one- or two-sided<br>*Only common tests should be described solely by name; describe more complex techniques in the Methods section.*
☐	☒	A description of all covariates tested
☐	☒	A description of any assumptions or corrections, such as tests of normality and adjustment for multiple comparisons
☐	☒	A full description of the statistical parameters including central tendency (e.g. means) or other basic estimates (e.g. regression coefficient) AND variation (e.g. standard deviation) or associated estimates of uncertainty (e.g. confidence intervals)
☐	☒	For null hypothesis testing, the test statistic (e.g. *F*, *t*, *r*) with confidence intervals, effect sizes, degrees of freedom and *P* value noted<br>*Give P values as exact values whenever suitable.*
☒	☐	For Bayesian analysis, information on the choice of priors and Markov chain Monte Carlo settings
☒	☐	For hierarchical and complex designs, identification of the appropriate level for tests and full reporting of outcomes
☐	☒	Estimates of effect sizes (e.g. Cohen's *d*, Pearson's *r*), indicating how they were calculated

Our web collection on statistics for biologists contains articles on many of the points above.

Software and code

Policy information about availability of computer code

Data collection	No software was used for data collection
Data analysis	GenomicSEM v.0.0.5c: https://github.com/GenomicSEM/GenomicSEM;<br>TwoSampleMR v.0.6.2: https://mrcieu.github.io/TwoSampleMR/;<br>MendelianRandomization v.0.10.0: https://cran.r-project.org/web/packages/MendelianRandomization/index.html;<br>MRlap v.0.0.3: https://github.com/n-mounier/MRlap;<br>MR-BMA: https://github.com/JamesZ-Yang/MR-BMA;<br>FUSION TWAS: http://gusevlab.org/projects/fusion/;<br>UTMOST: https://github.com/Joker-Jerome/UTMOST;<br>FUSION PWAS: http://nilanjanchatterjeelab.org/pwas/;<br>FOCUS v.0.6.10: http://github.com/bogdanlab/focus/;<br>EasyFinemap v.0.4.6: https://jianhua-wang.github.io/easyfinemap/fine/;<br>FUMA and MAGMA v.1.5.2: https://fuma.ctglab.nl/;<br>BrainXcan: https://github.com/hakyimlab/brainxcan;<br>LD score regression: https://github.com/bulik/ldsc/wiki;<br>SCOUTJOY: https://github.com/aelliott08/SCOUTJOY;<br>R v.4.3.2, Python v.2.7 and Python v.3.9.

For manuscripts utilizing custom algorithms or software that are central to the research but not yet described in published literature, software must be made available to editors and reviewers. We strongly encourage code deposition in a community repository (e.g. GitHub). See the Nature Portfolio guidelines for submitting code & software for further information.

Data

Policy information about availability of data

All manuscripts must include a data availability statement. This statement should provide the following information, where applicable:

- Accession codes, unique identifiers, or web links for publicly available datasets
- A description of any restrictions on data availability
- For clinical datasets or third party data, please ensure that the statement adheres to our policy

All analyses were based on publicly available data. The mvPuberty GWAS data generated in this study are provided in the Zenodo database under accession code https://doi.org/10.5281/zenodo.14183879. The TWAS and PWAS data generated in this study are provided in the Source Data file. Summary-level statistics for age of menarche are available at: https://www.reprogen.org/; summary-level statistics for age of first facial hair are available at: https://broad-ukb-sumstats-us-east-1.s3.amazonaws.com/round2/; summary-level statistics for age of voice break are available at: https://broad-ukb-sumstats-us-east-1.s3.amazonaws.com/round2/; summary-level statistics for puberty height spurt are available at: http://egg-consortium.org/Pubertal_Growth/; summary-level statistics for Tanner stage are available at: http://egg-consortium.org/tanner-stage.html. GTEx weights for FUSION analyses are available at: http://gusevlab.org/projects/fusion/weights/sCCA_weights_v8_2.zip. Single-cell gene expression data from the Tabula Muris study are available at https://tabula-muris.ds.czbiohub.org/. Summary-level statistics used for Mendelian randomization are shown in Supplementary Data 30-32.

Research involving human participants, their data, or biological material

Policy information about studies with human participants or human data. See also policy information about sex, gender (identity/presentation), and sexual orientation and race, ethnicity and racism.

Reporting on sex and gender	We analyzed the effects of lead loci across sex using GWAS of Tanner stage. Sex of participants sourced for GWAS was determined based on self-report and Tanner scale. GWAS data used in our study is summary levels rather than individual levels.
Reporting on race, ethnicity, or other socially relevant groupings	We used the terms "European population, individuals or ancestry " to report genetic ancestry groups
Population characteristics	The population specific characteristics for the biobanks and population cohorts used in this study are described in Supplemental tables 1, 29, 30 and 31.
Recruitment	This study does not include recruitment of participants
Ethics oversight	Ethics oversight does not apply to this study

Note that full information on the approval of the study protocol must also be provided in the manuscript.

Field-specific reporting

Please select the one below that is the best fit for your research. If you are not sure, read the appropriate sections before making your selection.

☒ Life sciences ☐ Behavioural & social sciences ☐ Ecological, evolutionary & environmental sciences

For a reference copy of the document with all sections, see nature.com/documents/nr-reporting-summary-flat.pdf

Life sciences study design

All studies must disclose on these points even when the disclosure is negative.

Sample size	Effective sample size was 514,750 using mvPuberty summary statistics restricted to minor allele frequency (MAF) limits of 10% and 40% to produce stabler estimates.
Data exclusions	In multivariate GWAS, we removed SNPs with MAF<0.01 (to avoid error due to fewer samples within the genotype cluster). We created genetic instruments using SNPs at conventional genome-wide significance P < 5×10-8 clumped at linkage disequilibrium R2 = 0.001 (10,000 kb distance), using reference samples comprised of participants of European ancestry. F-statistics was calculated for the instruments for our exposure, which statistics generally exceeded the conventional cutoff of 10, suggesting minimal bias from weak instruments.
Replication	The results of the multivariate analysis were not directly replicated; instead, effect estimates were used to predict outcomes in independent cohorts
Randomization	Leveraged the random allocation of alleles through methods such as MR.
Blinding	The role in GWAS and post-GWAS studies is minimal due to the objective and automated nature of these analyses.

Reporting for specific materials, systems and methods

We require information from authors about some types of materials, experimental systems and methods used in many studies. Here, indicate whether each material, system or method listed is relevant to your study. If you are not sure if a list item applies to your research, read the appropriate section before selecting a response.

Materials & experimental systems

n/a	Involved in the study
☒	☐ Antibodies
☒	☐ Eukaryotic cell lines
☒	☐ Palaeontology and archaeology
☒	☐ Animals and other organisms
☒	☐ Clinical data
☒	☐ Dual use research of concern
☒	☐ Plants

Methods

n/a	Involved in the study
☒	☐ ChIP-seq
☒	☐ Flow cytometry
☒	☐ MRI-based neuroimaging

Plants

Seed stocks	This study is not conducted on plants.
Novel plant genotypes	This study is not conducted on plants.
Authentication	This study is not conducted on plants.
